# Structural and functional basis of antinociceptive action of χ-conotoxin AoIA at the noradrenaline transporter

**DOI:** 10.1038/s41594-026-01838-z

**Published:** 2026-07-21

**Authors:** Oliver J. V. Belleza, Heng Zhang, Helmut Schmidhammer, Tye I. Gonzalez, Cosmin I. Ciotu, Nataša Tomašević, Carlo Martin M. Ocampo, Jomari C. Fernando, Johannes Koehbach, Paula Schwarz, Mounaf Al Makhlouf, Gabor Tajti, Simon Hasinger, Nina Kastner, Orcun Avsar, Bernhard Retzl, Kathrin Jäntsch, Yi Jiang, Roland Hellinger, Michael J. M. Fischer, K. Johan Rosengren, Christian W. Gruber, Aaron Joseph L. Villaraza, Thomas Stockner, Mariana Spetea, H. Eric Xu, Harald H. Sitte

**Affiliations:** 1 Institute of Pharmacology, Center for Physiology and Pharmacology, Medical University of Vienna, Vienna, Austria; 2 State Key Laboratory of Drug Research, Shanghai Institute of Materia Medica, Chinese Academy of Sciences, Shanghai, China; 3 Department of Pharmaceutical Chemistry, Institute of Pharmacy and Center for Molecular Biosciences Innsbruck (CMBI), University of Innsbruck, Innsbruck, Austria; 4 School of Biomedical Sciences, The University of Queensland, Brisbane, Queensland Australia; 5 Institute of Physiology, Center for Physiology and Pharmacology, Medical University of Vienna, Vienna, Austria; 6 Institute of Chemistry, University of the Philippines – Diliman, Quezon City, Philippines; 7 Department of Molecular Biology and Genetics, Faculty of Arts and Sciences, Hitit University, Corum, Türkiye; 8 Lingang Laboratory, Shanghai, China; 9 Hourani Center for Applied Scientific Research, Al-Ahliyya Amman University, Amman, Jordan; 10 Center for Addiction Research and Science, Medical University of Vienna, Vienna, Austria

**Keywords:** Peptides, Cryoelectron microscopy, Membrane proteins

## Abstract

The χ-conotoxins are venom-derived peptides that specifically target the noradrenaline transporter (also known as norepinephrine transporter, NET). Regulation of noradrenergic signaling by NET affects neurophysiological processes, including pain. Therefore, the χ-conotoxin MrIA and its synthetic analogs have been previously investigated for their analgesic activity. Here we describe the synthesis and pharmacological characterization of χ-AoIA, a peptide that selectively inhibits NET with a higher potency compared to MrIA in in vitro radiotracer flux assays. Furthermore, we resolved the structure of the human NET:χ-AoIA complex by cryogenic electron microscopy, which revealed an atypical binding mode consisting of both the central binding site and the outer vestibule of the transporter. Lastly, χ-AoIA displays antinociceptive efficacy in a model of inflammatory pain after subcutaneous administration in mice. Our results demonstrate the efficacy of χ-AoIA as a highly selective ligand of NET and provide a mechanistic basis for its potential development as a nonopioid analgesic.

## Abstract (web summary)

Belleza, Zhang and colleagues showed that the χ-conotoxin χ-AoIA, a venom-derived peptide, reduces pain in mice by blocking the norepinephrine transporter more effectively than other similar peptides.

## Main

Cone snails are marine gastropods that live in tropical aquatic environments, widely recognized for the biochemical complexity of their venom. There are over 1,000 species of cone snails known to date (https://www.marinespecies.org). Each animal’s venom gland can contain up to 200 bioactive components, thus making them an abundant source of compounds with pharmacological potential^1–6^. Several decades of research have been dedicated to elucidating the chemical composition of cone snail venoms^7–9^. Most of these compounds are peptides and are, therefore, called ‘conopeptides’ or ‘conotoxins’, referring to their toxic effects. The cone snail can elicit excitotoxic shock, paralysis and sedation in their envenomed target, as a means to defend itself or facilitate prey capture^10^.

Most conotoxins are known to produce neurotoxic effects by acting on various ion channels, receptors and transporters in the nervous system^11^. Therefore, conotoxins are classified into pharmacological families, depending on the protein targets that define their biological activity^11,12^. Some have been developed into highly selective pharmacological probes used for the isolation of protein subtype-specific or isoform-specific activity (for example, the α-conotoxins for nicotinic acetylcholine receptors)^13^ or as potent drug leads for the treatment of pain (for example, the ω-conotoxin MVIIA, which is approved for clinical use as the intrathecally administered drug ziconotide)^14^. These advances highlight the important role of conotoxins in biomedical research^15^. Over the years, transcriptomic and proteomic techniques have accelerated the identification of peptides contained in various animal venoms, including from the cone snail^16^. As such, nucleotide and amino acid sequences of conotoxins are now effectively curated in online databases^17,18^. However, high-throughput approaches to biological screening have not advanced at a similar pace. As a result, many identified conotoxins do not have a confirmed molecular target. Indeed, among the 8000 known peptide sequences, more than 98% lack information on three-dimensional (3D) structure and functional activity^4^.

**Fig. 1:**
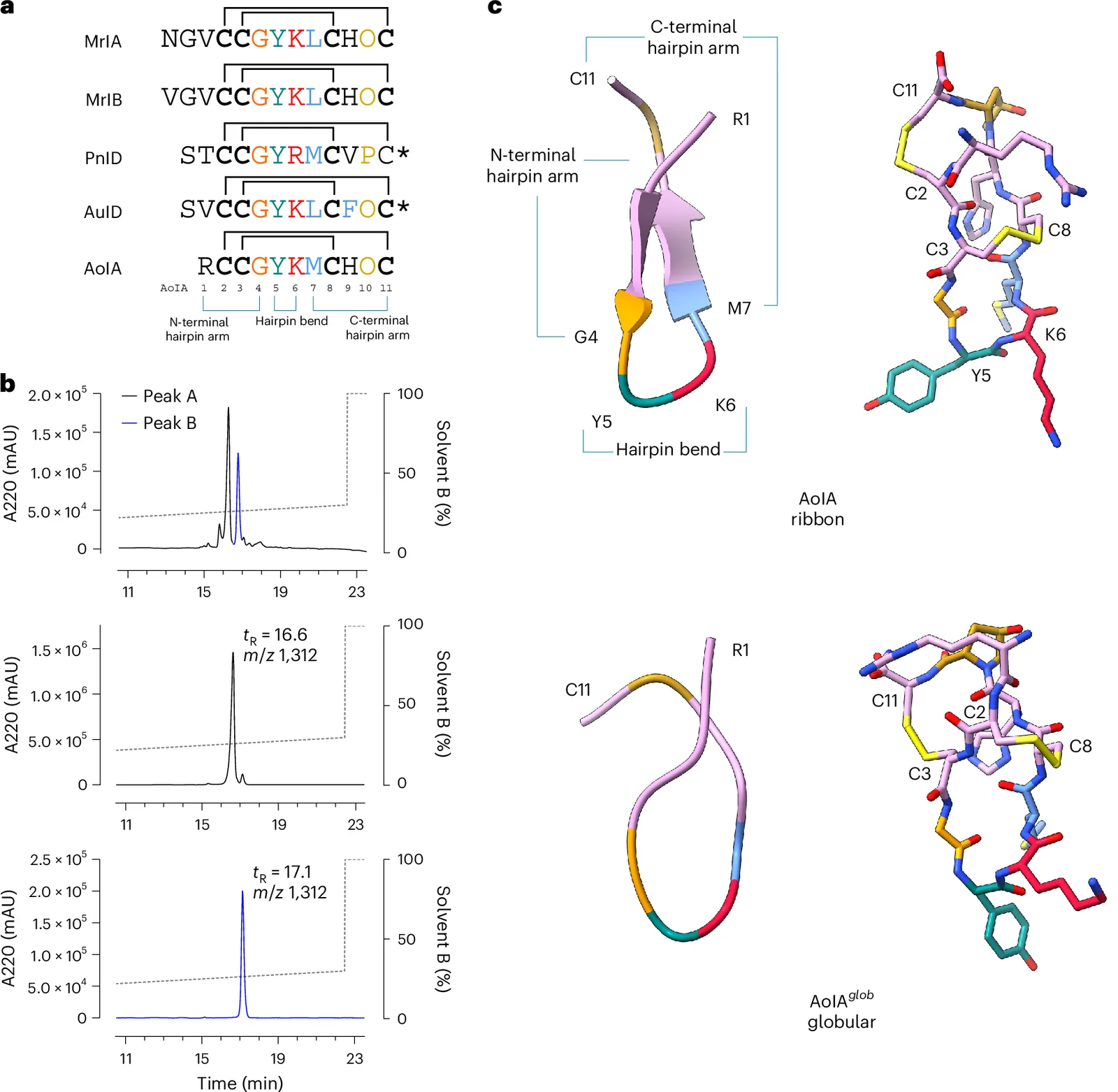
The peptide with the sequence RCCGYKMCHOC is a χ-conotoxin, AoIA. **a**, Sequence alignment of isolated χ-conotoxins with activity at NET: MrIA, MrIB, PnID, AuID and AoIA. Conserved residues within the first and second disulfide loops are colored using the ClustalX default color scheme. O, Hyp; *C-terminal amide. **b**, HPLC chromatograms show the two major isomeric products obtained from the synthesis of AoIA using nonselective oxidation: peaks A and B, with identical monoisotopic masses (experimental [M + H]^+^ *m*/*z* = 1,312) and different HPLC *t*_R_. Purified isomers (middle and bottom) were isolated from the crude product (top). **c**, NMR structural analysis of the two AoIA isomers reveals differences in disulfide connectivity. Shown here are representative structures selected from the 20 best models calculated using NMR data for AoIA^*glob*^ from peak A, with disulfides C2–C8;C3–C11 (globular conformation), and AoIA from peak B, with disulfides C2–C11;C3–C8, displaying the native ribbon conformation. AoIA adopts a hairpin structure consisting of the N-terminal hairpin arm (R1–G4), hairpin bend (Y5–K6) and C-terminal hairpin arm (M7–C11). Source data

Neurotransmitter transporters are a class of secondary active transport proteins that remove neurotransmitter molecules from the synaptic cleft^19^. This terminates the effect of neurotransmitters that are released following an action potential at their corresponding receptors. The noradrenaline transporter (also known as the norepinephrine transporter, NET) is a member of the SLC6 monoamine transporter (MAT) family, which also encompasses the transporters for dopamine (DAT) and serotonin (SERT). In 2001, the χ-conotoxins were shown to specifically target the NET, following the discovery of NET-inhibiting peptides χ-MrIA and χ-MrIB, both isolated from *Conus* *marmoreus*^20–22^. Most NET inhibitors are known for their antidepressant effects, yet some have also been found effective in treating chronic and neuropathic pain^23–25^. Remarkably, MrIA was also found to display antinociceptive activity in mice^21^. Soon afterward, it became the subject of extensive structure–activity relationship and optimization studies, which led to the development of Xen2174, a more potent and stable synthetic analog^26–29^. Xen2174 showed promise as an intrathecal analgesic drug in animals and in an open-label phase 1 clinical trial^30^. However, in a phase 2 trial with 25 subjects, it did not show a significant difference to placebo^30,31^. Despite the apparent therapeutic potential of χ-conotoxins^5^, MrIA and MrIB, along with their synthetic analogs, have remained the only members of this family with confirmed NET-specific activity for over two decades. Only recently were two new χ-conotoxins reported: PnID and AuID, isolated from *Conus* *pennaceus* and *Conus* *aulicus*, respectively^32,33^. Both peptides bear sequence similarities to MrIA and MrIB, most notably the cysteine framework, disulfide connectivity and the conserved amino acids found within the intercysteine disulfide loops (Fig. 1a). This has unlocked the potential expansion of the χ-conotoxin class by identifying structurally similar conotoxins isolated from other species. More recently, the structure of a synthetic analog of MrIA bound to NET, χ-MrIA^EM^, was solved using cryogenic electron microscopy (cryo-EM)^34^. Investigating the activity of χ-conotoxins at NET could further aid in their development as pharmacological tools and drug candidates for treating pain conditions.

Pain, as defined by the International Association for the Study of Pain, is an ‘unpleasant sensory and emotional experience associated or resembling that associated with actual or potential tissue damage’ (ref. ^35^). Chronic pain is a leading cause of disability worldwide and a major clinical, social and economic problem^36–38^. Opioid therapy is effective in treating moderate to severe pain but is limited by adverse effects, including constipation, respiratory depression and sedation, and by risks of analgesic tolerance, dependence and abuse^39,40^. Hence, new analgesic approaches are required.

Noradrenergic signaling has been described in detail elsewhere as a component of nociception and descending pain pathways^41–45^. Briefly, noradrenaline (or norepinephrine, NE) acts in the spinal cord as an inhibitory modulator of nociceptive processing through the activation of α_2_-adrenergic receptors (α_2_-AR). NE is taken up into the releasing neuron after having stimulated presynaptically and postsynaptically located G-protein-coupled receptors. Importantly, presynaptic α_2_-ARs negatively regulate NE release; however, an increase in synaptic NE stimulates postsynaptic α_2_-AR to inhibit calcium channels, which, in turn, reduces the release of excitatory neurotransmitters. On the basis of this mechanism, certain NET-targeting antidepressants are effective in treating coincident pain and depressive disorders^23–25^. Interestingly, other members of the SLC6 family have also gained attention (for example, glycine transporter 2) in the development of analgesic compounds^46,47^.

In this work, we identified a χ-conotoxin on the basis of its sequence similarity to MrIA and other reported χ-conotoxins. The peptide originally isolated by Gupta et al. from *Conus araneosus* with the sequence RCCGYKMCHOC, where O represents 4-hydroxyproline (Hyp), was found to possess the same disulfide connectivity as other χ-conotoxins^48^. Herein, we report its nuclear magnetic resonance (NMR) structure, investigate its activity at NET and confirm its pharmacological classification as a χ-conotoxin, hereafter named χ-AoIA (or simply AoIA). Moreover, the structural basis of its mechanism of action is established using a cryo-EM structure of the human NET:AoIA complex, which confirms AoIA as a ligand that occupies the entire outer vestibule of the transporter, including parts of the central substrate-binding site (S1). We also show the antinociceptive efficacy of AoIA in a mouse model of inflammatory pain, which demonstrates its potential as a nonopioid analgesic drug. The findings support further investigation of χ-conotoxins as leads for analgesic drugs and demonstrate the potential therapeutic implications of NET inhibition in the periphery.

## Results

### Chemical synthesis and structural and in vitro pharmacological characterization

**Extended Data Fig. 1:**
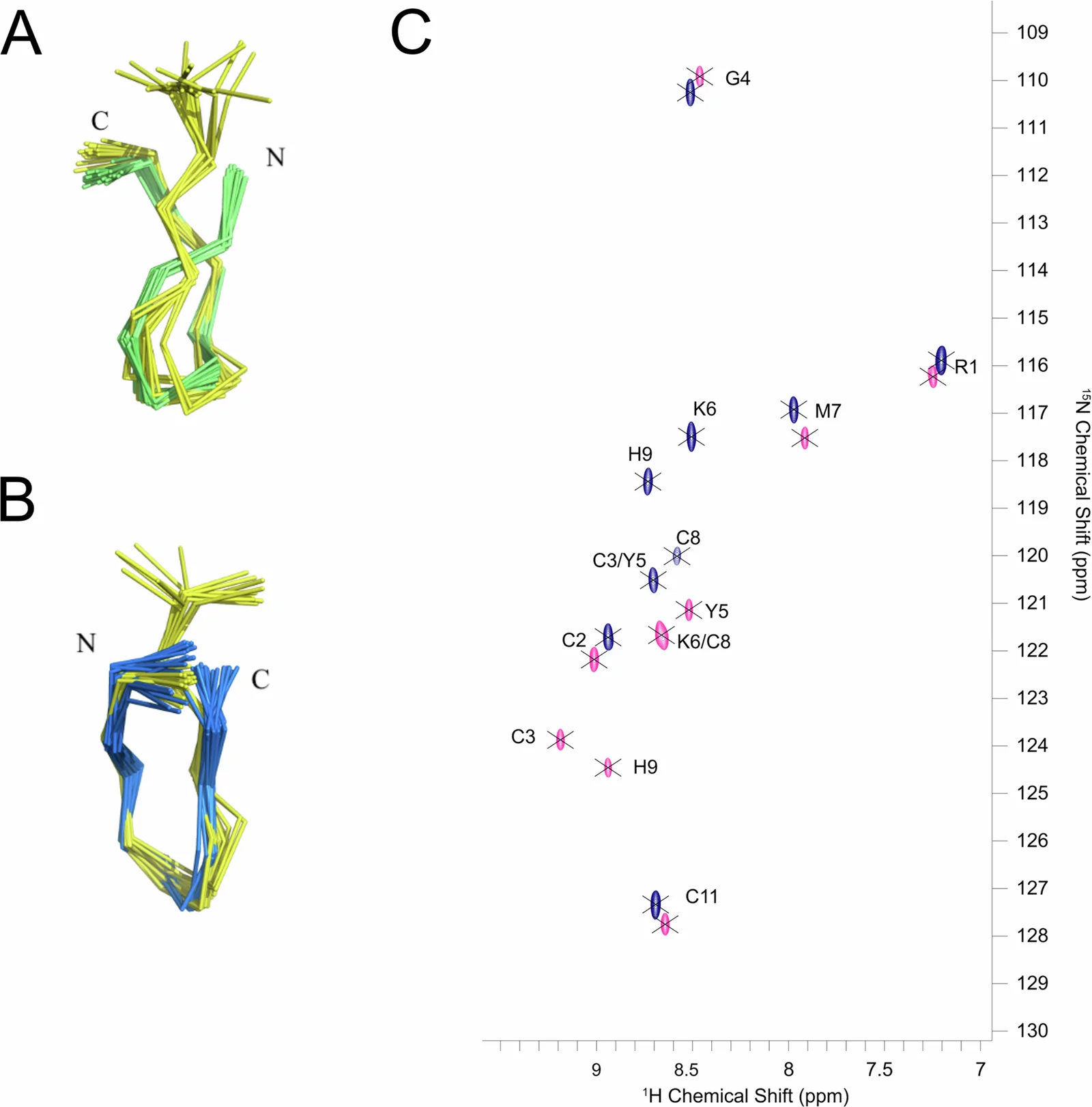
NMR solution structure of globular AoIA^*glob*^ and ribbon AoIA. Ensembles of the 20 best structure models of (**a**) globular AoIA^*glob*^, in green, and (**b**) ribbon AoIA, in blue, are superposed on the ensemble of MrIA (PDB:2EW4)^67^, shown in yellow. *N*- and *C*- termini are labelled. (**c**) Two dimensional ^15^N HSQC spectra of both disulfide isomers of AoIA recorded at 700 MHz. Cross peaks are labelled with single amino acid codes and residue numbers. The spectrum for the native disulfide isomer (I-IV, II-III) is shown in blue and the spectrum for the non-native isomer AoIA^*glob*^ (I-III, II-IV) is shown in pink.

Synthesis of AoIA was performed using automated solid-phase peptide synthesis. A conotoxin containing four cysteine residues can form its two disulfide pairs in three different ways: (1) I–III;II–IV, the ‘globular’ conformation; (2) I–IV;II–III, the ‘ribbon’ conformation; and (3) I–II;III–IV, the ‘bead’ conformation, with each Roman numeral representing the order in which the cysteine residues forming a disulfide bond appear in the amino acid sequence. A nonselective DMSO-assisted oxidative folding method yielded two major isomeric peptide products, observed as the two major peaks following high-performance liquid chromatography (HPLC) (Fig. 1b). Both peptides displayed nearly identical monoisotopic masses (1,312 Da) yet eluted separately. The first peak ‘peak A’ has an HPLC retention time (*t*_R_) of 16.6 min and elutes at 26.1% solvent B, while the second peak ‘peak B’ elutes at *t*_R_ = 17.1 min and 26.4% solvent B. Both variants were subjected to solution NMR spectroscopy and generated sharp lines with good dispersion, consistent with highly ordered structures. Sequential resonance assignment strategies using two-dimensional (2D) homonuclear and heteronuclear data recorded at natural abundance allowed for complete assignment of both isomers. Structural restraints derived from the data resulted in high-quality 3D structures (Extended Data Table 1 and Extended Data Fig. 1). Peak A was confirmed as the isomer with the globular conformation (I–III;II–IV), with disulfide bonds connecting C2–C8 and C3–C11. Meanwhile, peak B was the isomer with the ribbon conformation (I–IV;II–III) with disulfide bonds C2–C11 and C3–C8 (Fig. 1c). The peptide bearing the sequence of AoIA originally isolated from *C*. *araneosus* was reported to have a disulfide connectivity of C2–C11 and C3–C8 (ref. ^48^). Therefore, the later-eluting compound from peak B is the peptide AoIA that represents the native ribbon conformation.

The NMR structures show that AoIA, with the ribbon conformation, adopts a hairpin shape with antiparallel β-sheets stabilized by the C3–C8 disulfide bond. In contrast, the globular isomer, AoIA^*glob*^, is unable to adopt a similar secondary structure because of geometric constraints by the C2–C8 and C3–C11 disulfide bonds. The hairpin structure of AoIA can be subdivided into three regions (Fig. 1c): the N-terminal hairpin arm (R1–G4), the C-terminal hairpin arm (M7–C11) and the hairpin bend (Y5–K6) connecting the β-strands. The N-terminal and C-terminal hairpin arms are linked by the two disulfide bonds formed by C2–C11 and C3–C8, respectively. Comparison of the NMR structures to MrIA confirms a near-perfect overlap of the peptide backbone for the native AoIA ribbon isomer, whereas AoIA^*glob*^ only partly retains the χ-conotoxin pharmacophore (Extended Data Fig. 1). Synthesis of AoIA for further functional and structural studies was then subsequently performed using an orthogonal cysteine deprotection strategy to selectively form the desired disulfide connectivity: C2–C11 and C3–C8.

**Fig. 2:**
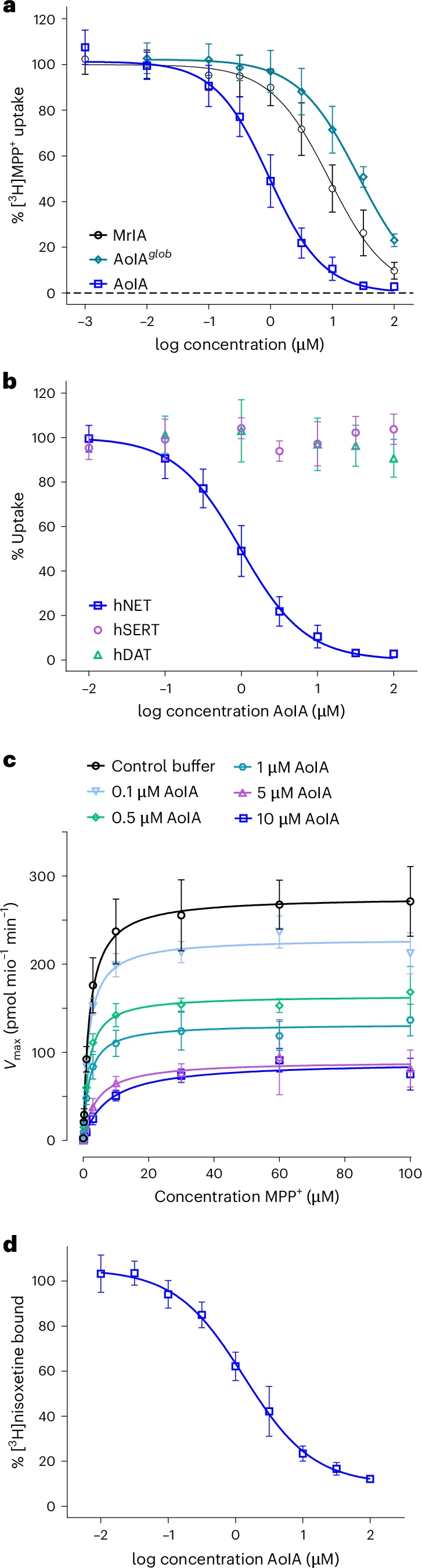
AoIA is a selective, mixed competitive/non-competitive inhibitor of NET. **a**, AoIA inhibits the uptake of [^3^H]-MPP^+^ by NET with a higher potency (IC_50_ = 1.06 ± 0.48 µM; *n* = 8 biological replicates) than the nonnative globular isomer AoIA^*glob*^ (IC_50_ = 23.72 ± 7.1; *n* = 3) and MrIA (IC_50_ = 10.9 ± 4.6 µM; *n* = 6). **b**, AoIA lacks activity at SERT and DAT up to the highest concentration tested (100 µM; *n* = 3) and is, therefore, a selective NET inhibitor. The NET inhibition curve taken from **a** is displayed for illustrative purpose. **c**, Increasing concentrations of AoIA result in a progressive decline in *V*_max_ of substrate uptake by NET, accompanied by an increase in *K*_m_ only at higher concentrations, which is indicative of a mixed competitive/noncompetitive mode of inhibition (concentrations [AoIA] in µM/*n*: 0/6, 0.1/3, 0.5/3, 1/4, 5/3 and 10/3). **d**, Radioligand displacement of [^3^H]-nisoxetine (1 nM) by increasing concentrations of AoIA shows that AoIA binds to NET with an average *K*_i_ = 1.30 ± 0.36 µM (*n* = 3). Error bars represent the s.d. Source data

The pharmacological characterization of AoIA as a χ-conotoxin was first confirmed by testing its biological activity in human embryonic kidney 293 (HEK293) cells stably expressing human NET. Both AoIA and the globular isomer AoIA^*glob*^ inhibited the uptake of the tritiated substrate [^3^H]-MPP^+^ by NET. Moreover, AoIA displayed greater inhibitory potency compared to the AoIA^*glob*^ isomer, with half-maximal inhibitory concentration (IC_50_) = 1.06 ± 0.48 µM (mean ± s.d.), as opposed to IC_50_ = 23.7 ± 7.1 µM (Fig. 2a and Table 1). According to the same assay, AoIA is about tenfold more potent at inhibiting the uptake of NET compared to MrIA (IC_50_ = 10.9 ± 4.6 µM). Affinity to NET was also confirmed by radioligand competition binding experiments using [^3^H]-nisoxetine in membrane fractions with *K*_i_ = 1.30 ± 0.36 µM (Fig. 2d). AoIA appears to inhibit the uptake of NET in a manner that could be interpreted as a mixed competitive/noncompetitive inhibition. This is substantiated by the observed incremental concentration-dependent decrease in *V*_max_ accompanied by an initial constant *K*_m_: going from 1.9 µM in the control buffer to 1.6–1.8 µM in the presence of 0.1–1 µM AoIA. This is followed by an increase in *K*_m_ values at higher concentrations (4.1 µM and 7.4 µM at AoIA concentrations of 5 µM and 10 µM, respectively) (Fig. 2c and Table 2). Furthermore, we demonstrate the selectivity of AoIA toward NET versus SERT and DAT. When tested in HEK293 cells stably expressing human SERT and DAT, AoIA did not inhibit substrate uptake up to the highest concentration tested (100 µM; Fig. 2b).

**Table 1:** Summary of IC_50_ values (µM) obtained from the inhibition of NET [^3^H]-MPP^+^ uptake by AoIA^*glob*^, AoIA and MrIA in stable HEK293 cell line

	AoIA^*glob*^	AoIA	MrIA
**Mean**	23.7	1.06	10.9
**s.d.**	7.07	0.48	4.61
***n***	3	8	6

**Table 2:** Uptake kinetics of [^3^H]-MPP^+^ by NET in the presence of increasing concentrations of AoIA

	Control buffer	0.1 µM AoIA	0.5 µM AoIA	1 µM AoIA	5 µM AoIA	10 µM AoIA
***V***_**max**_ **(pmol mio**^**−1**^ **min**^**−1**^**)**	276.5	229.2	164.4	132.0	90.06	89.32
*V*_max_ 95% CI	267.2–285.7	223.4–235.1	159.3–169.5	126.4–137.6	81.75–98.37	82.97–95.8
***K***_**m**_ **(µM)**	1.9	1.7	1.6	1.8	4.1	7.4
*K*_m_ 95% CI	1.6–2.2	1.4–1.9	1.3–1.9	1.4–2.2	2.4–5.8	5.3–9.6

HEK293 cells stably expressing hNET were incubated with increasing concentrations of MPP^+^ in the absence (control buffer) and presence of 0.1, 0.5, 1, 5 and 10 µM AoIA. Nonspecific uptake was determined in the presence of 50 µM GBR12909. The amount of MPP^+^ taken up by the cells (per million, mio) after 5 min was measured and analyzed. Data were fitted to the Michaelis–Menten equation to determine the kinetic parameters from *n* = 3 (0.1, 0.5, 5, 10 µM AoIA), 4 (1 µM AoIA) and 6 (control buffer) independent experiments.

**Fig. 3:**
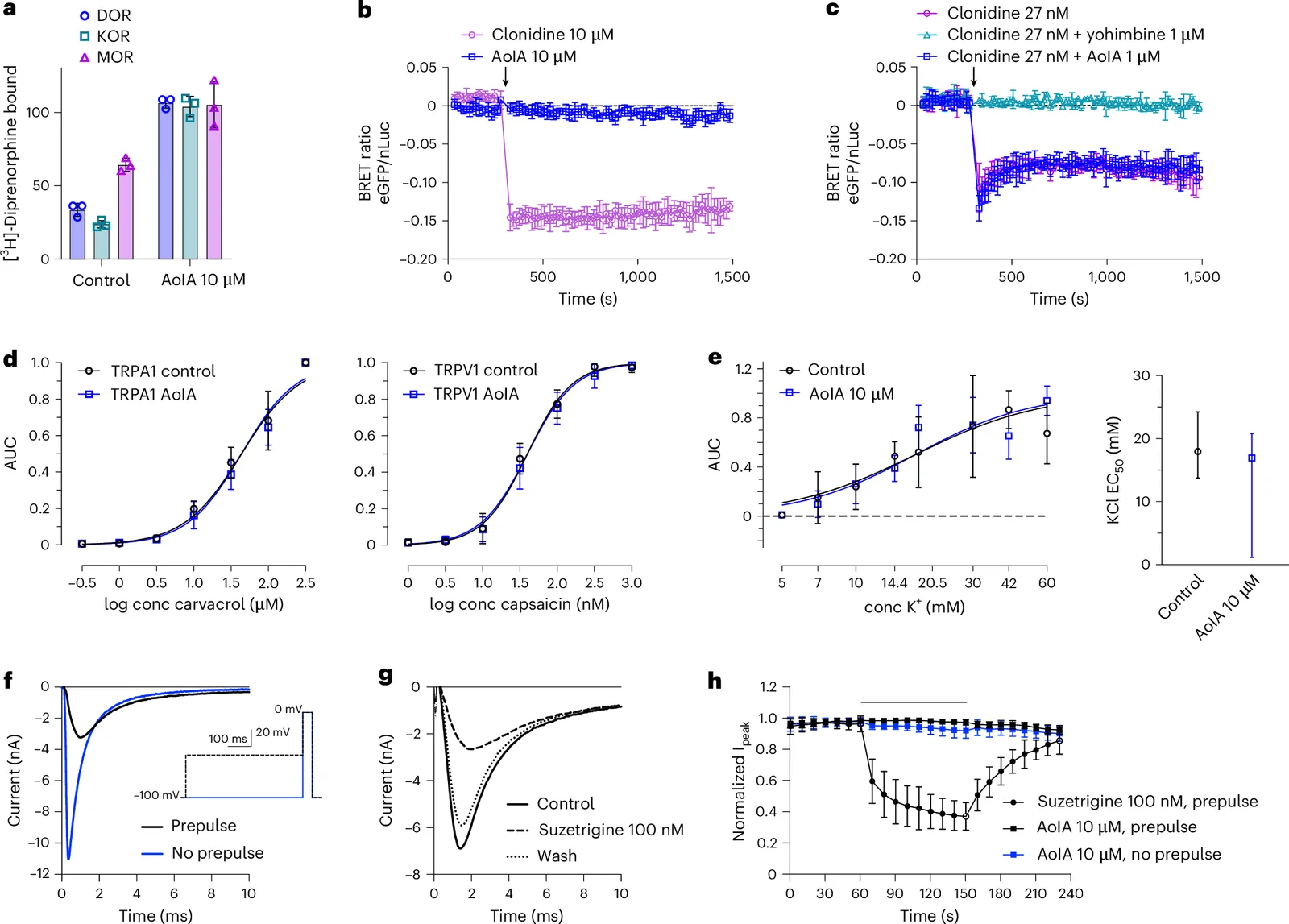
AoIA has no effect on key targets in nociception. **a**, Single-point radioligand displacement of [^3^H]-diprenorphine (1 nM) by AoIA (10 µM) at DOR, KOR and MOR. DADLE, dyn A and DAMGO (10 nM) were used as positive controls, respectively (*n* = 3 biological replicates ± s.d.). **b**, The effects of AoIA (10 µM) and the small-molecule agonist clonidine (10 µM) on Gα_i1_ dissociation at the α_2A_-AR expressed in HEK293 cells were investigated in BRET assays. Data are presented as the mean (*n* = 3) ± s.d. **c**, Additionally, cells were preincubated with the small-molecule antagonist yohimbine (1 µM) or AoIA (1 µM), followed by induction with a half-maximal effective concentration (EC_50_) of clonidine. The arrow represents the time of compound application (*t* = 300 s). Data are presented as the mean (*n* = 3) ± s.d. **d**, Effects of AoIA on TRPA1 and TRPV1 activation. Four-parameter logistic fits of the normalized area under the concentration–response curves (AUCs) for carvacrol (left; TRPA1-transfected cells, *n* = 6) and capsaicin (right; TRPV1-transfected cells, *n* = 3), following a 15-min pretreatment with 10 µM AoIA (blue) or vehicle (black). Data are presented as the mean ± s.d. **e**, AoIA effects on KCl depolarization of mouse dorsal root ganglia neurons. Left: four-parameter logistic fit of the normalized AUCs for the KCl response following a 15-min pretreatment with 10 µM AoIA or vehicle (*n* = 4). Data are presented as the mean ± s.d. Right: estimated KCl EC_50_ values with 95% CIs for the conotoxin treatment compared with the vehicle treatment. **f**, Representative current traces recorded from mouse DRG neurons without (blue line) or with a conditioning depolarizing prepulse to −50 mV for 500 ms (black line) to isolate slow-inactivating Na_V_ currents, followed by a test pulse to 0 mV for 40 ms (voltage protocol in inset). Data were recorded from the same cell and represent the first 10 ms of the test pulse. **g**, Representative traces of currents recorded with conditioning depolarizing prepulse before or upon application of Na_V_1.8 antagonist suzetrigine and after washout. **h**, Normalized peak currents obtained every 10 s. The black bar represents the application of suzetrigine or AoIA. Empty circles represent the time points shown in **g**. AoIA inhibited neither the largely Na_V_1.7-dependent sodium current nor the largely Na_V_1.8-dependent current after the prepulse. Data are presented as the mean ± s.d. (*n* = 7 per group). Source data

**Extended Data Fig. 2:**
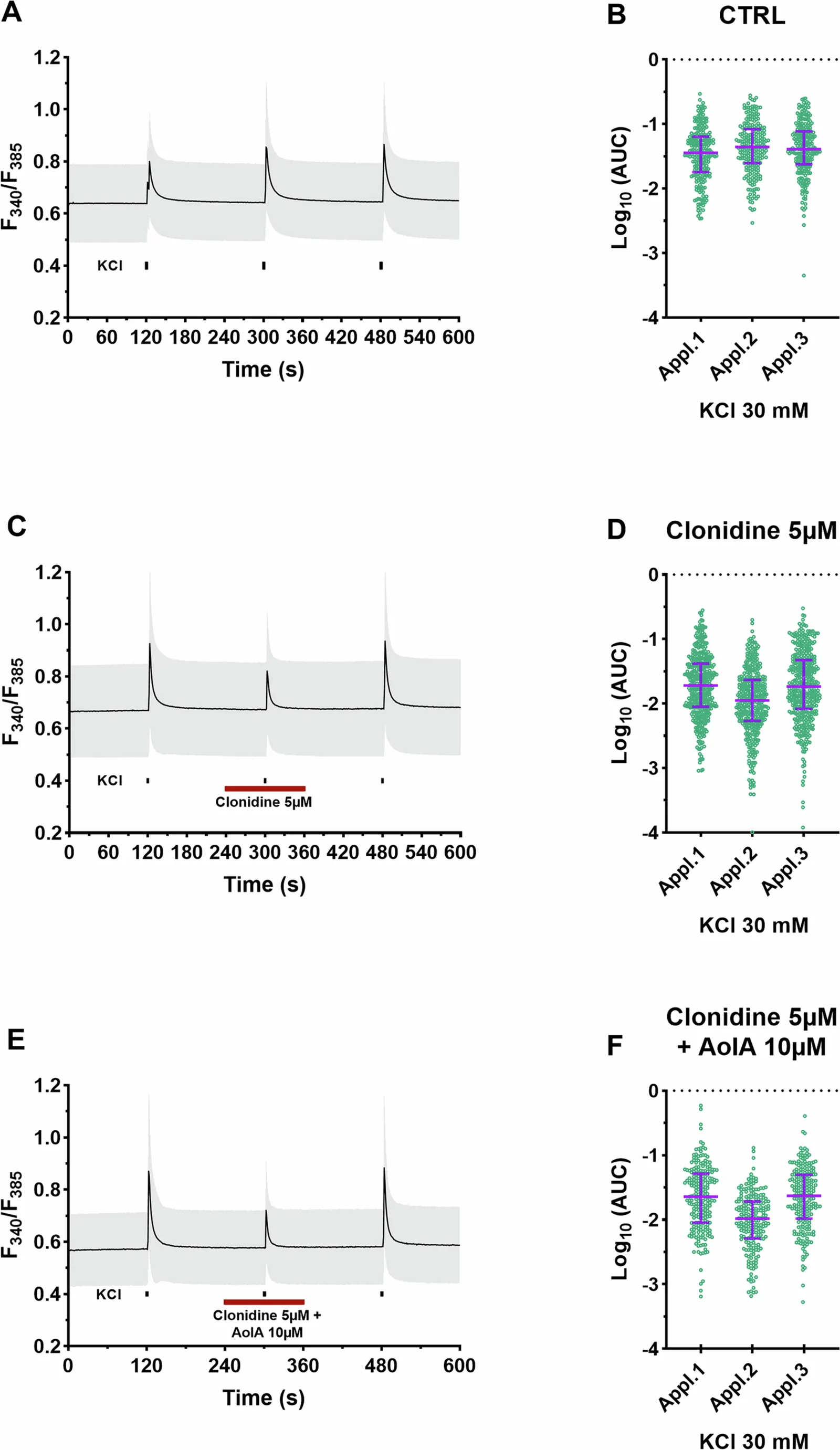
Assessment of possible off-target effects of AoIA on α2 -adrenergic receptors in DRG neurons. (**a**) Control application of KCl 30 mM for 1 s, with AUC values shown as mean ± SD in (**b**) *n* = 240 cells, 3 replicates. (**c**) Clonidine 5 µM is pre- and coapplied with the second KCl pulse (red bar), with AUC values shown as mean ± SD in (**d**) *n* = 438 cells, 4 replicates. (**e**) Clonidine 5 µM and AoIA are pre- and coapplied with the second KCl pulse (red bar), with AUC values shown as mean ± SD in (**f**) *n* = 230 cells, 2 replicates. Source data

The χ-conotoxin MrIA and its synthetic analog Xen2174 have been studied for their analgesic effects^26–29^. Accordingly, we also explored the possible effects of AoIA on a variety of targets involved in nociception. Firstly, we tested the binding of AoIA to the mu, delta and kappa opioid receptors (MOR, DOR and KOR, respectively) expressed in HEK293 cells. We confirmed through radioligand competition binding assays that AoIA (10 µM) had no affinity for these targets (Fig. 3a). To further investigate the possible adrenergic mechanism of AoIA, we also tested its effect on α_2__A_-adrenoceptors (α_2A_-ARs) expressed in HEK293 cells using bioluminescence resonance energy transfer (BRET) assays. AoIA displayed neither agonistic nor antagonistic effects on α_2A_-AR, as opposed to the control compounds clonidine and yohimbine (Fig. 3b,c). The lack of adrenergic effect we observed in HEK293 cells was also replicated in DRG neurons (Extended Data Fig. 2).

**Extended Data Fig. 3:**
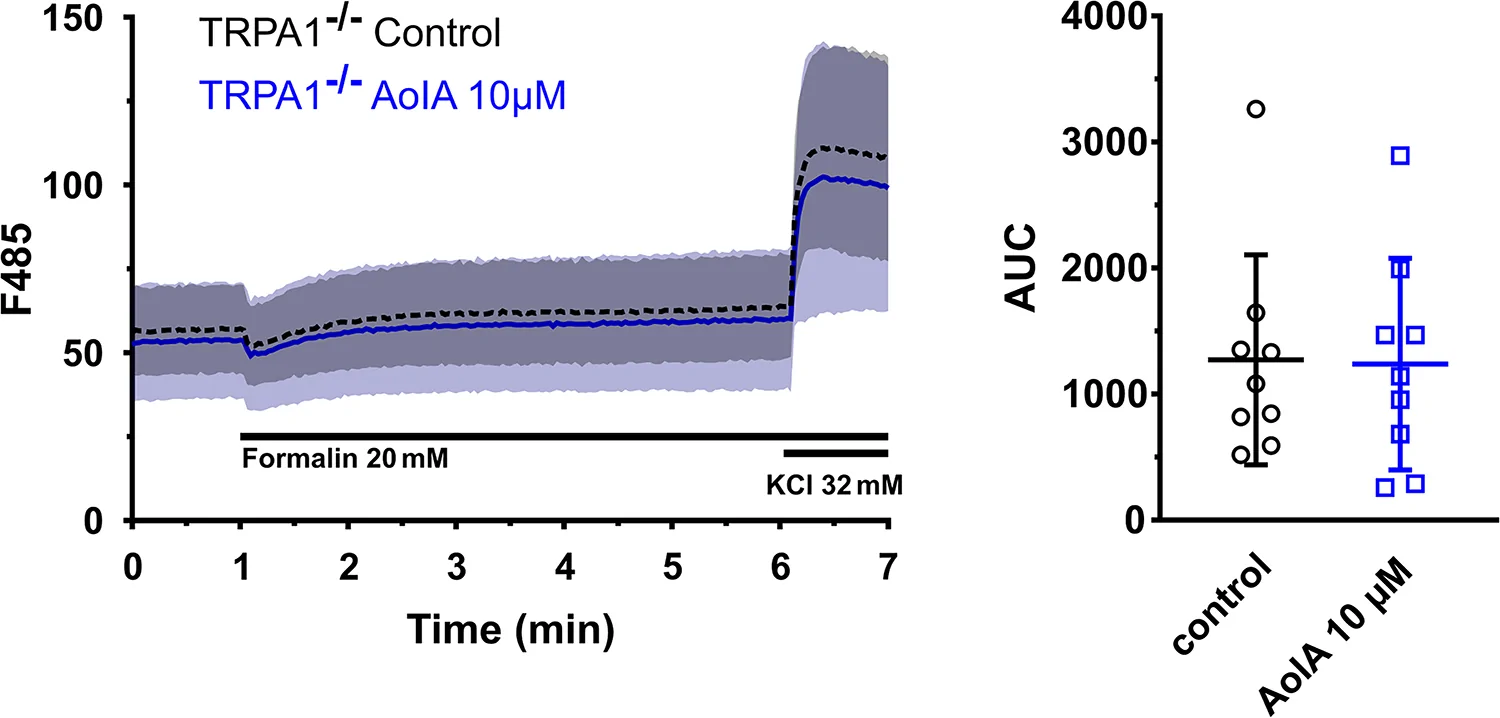
Assessment of possible off-target effects of AoIA on SERCA in TRPA1^−/−^ DRG neurons. Left: Intracellular calcium level traces measured by Calcium 6 microfluorimetry. Horizontal bars indicate the sequential addition of formaldehyde (20 mM) and potassium chloride (32 mM). AoIA (10 µM) was applied for 15 min before the start of the recordings. Right: Areas under the curve of the formaldehyde application (period 60–360 s) were similar to a vehicle-treated controls (*n* = 9 each). Error bars represent SD. Source data

Next, we tested for the effect of AoIA (10 µM) on the transient receptor potential (TRP) ion channels, TRPA1 and TRPV1 (Fig. 3d). We confirmed that AoIA did not affect the activity of TRPA1 or TRPV1 channels expressed in HEK293 cells, activated by their agonists carvacrol and capsaicin, respectively (Fig. 3d). To assess whether AoIA modulates the activity of voltage-gated calcium channels, we monitored intracellular calcium in DRG neurons across a range of potassium-induced depolarizations. Increasing extracellular potassium shifts the membrane potential in a Nernstian fashion to directly trigger the opening of voltage-gated calcium channels. Therefore, the overlapping dose–response curves of potassium-induced calcium entry indicate that AoIA does not modulate the voltage sensitivity of the low-voltage and high-voltage sensitive calcium channels expressed in sensory neurons (Fig. 3e). We also tested for the potential activity of AoIA on the sarco/endoplasmic reticulum calcium ATPase (SERCA). Formaldehyde inhibits SERCA, leading to TRPA1-independent cytoplasmic calcium elevation and slow endoplasmic calcium depletion^49–51^. Prior exposure to either AoIA or control solution resulted in a similar small increase in intracellular calcium by 20 mM formaldehyde, thus excluding SERCA as a target (Extended Data Fig. 3).

Whole-cell electrophysiology was used to investigate a potential effect of AoIA on voltage-gated sodium channels in DRG neurons. We investigated the effect of AoIA on net fast inward currents of sensory neurons^52^. Additionally, a −50 mV conditioning pulse preceding the test pulse was used to isolate slow-inactivating sodium currents^53^. AoIA inhibited neither the net sodium inward current, mainly carried by Na_V_1.7, nor the isolated slow-inactivating sodium currents, mainly carried by Na_V_1.8. The Na_V_1.8 inhibitor suzetrigine served as a positive control (Fig. 3f–h).

These results suggest that any potential analgesia elicited by AoIA would not result from direct interactions with opioid receptors, adrenergic receptors, TRPA1, TRPV1 and voltage-gated sodium and calcium channels, but would rather be mainly driven by its selective action at NET.

### Structural basis of the mechanism of action of AoIA at NET using cryo-EM

**Fig. 4:**
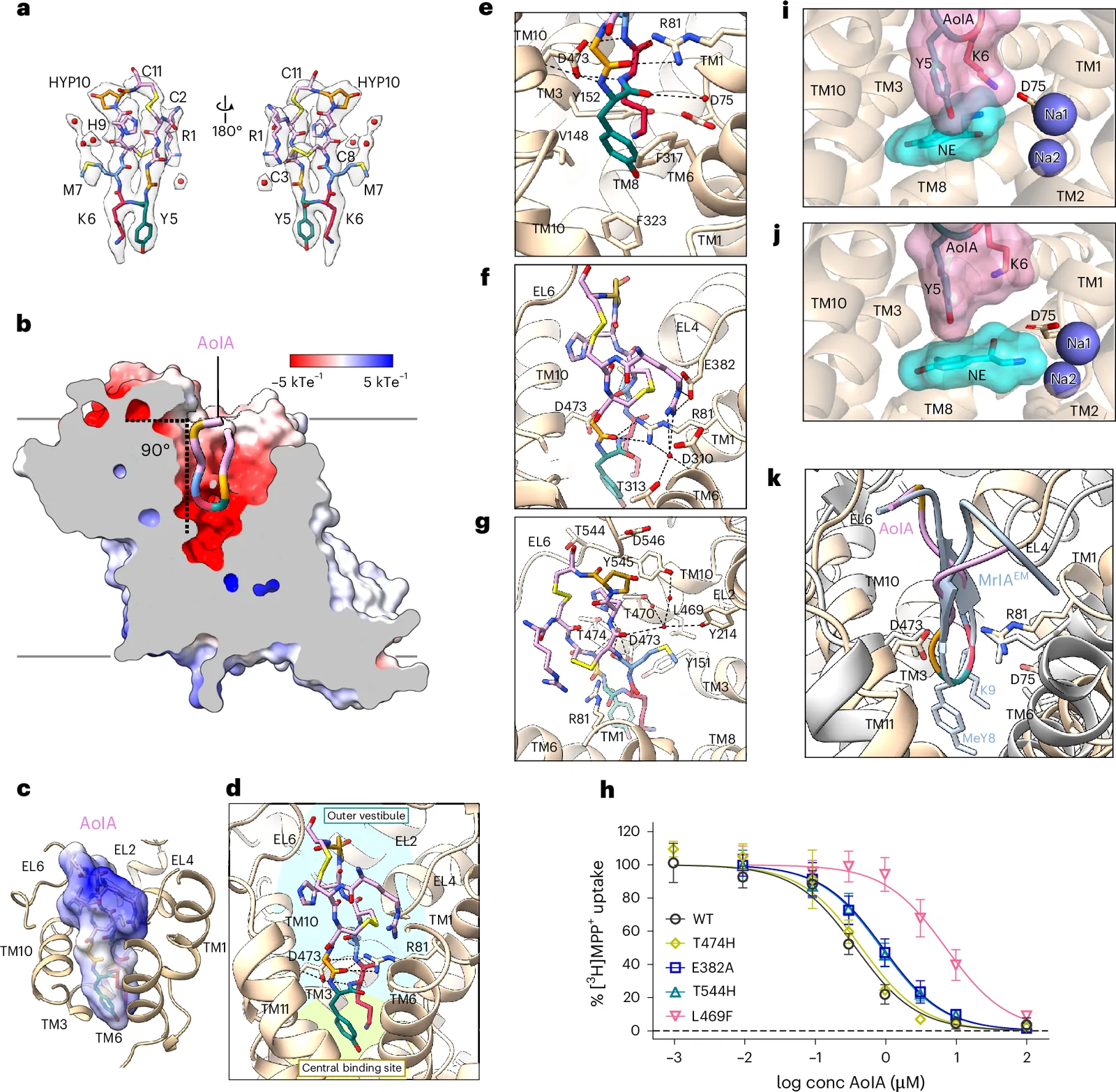
Structural insights into the binding pocket of AoIA in human NET. **a**, Electron density map of AoIA and water molecules, shown as a transparent solid surface. The AoIA molecule is depicted as colored sticks and water molecules are depicted as red spheres. **b**, Surface electrostatic potential map of AoIA-bound NET. A cross-sectional view highlights AoIA (pink) binding within the negatively charged pocket of NET, with the AoIA molecule oriented perpendicular to the membrane plane. **c**, The AoIA molecule is shown with a surface electrostatic potential showing complementarity to its binding pocket at NET. **d**, The overall binding pocket for the χ-conotoxin AoIA in NET consists of two parts: the outer vestibule and the central binding site (S1). The salt-bridge-forming residues D473^TM10^ and R81^TM1^ stabilize the peptide backbone, allowing for Y5^AoIA^ and K6^AoIA^ to reach into the S1 site. **e**, The hairpin bend of AoIA consisting of Y5^AoIA^ and K6^AoIA^ interacts with the S1 site through polar and hydrophobic interactions. **f**, The N-terminal hairpin arm (R1^AoIA^–G4^AoIA^) primarily engages with TM1, TM6 and EL4 of NET, with T313^TM6^ stabilizing R1^AoIA^ through water-mediated interactions and E382^EL4^ forming polar interactions with R1^AoIA^. **g**, The C-terminal hairpin arm (M7^AoIA^–C11^AoIA^) interacts with TM3, EL2, TM10 and EL6 of NET mainly through a network of water-mediated hydrogen bonds. Residues and water molecules are shown as sticks, with hydrogen bonds and salt bridges represented by dashed lines. **h**, Activity of AoIA in HEK293 cells transiently expressing wild-type (WT) NET and NET mutants. The potency of AoIA (IC_50_ = 0.39 ± 0.02 µM; *n* = 3 biological replicates) is significantly reduced in the E382A, T544H and L469F mutants, with IC_50_ = 0.89 ± 0.25 µM (*n* = 4, *P* = 0.0213), 0.90 ± 0.05 µM (*n* = 3, *P* = 0.000072) and 7.73 ± 2.3 µM (*n* = 3, *P* = 0.0056), respectively. This potency is retained in the T474H mutant (IC_50_ = 0.43 ± 0.03 µM; *n* = 3, *P* = 0.873). Unpaired two-tailed *t*-tests were performed to evaluate the differences in IC_50_ values compared to WT NET. Data are shown as the mean ± s.d. **i**, The structure of AoIA-bound NET is shown together with NE as observed in the NE-bound occluded structure of NET (PDB 8ZPB)^55^. NET is shown in beige, the semitransparent surface of AoIA is shown in red and the semitransparent surface of NE is shown in cyan. The nitrogen atoms of NE and K6^AoIA^ overlap and, thus, occupy the same space. **j**, Representative hypothetical structure of NET bound to AoIA and NE at the end of a simulation. The structural overlap of NE and AoIA was resolved in the simulation but resulted in displacements of NE, K6^AoIA^ and Na1, and a breaking of the key salt bridges of K6^AoIA^ stabilizing AoIA. This shift reflects the incompatibility of simultaneous NE and AoIA binding. **k**, The structure of NET:AoIA (beige) is superposed with the structure of NET with χ-MrIA^EM^ bound (gray; PDB 8WTW)^34^ to display identical binding to NET of AoIA (pink) and χ-MrIA^EM^ (blue-gray). Source data

**Extended Data Fig. 4:**
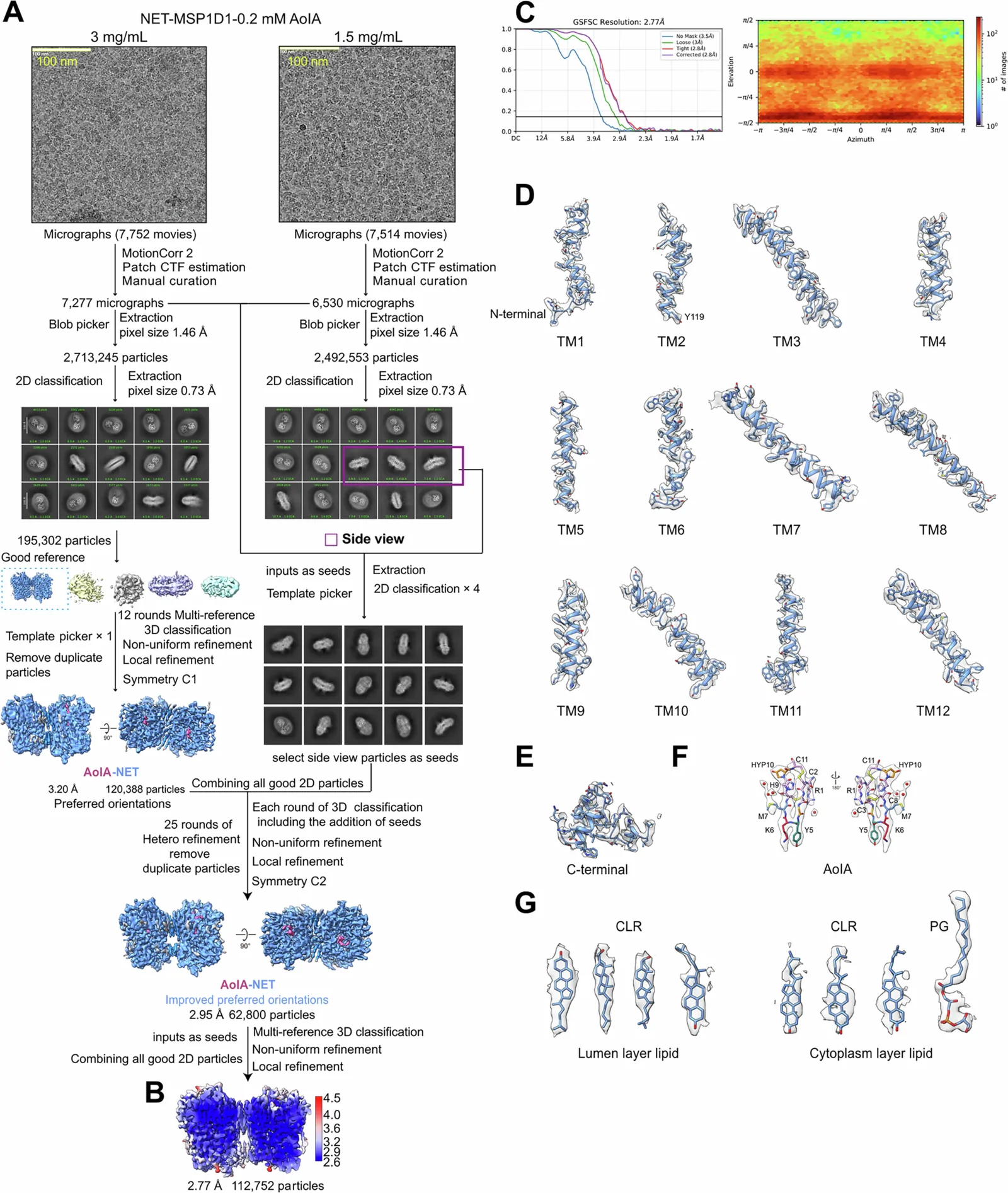
Cryo-EM data processing of NET bound to AoIA. (**a**), Data processing workflow of the NET-AoIA complex. The scale bar located at the upper left in the Representative cryo-EM micrographs of the NET-AoIA complex. (**b**), The local resolution of the sharpened map of the NET-AoIA complex is calculated by Cryosparc4.3.1. (**c**), Gold-standard Fourier shell correlation (FSC) curve for the 3D refinement and angular distribution of the particles used for final reconstitution of the NET-AoIA complex. (**d**), EM densities of TM helices of the NET. (**e**), EM densities of C-terminus of the NET. (**f**), EM densities of AoIA and water molecules. (**g**), EM densities of cholesterols and lipids at the dimer interface.

To further investigate the binding mode of AoIA at NET, we determined the structure of NET in complex with AoIA using cryo-EM. Lipid nanodisc and single-particle cryo-EM techniques were used to resolve the structure of the NET:AoIA complex. The overall resolution of the map is 2.77 Å. This high-resolution map allows for unequivocal assignment of the side chains for both NET and AoIA (Fig. 4, Extended Data Fig. 4 and Extended Data Table 2). Consistent with previous analyses, the NET:AoIA complex forms a homodimer mediated by cholesterol molecules and other lipids (Extended Data Fig. 4). The arrangement of cholesterol aligns with earlier reports^54^. However, unlike prior observations, this structure lacks densities corresponding to phosphatidylinositol at the cytoplasmic dimer interface. Instead, it reveals a density compatible with a lipid molecule, which is LPG 18:2 (1-(9*Z*,12*Z*-octadecadienoyl)-glycero-3-phospho-(1′-*sn*-glycerol)). Each protomer in the dimer combining with AoIA exhibited an outward-open conformation.

The high-resolution structure also reveals numerous water molecules surrounding AoIA within its binding pocket (Fig. 4a). The overall orientation of the NET-bound AoIA is perpendicular to the membrane plane and the electrostatic potential of NET is complementary to the charge distribution of AoIA (Fig. 4b,c). The conformation of AoIA observed from its NMR solution structure resembles a preformed bound state, limiting the energy penalty associated with induced-fit binding. The AoIA-binding site occupies the entire outer vestibule of NET in the outward-open conformation and also reaches into the S1 pocket (Fig. 4d). The water molecules mediate the interaction between AoIA and NET through a hydrogen-bond network and fill the remaining space in the outer vestibule.

In the occluded conformation of the NE-bound NET (PDB 8ZPB)^55^, R81^TM1^ and D473^TM10^ form the predicted salt bridge that is conserved across the SLC6 transporter family. Substitution of either residue was previously shown to block transport in MATs^56–58^. In the NET:AoIA complex, R81^TM1^ and D473^TM10^ precisely position AoIA in the outer vestibule by stabilizing it through strong salt bridges from either side (Fig. 4d). Specifically, residues R81^TM1^ and D473^TM10^ interact with the backbone around the hairpin bend of the peptide (Y5^AoIA^ and K6^AoIA^). Meanwhile, a key feature of the NET:AoIA complex is the interaction of K6^AoIA^ with NET. While the backbone position of K6^AoIA^ is determined by the interactions with R81^TM1^ and D473^TM10^, this conotoxin side chain is located at the tip of its β-strand–turn–β-strand structure and reaches into the S1 site (Fig. 4d,e). The positive charge of K6^AoIA^ overlaps with the position of the positive charge of the amino group of NE observed in the NE-bound NET structure (PDB 8ZPB)^55^ (Fig. 4i). Residues D75^TM1^ and Y152^TM3^ in the S1 site form a salt bridge and polar interactions with K6^AoIA^, while hydrophobic interactions also occur between V148^TM3^, F317^TM6^, F323^TM6^ and Y5^AoIA^ of the bend (Fig. 4e). AoIA keeps the outer hydrophobic gate of NET open by placing the side chain of Y5 to the position that F317^TM6^ would assume in the closed state.

The N-terminal hairpin arm of AoIA (R1^AoIA^–G4^AoIA^) primarily engages with R81^TM1^, E382^EL4^ and T313^TM6^. R1^AoIA^ forms multiple salt bridges with E382^EL4^ that, along with water-mediated interactions, stabilize the peptide backbone (Fig. 4f). The C-terminal hairpin arm (M7^AoIA^–C11^AoIA^) mainly interacts with TM3, EL2, TM10 and EL6 of NET. These interactions can be divided into two components: (1) one involving Y214^EL2^, T470^TM10^ and Y545^EL6^, which form a hydrogen-bond network with M7^AoIA^ and C8^AoIA^ through multiple water molecules, and (2) the other involving D473^TM10^ and T474^TM10^, which establish a hydrogen-bond network with H9^AoIA^ of the C-terminal hairpin arm, also mediated by a water molecule. Residue L469^TM10^ also likely forms a hydrophobic interaction with M7^AoIA^ (Fig. 4g).

Similar binding interactions can be found in the cryo-EM structure of NET with χ-MrIA^EM^ bound (PDB 8WTW)^34^. Superposing the structures of NET:AoIA and NET:χ-MrIA^EM^ reveals that both peptides occupy the same binding site in the transporter (Fig. 4k). Residues R81^TM1^ and D473^TM10^ form the same hydrogen bonds with the backbone of χ-MrIA^EM^ around the region that creates a hairpin bend similar to AoIA. This region of χ-MrIA^EM^ also contains a positively charged lysine residue that inserts itself into the S1 site to form a salt bridge with D75^TM1^, which, along with a methylated tyrosine, forms hydrophobic interactions with the hydrophobic residues in this pocket^34^.

On the basis of the evaluation of the NET:AoIA complex, amino acids that are essential to binding AoIA were identified, which could allow for functionally verifying the peptide–protein interactions with minimal interference with the mechanics of NET. To confirm the essential binding partners of AoIA in NET, we performed single-point substitutions of the amino acids in NET with either alanine or their corresponding residues in DAT. The measured IC_50_ of AoIA in HEK293 cells transiently transfected with NET was lower (IC_50_ = 0.39 ± 0.02 µM, mean ± s.d.) compared to that from the stable cell line. This potency was retained in the T474H mutant (IC_50_ = 0.43 ± 0.03 µM). On the other hand, the E382A, T544H and L469F mutants produced a rightward shift of the inhibition curve for AoIA, which indicates a ~2-fold loss of inhibitory activity for E382A (IC_50_ = 0.89 ± 0.25 µM) and T544H (IC_50_ = 0.90 ± 0.05 µM) and a 20-fold loss for L469F (IC_50_ = 7.73 ± 2.3 µM) (Fig. 4h). This validates the importance of E382^EL4^, T544^EL6^ and L469^TM10^ for the formation of a suitable binding mode of AoIA within NET.

To verify that binding of AoIA and NE is competitive because of structural overlap, we first created a hypothetical model in which we combined the AoIA-bound NET and the NE-bound NET structures (PDB 8ZPB)^55^ (Fig. 4i). This hypothetical structure shows that the positively charged nitrogen atoms of NE and of K6^AoIA^ occupy the same space, interacting with D75^TM1^ and with the helical dipole of TM6. To further ascertain the competitive nature of NE and AoIA binding by spatial overlap, we carried out molecular dynamics (MD) simulations. The final hypothetical structure of the MD simulation (Fig. 4j) reflects the competitive nature as it leads to a high-energy conformation in which several key features of high-affinity binding to NET are broken. Multiple simulations, starting from the hypothetical structure of NE-bound and AoIA-bound NET, consistently resulted in the displacement of NE from its binding pocket, which moved so far toward the sodium-binding site to displace Na1, pushing it toward Cl^−^ and N350^TM7^. In parallel, K6^AoIA^ relocated from its key interactions with D75^TM1^ and the helical dipole of TM6 toward TM3, resulting in a breaking of critical charge interactions between AoIA and NET.

**Fig. 5:**
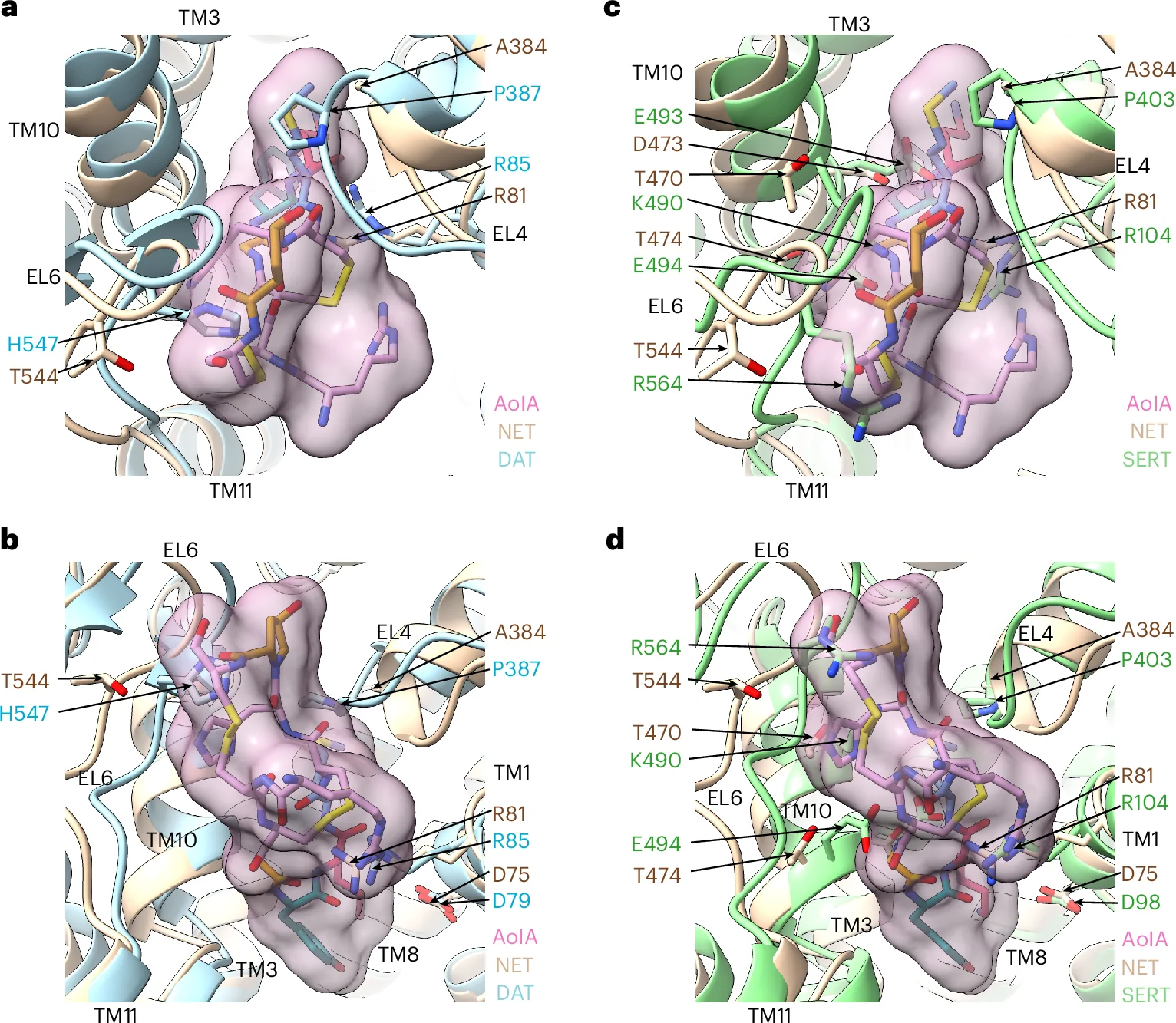
Structural comparisons of NET:AoIA to other MATs. **a**,**c**, Binding pocket superposition of NET:AoIA (beige) and DAT:cocaine (**a**; light blue; PDB 9EO4)^59^ and SERT:paroxetine (**c**; light green; PDB 6VRH)^60^, viewed from above. Residues A384^EL4^ and T544^EL6^ of NET are replaced with bulkier residues in DAT and SERT, extending EL4a and EL6 farther into the vestibule, which results in a steric overlap with AoIA. **b**,**d**, Structural clamp fixing of AoIA between the EL4a–EL4b loop and EL6 in DAT (**b**) and SERT (**d**), viewed from the side. Residues K490 and E494 in SERT overlap with AoIA, destabilizing the AoIA peptide backbone. AoIA is shown as colored sticks and a pink surface, with residues labeled in colors corresponding to the transporters: NET in brown, DAT in blue and SERT in green.

A comparison of the NET, DAT and SERT structures may elucidate how AoIA specifically inhibits the transport activity of NET and help explain why SERT and DAT are not affected, despite having 50% and 67% sequence identity (Extended Data Fig. 7). Such differences can be observed by superimposing our NET:AoIA structure with published structures of human DAT (PDB 9EO4)^59^ and SERT (PDB 6VRH)^60^ (Fig. 5). AoIA stabilized NET in an outward-open conformation, which is very similar to the conformation observed for DAT and SERT. The scaffold domain and the bundle domain assume an identical conformation, within experimental uncertainty. Small differences can be observed for EL4a, EL6 and TM12, whereby the AoIA-bound structure is ~1 Å more open than cocaine-bound DAT and paroxetine-bound SERT^59,60^. Many residues in contact with AoIA are shared across DAT, NET and SERT, as NET and DAT share a sequence identity of 72% for their TM domains, while NET and SERT are 54% identical.

The main difference defining the selectivity of AoIA toward NET over DAT and SERT is confined to EL4a, which forms a structural clamp with TM10 and EL6 to stabilize AoIA toward the outer end of the vestibule (Fig. 5). Interactions are not very strong, as direct hydrogen bonds, salt bridges and hydrophobic interactions are limited. The essential difference is the loop connecting EL4a with EL4b, in particular A384 in NET, which is replaced by a proline in DAT and SERT (P387 and P403, respectively). Proline is a larger amino acid, whose structure also restricts the backbone conformation. The loop connecting EL4a with EL4b containing a proline, therefore, extends ~2–3 Å farther into the open vestibule and would overlap with M7^AoIA^ and C8^AoIA^. In parallel, T544^EL6^ of NET is smaller than H547 in DAT or R564 in SERT. This structural difference implies that AoIA perfectly fits into the outer vestibule of NET, in which AoIA acts as a wedge between the EL4a–EL4b loop and EL6. The larger amino acids in DAT and SERT on EL4 and EL6, along with the difference in the EL4a–EL4b loop conformation, likely prevent binding of AoIA in the same pose and/or sufficient occlusion of DAT and SERT. In this manner, AoIA may not interact with the salt-bridge-forming residues (R85 and D476 in DAT; R104 and E493 in SERT) or form a bridge between K6^AoIA^ and the aspartate in the S1 (D79 in DAT; D98 in SERT). In addition, key residue differences include the outer gate salt bridge, which is an aspartate in NET (D473) and DAT (D476) but one methylene moiety longer in SERT (E493). This difference not only affects the length of the salt bridge, but also repositions the carboxylate of E493 in SERT (Fig. 5d). In NET, D473 is a key residue for fixing the position of AoIA in its bound conformation. In addition, the side chains of K490 and E494 in SERT (T470 and T474 in NET) would overlap with AoIA and exert destabilizing electrostatic repulsions.

### AoIA shows antinociceptive efficacy in inflammatory pain model without sedation after subcutaneous administration in male mice

**Fig. 6:**
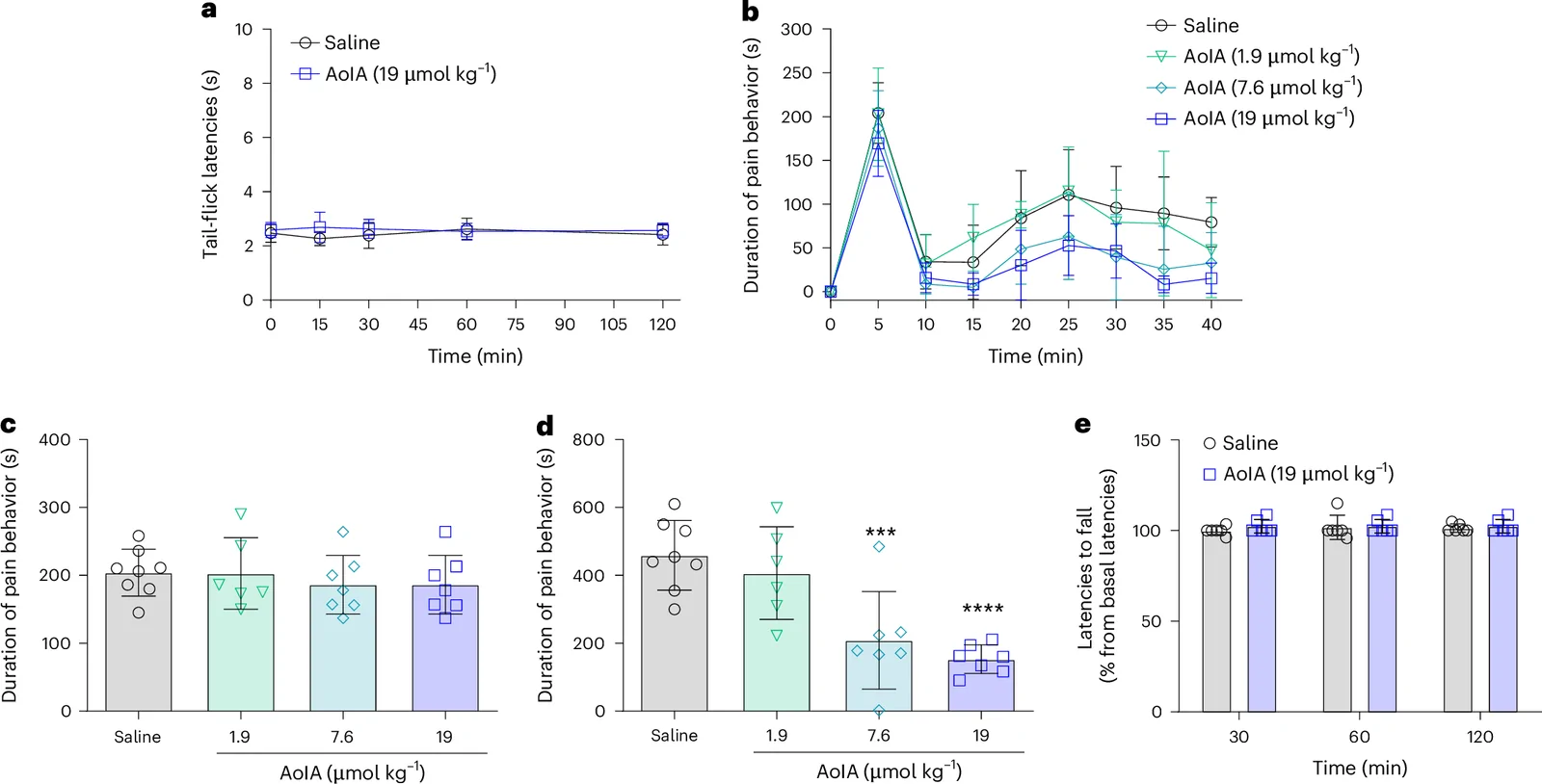
In vivo pharmacology of AoIA after s.c. administration in male mice. **a**, Radiant heat tail-flick test for acute nociceptive pain after administering saline (*n* = 6 mice) or AoIA (19 µmol kg^−1^, *n* = 6). Statistical analysis was conducted using a two-way ANOVA, *F*_(4, 50)_ = 0.1246, *P* = 0.9729. **b**, Formalin test for inflammatory pain and a dose-dependent and time-dependent effect after administering saline (*n* = 8) or AoIA (1.9 µmol kg^−1^, *n* = 6; 7.6 µmol kg^−1^, *n* = 7; 19 µmol kg^−1^, *n* = 7). **c**, Formalin test of nociceptive phase I after administering saline (*n* = 8) or AoIA (1.9 µmol kg^−1^, *n* = 6; 7.6 µmol kg^−1^, *n* = 7; 19 µmol kg^−1^, *n* = 7). Statistical analysis was conducted using a one-way ANOVA, *F*_(3, 24)_ = 0.3652, *P* = 0.7787. **d**, Formalin test of inflammatory phase II after administering saline (*n* = 8) and AoIA (1.9 µmol kg^−1^, *n* = 6; 7.6 µmol kg^−1^, *n* = 7; 19 µmol kg^−1^, *n* = 7). Statistical analysis was conducted using a one-way ANOVA, *F*_(3, 24)_ = 12.70, *P* < 0.0001. **e**, Rotarod test for sedation and motor coordination after administering saline (baseline: 285 ± 26 s, *n* = 6) or AoIA (19 µmol kg^−1^, baseline: 293 ± 11 s, *n* = 6). Statistical analysis was conducted using a two-way ANOVA, *F*_(1, 30)_ = 0.9776, *P* = 0.3307. The two-way ANOVAs in **a** and **e** were corrected with Bonferroni’s post hoc test. The one-way ANOVAs in **c** and **d** were corrected with Dunnett’s post hoc test. ***P < 0.001 and ****P < 0.0001 for drug versus saline groups. All data represent the mean ± s.d. Source data

Given the remarkable in vitro profile of AoIA, we were interested in evaluating the in vivo activity of AoIA after subcutaneous (s.c.) administration in male mice. First, behavioral properties of AoIA were assessed in a mouse model of acute nociceptive pain using the radiant heat tail-flick test. As shown in Fig. 6a, AoIA did not significantly increase tail-flick latencies compared to saline mice at any time point (two-way analysis of variance (ANOVA), *P* < 0.05), when administered s.c. at a dose of 19 µmol kg^−1^. In the same pain assay, clinically used analgesic drugs, including opioids (that is morphine, oxycodone, oxymorphone, buprenorphine and fentanyl), are well known to produce antinociceptive effects following systemic administration to rodents^61–64^.

**Extended Data Fig. 5:**
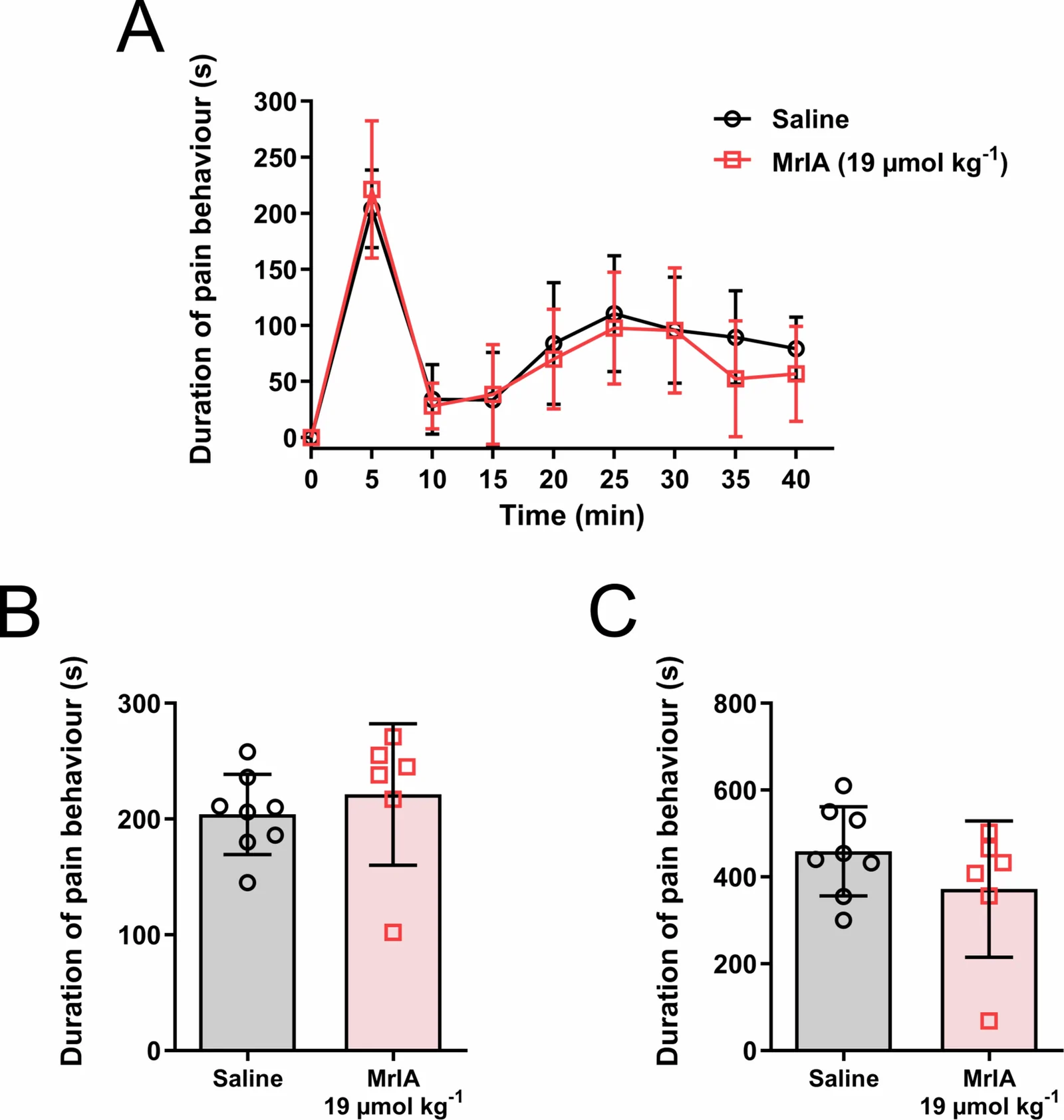
Effect of MrIA in the formalin test after s.c. administration in male mice. (**a**) Formalin test for inflammatory pain, dose and time-dependent effect; groups: saline (*n* = 6 mice) and MrIA (19 µmol kg^−1^, *n* = 6 mice); Two-way ANOVA with Bonferroni’s *post-hoc* test, F_(8, 108)_ = 1.070, *P* = 0.3033. (**b**) Formalin test, nociceptive Phase I; groups: saline (*n* = 8 mice) and MrIA (19 µmol kg^−1^, *n* = 6 mice); unpaired one-tailed t-test, *P* = 0.2561. (**c**) Formalin test, inflammatory Phase II; groups: saline (*n* = 8 mice) and MrIA (19 µmol kg^−1^, *n* = 6 mice); unpaired one-tailed t-test, *P* = 0.1167. All data represent means ± SD. Source data

To further investigate the therapeutic antinociceptive potential of AoIA, we examined its in vivo action in a model of inflammatory pain using the formalin test. Pain behavior was assessed in male mice that received intraplantar injection of formalin solution at the right hindpaw. Administration of AoIA produced time-dependent and dose-dependent reductions in pain behavior in formalin-injected mice, determined as the amount of time each animal spent licking, biting, lifting and flinching the formalin-injected paw (Fig. 6b). Whereas AoIA did not alter the pain response of formalin-injected male mice at any tested dose during nociceptive phase I (Fig. 6c), we measured an antinociceptive effect during inflammatory phase II of the formalin test (Fig. 6d). Significant effects were observed for AoIA at doses of 7.6 and 19 µmol kg^−1^, with reductions in pain behavior by 55% ± 31% and 67 ± 39%, respectively, compared to that seen in saline-treated mice. The calculated median antinociceptive effective dose (ED_50_) of AoIA in inflammatory phase II of the formalin test was 8.24 µmol kg^−1^ (95% confidence interval (CI): 3.06–22.2). We previously reported on the antinociceptive efficacy of the gold-standard analgesic morphine in the formalin test in mice after s.c. administration, with morphine having an ED_50_ value of 6.44 µmol kg^−1^ (ref. ^65^). Notably, the peptide MrIA given s.c. to male mice at an equimolar dose of 19 µmol kg^−1^ was devoid of an antinociceptive effect in the formalin test (two-way ANOVA, *P* < 0.05) (Extended Data Fig. 5).

To further address the in vivo properties of AoIA, we investigated its effects on motor coordination and its potential to induce sedation after s.c. administration in male mice using the rotarod test (Fig. 6e). Male mice were administered the highest antinociceptive effective dose in the formalin test with 19 µmol kg^−1^ AoIA, and latencies to fall off the rotarod were measured. The motor performance of animals treated with AoIA and saline was comparable over the entire observation period.

Our results, therefore, demonstrate that AoIA produces significant and potent antinociception in mice with inflammatory pain, without the liability of sedation or motor dysfunction after s.c. administration in male mice.

## Discussion

Our present study describes the pharmacological characterization of AoIA, a peptide originally isolated from the venom of *C*. *araneosus*. The cysteine framework of a conotoxin is defined by the arrangement of cysteine residues in its primary sequence, which can be a useful predictor of biological function^66^. AoIA displays the cysteine framework X (CC–CX(O/P)C), which is similar to framework I, as reflected in our proposed name for this conotoxin, following the established nomenclature^11,12^. Another defining feature of a conotoxin is its disulfide configuration. All identified χ-conotoxins, including AoIA, exhibit the ribbon configuration in their native state. This suggests that the ribbon configuration might represent a common feature for conotoxins belonging to the χ-pharmacological family. Moreover, the sequence GYKL contained within the intercysteine hairpin loop was previously identified as a pharmacophore region of MrIA^26,27^. This sequence is strongly conserved in AoIA and other χ-conotoxins (Fig. 1a). Indeed, conservative substitutions in the loop region in PnID and in synthetic analogs of MrIA were shown to maintain activity at NET^26,27,32^. Taken together, the data indicate that other known conotoxin sequences can be identified as potential NET inhibitors on the basis of structural similarities (Extended Data Table 3). Recently, it was postulated that the χ-conotoxins evolved with molluscivory in cone snails^33^. In fact, *C*. *marmoreus*, *C*. *pennaceus*, *C*. *aulicus* and *C*. *araneosus* are all molluscivorous cone snails. Hence, our data also support the expansion of peptides with potential selective NET inhibitory activity by specifically focusing on molluscivorous species.

Synthesis of AoIA and its isomer AoIA^*glob*^ enabled us to study differences between the two disulfide configurations in relating their structure and pharmacology. The β-hairpin structure of AoIA is also present in MrIA and PnID, but not in their globular isomers^32,67^. AoIA is over 20-fold more potent in inhibiting NET than AoIA^*glob*^. This confirms that the ribbon configuration is essential for the biological activity of χ-conotoxins. In the case of PnID, the ribbon isomer also exhibited the most significant inhibitory activity compared to the globular and bead isomers, with differences greater than tenfold^32^. It is worth nothing that AoIA exhibits around tenfold increased potency compared to MrIA in our NET assays, which could indicate that it may be a suitable candidate for further medically relevant development and applications. The remarkable selectivity displayed by AoIA for NET is a unique quality the χ-conotoxins possess as NET inhibitors, as most small-molecule NET inhibitors also display some affinity for SERT, DAT or both^68^. Furthermore, AoIA displays a mixed competitive/noncompetitive mode of inhibition based on its ability to reduce the maximum rate of NET uptake (*V*_max_) and to affect its substrate affinity (*K*_m_) in a concentration-dependent manner. MrIA has been previously described as an allosteric inhibitor of NET^20,34,57,69^; however, we suspect that MrIA follows the mixed kinetic model described in the current work. In fact, both S2 and S1 can be viewed as orthosteric sites for substrate binding by MATs. Because of the complexity of the transport cycle, compounds that bind to these sites can give rise to different modes of inhibition, which range from uncompetitive and noncompetitive to mixed competitive^70–72^. Thus, in this context, the term allostery captures digression from competitive inhibition but does not necessarily imply a binding site distinct from the substrate-binding sites.

Over the past year, cryo-EM structures of human NET have been reported by different groups^34,54,55,73^. Among them is a structure of NET in complex with *χ*-MrIA^EM^, a synthetic analog of MrIA^34^. The peptide *χ*-MrIA^EM^ was found to bind to a vestibular (allosteric) site of the transporter^34^. In the present work, we identify a binding mode for AoIA, which also involves the outer vestibule of NET, and we ascertained that AoIA reaches into the S1 binding site to overlap with the amine moiety of NE. This is, for the most part, identical to the binding mode elucidated from the structure of the NET:*χ*-MrIA^EM^ complex. Because of this partial orthosteric binding, however, AoIA and other *χ-*conotoxins cannot be classified as allosteric inhibitors of NET. Indeed, the entire binding site for AoIA in NET is distinct from yet partially overlapping the S1 site. This is in agreement with the earliest binding model proposed for MrIA, which was based on the bacterial leucine transporter^57^.

The current structure allows for the identification of amino acids essential for peptide–protein interactions. The chemical properties of the side chains of Y5^AoIA^ and K6^AoIA^ strategically complement the microenvironment in the S1 in a manner similar to cognate monoamine substrates, thus supporting the high affinity. Results from our structural analyses and mutagenesis studies highlight the importance of the size of the residues surrounding the vestibule in determining the binding of AoIA in NET, while also providing an explanation for the selectivity of AoIA for NET. In DAT and SERT, residues corresponding to T544^EL6^ and A389^EL4a^ are the bulkier residues histidine, arginine and proline, which preclude the entry of the peptide to the outer vestibule. Substituting L469^TM10^ to the corresponding phenylalanine in DAT also results in a steric overlap with M7^AoIA^, which is reflected in the decrease in AoIA potency in the L469F NET mutant. Our results agree with previous studies where E382^EL4^ and L469^TM10^ were both found to be essential for the binding of MrIA and MrIA^EM^ to NET, while substitutions of T474^TM10^ had no effect^34,57^. In a separate study, E382^EL4^ was also identified as a residue that interacts with a NE molecule in a secondary binding site (S2), further highlighting its role in forming S2 at NET^73^. A similar S2 located in the extracellular vestibule has also been found in SERT^71,74^.

**Extended Data Fig. 6:**
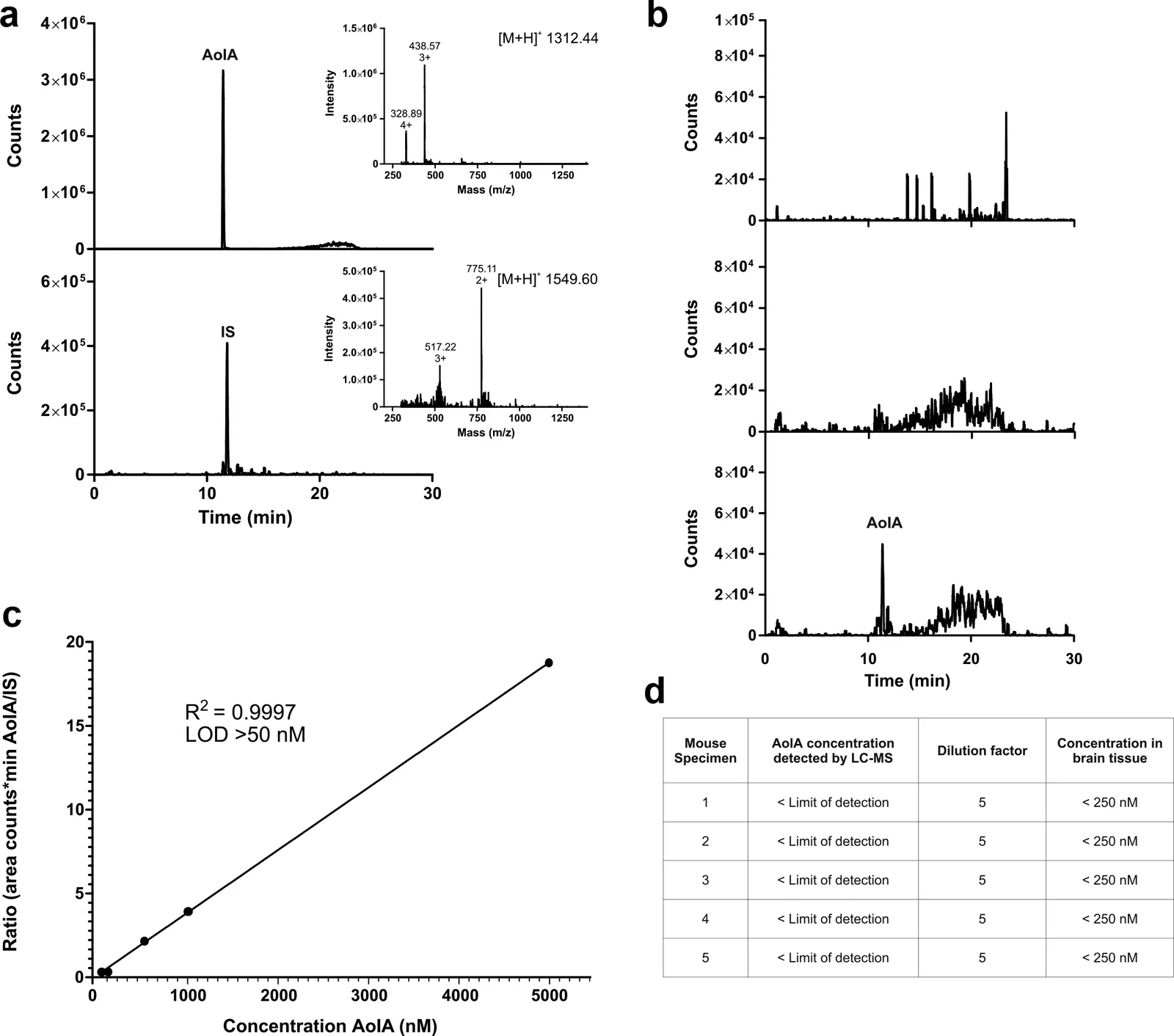
Bioanalytical analysis and absolute quantitation of peptide AoIA in brain tissue homogenates. (**a**) Ion chromatograms and mass spectra for the most abundant ion signal of AoIA (*t*_R_ = 11.4 min, ([M + 3H]^3+^)) as well as of the internal standard (IS) (*t*_R_ = 11.8 min, ([M + 2H]^2+^)) from samples in buffer are shown. (**b**) Extracted ion chromatograms (EIC) tracking the mass signal windows the IS (top chromatogram) and AoIA ([M + 3H]^3+^) (middle chromatogram) in blank mouse brain homogenates are provided, conforming, detection specificity (no other signal co-elutes at the same time) and the absence of interferes for the quantitation. Generated EIC trace for analyte spiked sample, 50 nM AoIA were added to brain homogenate and worked-up as described in the Methods section (bottom chromatogram). (**c**) Standard curve of AoIA in spiked rat brain homogenates with IS corrected quantitation. The concentrations 10, 50, 100, 500, 1000 and 5000 nM were measured. At 10 nM mass signal of the analyte was below limit of detection, therefore 50 nM was defined as lowest concentration for quantitation. The internal calibration graph indicates that 50 – 5000 nM were within the linear range (R^2^ = 0.999). (*t*_R_ = Retention time). (d) Result table of measurement outcomes. AoIA was not detectable above the limit of quantitation (50 nM in 5-fold diluted brain homogenates).

**Extended Data Fig. 7:**
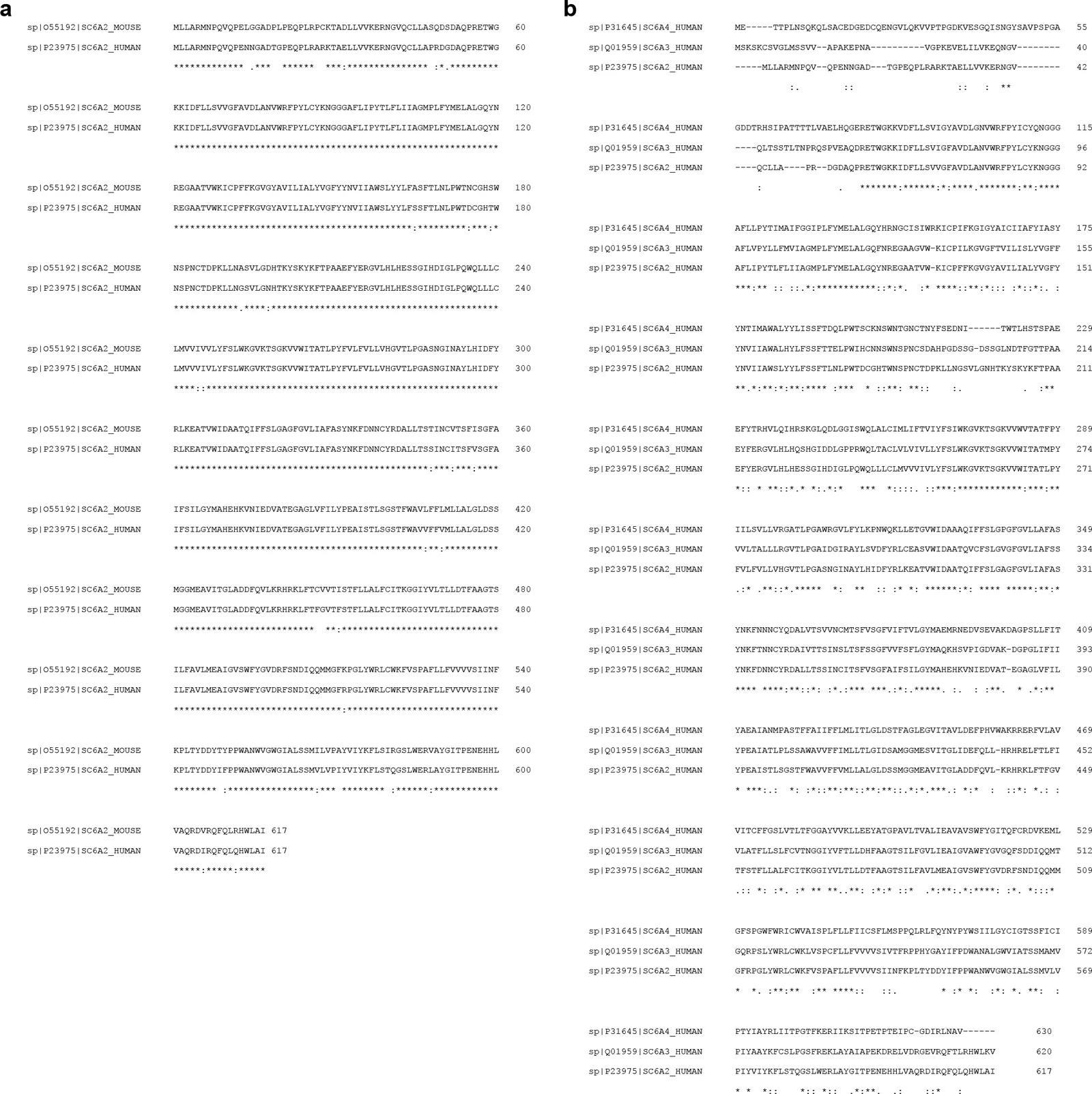
Sequence alignment of human NET (ID: P23975) with (**a**) mouse NET (ID: O55192) shows 94.3% identity, and (**b**) with human DAT (ID: Q01959, 67.5% identity) and SERT (ID: P31645, 49.7% identity) obtained from UniProt.org.^130^.

We have demonstrated in a mouse model of inflammatory pain that AoIA produces an analgesic effect in vivo (Fig. 6b–d). Mouse and human NET have a 94% sequence identity (Extended Data Fig. 7), which makes it a suitable model for translating our in vitro results. The lack of activity on other common targets involved in nociception, such as opioid receptors, TRP channels and voltage-gated sodium and calcium channels (Fig. 3), suggests that NET inhibition is its primary mechanism for analgesia. Indeed, it has been previously suggested for MrIA and Xen2174 that the analgesic effect of χ-conotoxins proceeds through adrenergic mechanisms in rodent models of pain^28–30^. Coadministration of Xen2174 with yohimbine, an α_2_-AR antagonist, abolished its analgesic effects in rats, thus indicating that its antinociceptive activity is NE-mediated^28^. Conotoxins can be developed into nonopioid analgesics, as demonstrated by the intrathecally applied ω-conotoxin, ziconotide (approved by the US Food and Drug Administration), which targets the N-type calcium channel^5^. Recent efforts also made use of cone snail-derived somatostatin analogs, which are able to produce antinociceptive effects in mice when administered intraperitoneally, suggesting that its site of action is outside the central nervous system^75^. The s.c. administration of AoIA to mice indicates that χ-conotoxins may also be capable of producing a systemic effect. Meanwhile, the peptide was undetectable in mouse brain tissues in our postmortem analyses (Extended Data Fig. 6), which, together with the sensitivity of the method, might argue against a central site of action.

Our behavioral studies in mice presented three pieces of data that could help to elucidate the mechanisms underlying the analgesic effect induced by AoIA: (1) it did not reduce the spinal nociceptive reflex in the radiant heat tail-flick test (Fig. 6a); (2) it did not alter the pain behavior in the nociceptive phase I of the formalin test (Fig. 6c); and (3) it showed efficacy in the inflammatory phase II of the formalin test (Fig. 6d). Although the heat stimulus activates peripheral thermal nociceptors, the behavioral readout (withdrawal of the tail) includes spinal reflex circuitry and is strongly modulated by spinal and supraspinal analgesic pathways. Centrally acting analgesics, such as opioids, reliably increase tail-flick latency^76^; however, this was not observed for our peptide. The presence of a spinal reflex would also indicate that detection of noxious stimuli by peripheral nociceptors is sustained and, therefore, not prevented by AoIA. We have established that AoIA, as a χ-conotoxin, is a selective NET inhibitor. NET inhibition is known to enhance descending noradrenergic inhibitory pathways, which are preferentially engaged during persistent, inflammatory or sensitization-dependent nociception, rather than during acute spinal reflexes^41,42,77,78^. This could also explain why AoIA did not cause a significant change in pain response in the acute, nociceptive phase I of the formalin test. In contrast, it significantly reduced the pain response in phase II, which involves peripheral inflammatory processes, sustained nociceptor activation and central sensitization, all of which are highly sensitive to modulation by enhanced noradrenergic signaling. Therefore, our results suggest that NET-mediated analgesic mechanisms in the periphery are not strongly recruited in acute responses to noxious stimuli, but more relevant in inflammatory pain states.

Previous animal studies performed on MrIA and Xen2174 delivered the χ-conotoxins intrathecally^28^. Indeed, conotoxins normally rely on an intrathecal route of administration to produce analgesia because of their poor ability to penetrate the blood–brain barrier^1,2^. As MrIA and its analogs have not been found to act on other ion channels and receptors that are common targets for analgesia^21,79^, the peripheral action of χ-conotoxins has so far not been elucidated. Most of the evidence for analgesic activity of NET inhibitors involves central processing in the locus coeruleus and the spinal cord^41,42^. The exact role of peripheral NET modulation in pain perception is still unclear. NET and α_2_-AR are also expressed in peripheral nerve endings in tissues^80–82^; therefore, it is also likely that AoIA could elicit its effect through prolonged adrenergic activation in the periphery. This has also been shown to work directly by peripherally active agonists of α_2_-AR (for example, xylazine^83,84^ or clonidine^85^ and analogs thereof such as medetomidine^86^). Neuroinflammation appears to be another component of the mechanism by which antidepressants, including NET inhibitors, could have a role in alleviating neuropathic pain conditions^87^. It has been shown that NE activates α_2_-ARs expressed in immune cells, which results in the release of β-endorphin and analgesia through peripheral activation of opioid receptors^88^. NE has also been shown to produce peripheral antinociception through activation of the α_2_-AR^89^. There are two main downstream antinociceptive cascades. First, the Gα_i_ inhibition of cAMP reduces protein kinase A activity, as well as protein kinase C through Epac1. These are the key protein kinases for sensitization of ion channels (for example, TRPV1)^90^. Second, the Gβγ complex can also exert antinociceptive effects by modulating ion channel activity, in particular through voltage-gated calcium channels limiting calcium entry^91^, as well as through voltage-gated potassium channels, particularly K_V_7 subtypes, hyperpolarizing the neurons^92^.

While the exact peripheral targets of AoIA remain to be fully elucidated, our findings open avenues for developing nonopioid analgesics targeting NET, with potential applications in neuropathic pain and other conditions linked to dysregulated adrenergic signaling. Future studies could explore AoIA’s efficacy in neuropathic pain models and investigate its long-term safety and therapeutic potential, particularly in comparison to existing NET inhibitors.

## Methods

### Peptide synthesis

#### Nonselective oxidative synthesis of AoIA globular and ribbon isomers

Microwave-assisted automated synthesis was carried out using a Liberty Blue peptide synthesizer. Preloaded Fmoc-Cys(Trt)-Wang resin (scale: 0.10 mmol) was swollen first in a small amount of DMF for 30 min. The resin was subjected to cycles of deprotection using 20% (v/v) 4-methylpiperidine in DMF, coupling using Fmoc-Xaa-OH/Oxyma/DIC at 2.5:2.5:2.5 equivalents relative to resin loading for 5 min and washing with DMF in between steps. A double coupling step was performed for hydrophobic amino acids and Fmoc-Arg(Pbf)-OH to ensure complete coupling. The reaction cycles were repeated until the complete linear sequence was assembled.

The peptide-containing resin was lyophilized overnight before treatment with TFA:TIPS:DODT:H_2_O (92.5%, 2.5%, 2.5% and 2.5% v/v) for 3.5 h. The mixture was filtered; then, the filtrate was collected over ice-cold *tert-*butyl methyl ether and refrigerated for 3 h for complete precipitation. The suspended peptide was collected by centrifugation and washed with DCM before dissolving in 5% acetonitrile in H_2_O. The peptide solution was lyophilized overnight to obtain a dry, white powder. After drying, oxidation was performed on the linear AoIA, as previously described with slight modifications^93^. The dry peptide sample was dissolved in an oxidative folding solution (2.5 mg ml^−1^) containing 30% DMSO in 0.1 M ammonium acetate (pH 6) and stirred at room temperature for 24 h. The mixture was quenched by adding H_2_O until a final concentration of 5% DMSO and lyophilized overnight. Two major products were expected in the oxidation: AoIA, the ribbon isomer, and AoIA^*glob*^, the globular isomer.

Analytical reverse-phase HPLC was performed using a Shimadzu Prominence HPLC system in an Inertsil ODS3 C_18_ column (5 µm, 4.6 × 250 mm) at a flow rate of 1.0 ml min^−1^. Mobile phases composed of 0.1% TFA in H_2_O (solvent A) and 0.1% TFA in acetonitrile (solvent B) were used for a linear gradient from 20% to 30% B for 15 min. Chromatograms were acquired by detecting absorbance at 220 nm and were analyzed using Shimadzu LabSolutions software. Semipreparative reverse-phase HPLC was used to obtain pure forms of both linear peptide and oxidized isomers in an Inertsil ODS3 C_18_ column (5 µm, 10 × 250 mm) at a flow rate of 3.0 ml min^−1^, using the same solvents and gradient. Fractions were collected and lyophilized until dry. Matrix-assisted laser desorption/ionization time-of-flight mass spectrometry (MS; Shimadzu Axima Confidence) was used to determine the [M + H]^+^ values of the products, using α-cyano-4-hydroxycinnamic acid as a matrix.

#### Selective synthesis of AoIA ribbon isomer

The ribbon isomer of AoIA was synthesized using automated Fmoc-tBu solid-phase peptide synthesis on a CSBio CS336X synthesizer (CSBio). Peptides were assembled on 2-chlorotritylchloride resin (substitution value: 1 mmol g^−1^, synthesis scale: 0.25 mmol). The first amino acid was coupled to the resin for 1 h in DCM, with a twofold excess of amino acid and eightfold excess of DIPEA. After coupling, the resin was treated three times with a mixture of methanol, DCM and DIPEA (17:2:1) for 1 min each to cap any remaining reactive functionalities on the resin. Fmoc deprotection was carried out with 20% (v/v) piperidine in DMF twice for 2 min. Subsequent amino acids were coupled for 30 min using a Fmoc-Xaa-OH/Oxyma/DIC equivalents of 4:4:8 relative to resin loading. DMF was used for resin washing between all steps. After chain assembly was completed, the resin was washed with DCM and dried under nitrogen.

For concomitant cleavage of the peptide chain and side-chain removal, the dry resin was then treated with TFA, TIPS and H_2_O (90:5:5, v/v/v) for 2 h, excess TFA was removed and the peptides were precipitated in ice-cold diethyl ether. The precipitate was filtered and dissolved in a 50:50 mixture of solvent A (0.05% TFA in water) and solvent B (90% acetonitrile, 0.05% TFA in water). The crude peptide was then lyophilized before purification by reverse-phase (RP)-HPLC.

Samples were purified using preparative and semipreparative RP-HPLC on a Shimadzu Prominence HPLC system with Phenomenex Gemini C_18_ columns (5 μm, 110 Å, 250 × 21.2 or 10 mm). Correct products were identified using electrospray ionization (ESI)-MS on a SCIEX API2000 MS instrument. Purity of all compounds (>95%) was assessed by analytical HPLC using a Vydac C_18_ column (150 × 2.1 mm) on a Shimadzu Prominence HPLC system using a linear gradient of 5–80% solvent B over 35 min.

First oxidation was achieved by dissolving peptides at a concentration of 1 mg ml^−1^ in 30% DMSO in 0.1 M ammonium acetate (pH 6). Oxidized peptide was purified after overnight incubation (>18 h at room temperature). After HPLC purification, the second oxidation was carried out by dissolving the peptide at a concentration of 1 mg ml^−1^ in 80% acetic acid followed by the addition of ten equivalents of iodine (dissolved in ethyl acetate). The reaction was monitored by ESI-MS and found to be complete within 10 min. The reaction was then quenched by addition of ascorbic acid until full decolorization and the peptide was purified as described above.

### NMR structure determination

NMR spectra for AoIA peak A and peak B were acquired using a Bruker Avance III HD 700-MHz spectrometer equipped with a cryoprobe. Roughly 1 mg of peptide was dissolved in 500 µl of 90% H_2_O and 10% D_2_O. The 2D spectra acquired included TOCSY, NOESY (mixing time 250 ms), ^13^C-HSQC and ^15^N-HSQC at 298 K. In addition, a series of TOCSY spectra were recorded from 288 K to 308 K at intervals of 5 K. All data were recorded and processed using Topspin 3.7.0 and referenced to DSS. Chemical shift assignments of the NMR spectra were performed using established sequential assignment methods^94^. Complete assignment of ^1^H resonances allowed assignments of ^13^C and ^15^N data recorded at natural abundance. In addition, ^13^C-HSQC was used to determine C_β_ and C_γ_ shifts of hydroxyproline, confirming a *trans* conformation in all cases.

Chemical shifts were used to predict backbone and side-chain dihedral angles using the programs TALOS-N^95^ and DISH^96^. Hydrogen-bond donors were identified by analysis of amide temperature coefficients. We first used CYANA (version 3.98.15)^97^ to perform automated NOE cross peak assignment and torsion angle dynamics on the peptides. The final set of NMR experimental restraints from these initial calculations were used as inputs for final structure calculation using cartesian dynamics and water refinement in CNS (version 1.2)^98^. Structural quality was assessed using MolProbity^99^. On the basis of these statistics, the 20 best models from a calculated set of 50 were chosen to represent the solution structures of the globular and (native) ribbon isomers of AoIA.

### Cell culture

HEK293 cells stably expressing the YFP-tagged and nontagged human isoforms of SERT (YhSERT), DAT (YhDAT) and NET (hNET) and GripTite HEK293 MSR cells (Thermo Fisher) were cultured in DMEM with high glucose (4.5 g L^−1^) and l-glutamine (584 mg L^−1^), supplemented with 10% fetal calf serum, 100 U per ml penicillin and 100 µg ml^−1^ streptomycin. The cells were cultured in a humidified atmosphere (37 °C, 5% CO_2_). Geneticin was added to maintain the selection pressure for the stable cell lines (50 µg ml^−1^ for YhSERT, YhDAT and hNET; 100 µg ml^−1^ for HEK293 MSR). Cells were typically seeded 24 h before the experiments. For uptake inhibition and saturation experiments, cells were seeded at 3.6 × 10^4^ cells in 0.2 ml into 96-well plates coated with poly(d-lysine).

### Site-directed mutagenesis and transfections

The NET mutants were generated in the YFP-hNET plasmid (constructed in the lab, as previously described)^100^ using the QuikChange Lightning site-directed mutagenesis kit (Agilent). The following primers were obtained from Microsynth: forward E382A, 5′-ggatgtggccacagcaggagctggcctag-3′; reverse E382A, 5′-ctaggccagctcctgctgtggccacatcc-3′; forward T544H, 5′-catcaacttcaagccactccactacgacgactacatcttc-3′; reverse T544H, 5′-gaagatgtagtcgtcgtagtggagtggcttgaagttgatg-3′; forward L469F, 5′-ccaagggtggaatttacgtcttcaccctcctggac-3′; reverse L469F, 5′-gtccaggagggtgaagacgtaaattccacccttgg-3′; forward T474H, 5′-ttgaccctcctggaccactttgctgcgggcac-3′; reverse T474H, 5′-gtgcccgcagcaaagtggtccaggagggtcaa-3′. All mutant plasmids were sequenced before use (LGC Genomics). Uptake inhibition experiments on YhNET mutants were performed in HEK293 MSR cells transiently transfected with polyethyleneimine (PEI). First, 800,000 cells were seeded into a 6-cm cell culture dish. The following day, 3 µg of DNA and 9 µg of PEI were diluted in 200 µl of serum-free DMEM. The mixture was vortexed for 10 s and incubated for 15 min at room temperature before adding to the cells. The assays were performed 48 h after the transfections.

### Radioligand-based assays

For uptake inhibition experiments, the cell medium was aspirated and the cells were washed once with 0.2 ml of KHB per well (120 mM NaCl, 3 mM KCl, 2 mM CaCl_2_, 2 mM MgCl_2_, 20 mM glucose and 10 mM HEPES NaOH, pH 7.3–7.4) at room temperature. The cells were preincubated with increasing concentrations of the compound of interest, diluted in KHB (0.05 ml per well). The preincubation solution was replaced with 0.05 ml of KHB solution per well still containing the substance of interest and the tritiated substrate (100 nM [^3^H]5HT for YhSERT; 100 nM [^3^H]DA or 50 nM [^3^H]MPP^+^ for YhDAT; 20 nM [^3^H]MPP^+^ for hNET) after 5 min (YhSERT and YhDAT) or 15 min (hNET). Substrate uptake was stopped after 1 min (for [^3^H]DA and [^3^H]5HT) or 5 min (for [^3^H]MPP^+^) through exchange of the substrate-containing buffer with 0.2 ml of ice-cold KHB. The KHB was aspirated immediately. Nonspecific uptake was determined in the presence of 3 µM paroxetine (for YhSERT) or 50 µM GBR12909 (for YhDAT and hNET). Each condition was performed in triplicate.

For uptake saturation experiments, hNET-expressing HEK293 cells were incubated with 50 µl of KHB containing 0.3–100 µM MPP^+^. The dilution row was prepared by combining solutions of 0.02 µM [^3^H]MPP^+^ with increasing concentrations of cold MPP^+^. Substrate-containing solutions were added to the cells and the uptake was terminated after 5 min by exchanging with ice-cold KHB. To determine the effect of the compound, the cells were preincubated with increasing concentrations of AoIA for 15 min before uptake measurements. Nonspecific uptake was determined in the presence of 50 µM GBR12909.

After each uptake inhibition and saturation experiment, cells were lysed and prepared for [^3^H] content analysis by adding 0.2 ml of Ultima Gold XR liquid scintillation cocktail. Measurements were performed in a 1450 MicroBeta microplate liquid scintillation counter. Uptake inhibition data were fitted using nonlinear regression models in GraphPad Prism (version 10.0), specifically inhibitor concentration versus normalized response with variable slope. Uptake saturation data were fitted to the Michaelis–Menten equation to determine kinetic parameters of uptake.

For radioligand binding competition assays, membranes were first prepared from HEK293 cells stably expressing human NET and mouse MOR, KOR and DOR, following previously published protocols^101^. Cells were harvested from dishes using a plastic scraper and ice-cold PBS. Cells were recovered as a pellet after centrifugation (400*g*) for 10 min at 4 °C and then resuspended in hypotonic HME buffer (2 mM MgCl_2_, 1 mM EDTA and 20 mM HEPES NaOH, pH 7.5). The cell suspension was frozen rapidly by immersing in liquid N_2_ and then immediately thawed and sonicated for three cycles of 12 pulses per minute. Membrane fractions were collected after centrifugation at 40,000*g* for 15 min and then resuspended in HME buffer. Protein concentration was determined using a Coomassie brilliant blue kit (Bio-Rad Laboratories). For NET, the binding reactions were carried out in a final volume of 0.25 ml of binding buffer (300 mM NaCl, 5 mM KCl and 50 mM Tris-HCl, pH 7.4) containing 15 µg of membranes, 1 nM [^3^H]-nisoxetine and increasing concentrations of test compound for 60 min at 20–22 °C. For MOR, KOR and DOR, assays were performed using binding buffer (50 mM Tris-HCl, 10 mM MgCl_2_ and 0.1% BSA, pH 7.4, supplemented with 75 µl of [^3^H]-diprenorphine (1 nM final concentration), test ligands (4× stock) and membranes (KOR: 7 µg per assay, MOR: 50 µg per assay, DOR: 25 µg per assay) and incubated at 37 °C for 1 h. Nonspecific binding was determined in the presence of 10 µM desipramine for NET or 10 µM naloxone for MOR, KOR and DOR. Following incubation, the reactions were terminated by rapid filtration through polyethylenimine-coated glass fiber filters using a Skatron cell harvester (Skatron) and cold washing buffer (120 mM NaCl, 1 mM MgCl_2_ and 10 mM Tris-HCl pH 7.4 or 50 mM Tris-HCl buffer pH 7.4). The amount of radioactivity in the filters was measured using a Tri-Carb 2800TR liquid scintillation analyzer (PerkinElmer). Data were normalized to the fraction of maximum bound radioligand: approximately 2,400–3,000 fmol mg^−1^ protein per assay for NET, 1,500 fmol mg^−1^ protein for DOR, 4,000–4,500 fmol mg^−1^ protein for KOR and 500–1,000 fmol mg^−1^ protein for MOR. The *K*_i_ was calculated from the measured IC_50_ using the Cheng–Prusoff equation^102^.

### Calcium microfluorimetry

#### Chemicals and solutions

Allyl isothiocyanate was obtained from Fisher Scientific, while capsaicin was obtained from Biotrend. All substances were diluted in extracellular solution, composed of the following ingredients: 145 mM NaCl, 5 mM KCl, 10 mM glucose, 10 mM HEPES, 1.25 mM CaCl_2_ and 1 mM MgCl_2_ buffered to pH 7.4 with NaOH. Salts and HEPES were obtained from Carl Roth, Sigma-Aldrich or Merck, while NaOH was obtained from Thermo Fisher Scientific.

#### Isolation of primary dorsal root ganglion neurons

Breeding, killing and animal handling procedures were performed according to regulations of animal care and welfare. Experiments were carried out in accordance with the European Communities Council Directive of 24 November 1986 (86/609/EEC). Wild-type C57BL/6 mice (males, 8–16 weeks old) were anaesthetized by exposure to isoflurane or rising CO_2_ levels and killed by cervical dislocation. DRGs from all spinal levels were excised and transferred to DMEM (D5648, Sigma-Aldrich) containing 1% streptomycin–penicillin and 1% L-glutamine (Lonza), treated with 1 mg ml^−1^ collagenase (Sigma-Aldrich) and 3 mg ml^−1^ dispase II (Roche Applied Science) for 55 min at 37 °C. Digested DRGs were then mechanically dissociated with a Pasteur pipette, centrifuged at 1,200 rpm for 5 min and plated in poly(D-lysine)-coated black-wall clear-bottom 96-well plates and incubated overnight at 37 °C and 5% CO_2_.

#### HEK293t cells and transient transfection

HEK293t cells were grown in DMEM (D5648, Sigma-Aldrich), supplemented with penicillin, streptomycin and L-glutamine (1% each, all from Lonza). HEK293t cells were transfected with human TRPA1 (hTRPA1) using jetPEI transfection reagent (Polyplus). The plasmid was obtained from P. Heppenstall and cloned into a pcDNA3.1 vector^103,104^. The cells were then spread on poly(D-lysine)-coated black-wall clear-bottom 96-well plates (~30,000 cells per well) and incubated overnight at 37 °C and 5% CO_2_.

#### Imaging

The microfluorimetry of cytosolic calcium levels was performed as described previously^105^. Briefly, cells were loaded with a Calcium 6 kit (Molecular Devices) in extracellular solution for 2 h according to the manufacturer’s protocol. Cells were not washed as fluorescence of the extracellular dye is absorbed by a dark dye in the kit. A pipetting fluorescence plate reader (FlexStation 3, Molecular Devices) was used to measure the change in intracellular Ca^2+^ concentration in hTRPA1-transfected, hTRPV1-transfected and nontransfected HEK293t cells. Calcium 6 fluorescence, which was excited every 2 s at 485 nm, served as an index of intracellular calcium levels. Using a preset protocol, 50 μl of the assay solution was automatically pipetted onto the cells in 100 μl of solution.

### Electrophysiology

Patch-clamp experiments on primary mouse dorsal root ganglion neurons isolated as described above were carried out in whole-cell voltage-clamp mode using a Multiclamp 700B amplifier and Axon Digidata 1550B data digitizer hardware operated by Clampex 11.3 software (Molecular Devices). Micropipettes were pulled in five steps using a P-97 Flaming/Brown micropipette puller (Sutter Instruments) from GB150F-8P borosilicate capillaries with filament (Science Products) and were fire-polished using an MF-830 microforge (Narishige Scientific Instrument Laboratory) resulting in 1.5–3.3 MΩ of pipette resistance in the bath solutions. Series resistance was corrected electronically by 75–85%. Pipettes were filled with internal solution consisting of 140 mM CsCl, 5 mM NaCl, 1 mM MgCl_2_, 10 mM HEPES and 5 mM EGTA, pH 7.3 (adjusted with CsOH). The bath solution consisted of 140 mM NaCl, 1 mM MgCl_2_, 1 mM CaCl_2_, 10 mM HEPES, 10 mM glucose, 0.1 mM CoCl_2_ and 10 mM TEA-Cl, pH 7.4 (adjusted with NaOH). All experiments were implemented at room temperature (~23 °C). For measurements, cells were held at hyperpolarized test potential (−100 mV) and depolarized to 0 mV for 40 ms. Optionally, to isolate slow-kinetics TTX-insensitive Na_V_ currents (mainly representing Na_V_1.8) from compound Na_V_ currents, a conditioning depolarizing prepulse to −50 mV for 500 ms was applied^52,53,106^. The interpulse interval was set to 10 s to provide full recovery of Na_V_ channels from inactivation. Data were sampled at 50 kHz and lowpass-filtered at 10 kHz. Continuous perfusion with bath solution and application of AoIA (10 µM) or suzetrigine (100 nM) was carried out using VC^3^ eight-channel software-controlled gravity-driven perfusion system (ALA Scientific Instruments). Electrophysiological recordings were analyzed using Clampfit 11.3 software (Molecular Devices).

### BRET assay

HEK293T cells were transfected with 1 µg of α_2A_-AR (in peGFP-N1 plasmid) and 1 µg of G-CASE G-protein biosensor plasmid, using jetPRIME transfection reagent (Polyplus). Cells were incubated with the transfection mixture for 6 h and subsequently transferred into white clear-bottom 96-well plates, at a cell density of 50,000 cells per well in 100 µl of DMEM, substituted with 10% FBS and 1% penicillin–streptomycin. Following overnight incubation, the cells were serum-starved for 1 h at 37 °C in DMEM without any additives. When measuring antagonism, the antagonists were added 30 min into the serum-starved cells, prepared in Hanks’ balanced salt solution (HBSS) (Gibco). The nanoluciferase substrates furimazine (Promega) and Hikarazine Z108 (Synthelis Biotech) were diluted 1:50 and 1:100, respectively, and ligands were prepared (4×) for agonist measurements and (5×) for antagonism in HBSS. Before starting to read, 50 µl of furimazine or Hikarazine was added to the cells and incubated for 5 min at 37 °C. Then, 5 min into the read, 50 µl of each ligand was added to each well. BRET was recorded for a total of 25 min at 37 °C at 460 nm (nanoluciferase) and 530 nm (cpVenus) using a FlexStation 3 (Molecular Devices). The data are presented as BRET ratios, calculated by dividing the emission at 530 nm by the emission at 460 nm.

### Cryo-electron microscopy

#### Expression, purification and nanodisc reconstitution of NET

The human wild-type NET (SLC6A2) was generated by integrating its coding sequence into the pEG BacMam vector with a 1× Flag tag positioned at the N terminus and connected by a GGS sequence. The construct was expressed in HEK293S GnTI^−^ cells, which were cultured in SMM-293TII medium (SinoBiological) supplemented with 2% FBS (Gibco). Following centrifugation (1,000*g*, 10 min, 4 °C), the cells were harvested and lysed using a Dounce homogenizer (Merck Millipore) in a buffer containing 50 mM HEPES (pH 7.5) and 150 mM NaCl, with protease inhibitors (aprotinin, leupeptin and pepstatin A; MedChemExpress) included to prevent degradation. The lysate was then subjected to centrifugation (8,000*g*, 25 min, 4 °C) to remove cell debris and the membranes were isolated by ultracentrifugation (100,000*g*, 1 h, 4 °C). The membrane fraction was solubilized in a buffer containing 50 mM HEPES (pH 7.5), 150 mM NaCl, 1% (w/v) dodecyl-β-D-maltoside (DDM; Anatrace) and 0.1% (w/v) cholesterol hemisuccinate (CHS, Anatrace) for 2.5 h at 4 °C. After centrifugation (30,000*g*, 1 h, 4 °C), the supernatant was incubated with anti-DYKDDDDK G1 affinity resin (GenScript) for 2 h at 4 °C. The bound protein was eluted using 0.2 mg ml^−1^ DYKDDDDK peptide (GenScript) and further purified by size-exclusion chromatography (SEC) on a Superose6 Increase 10/300 GL column (GE Healthcare) in SEC buffer (20 mM HEPES pH 7.5, 150 mM NaCl and 0.012% DDM). The peak fractions were concentrated to 2.0 mg ml^−1^ using a 100-kDa Amicon centrifugal filter (Merck Millipore).

For the reconstitution of NET into nanodiscs, brain polar lipids (Avanti Polar Lipids) were dissolved in chloroform, dried under argon and stored under vacuum overnight. The lipid film was rehydrated in 20 mM Tris pH 7.5, 100 mM NaCl and 0.1% DDM to a final concentration of 150 mM. NET, MSP1D1 (membrane scaffold protein) and lipids were mixed in a molar ratio of 1:2:20 in a buffer containing 20 mM HEPES pH 7.5,100 mM NaCl and 10 mM sodium cholate, followed by incubation for 2 h at 4 °C. Bio-Beads SM-2 resin (Bio-Rad) was added at a concentration of 320 mg ml^−1^ and stirred overnight at 4 °C to facilitate nanodisc formation. The sample was purified by SEC using a Superose6 Increase 10/300 GL column (GE Healthcare) in 20 mM HEPES (pH 7.5) and 150 mM NaCl. The peak fractions were collected and concentrated to 1.5 mg ml^−1^ and 3.0 mg ml^−1^ for cryo-EM analysis.

#### Grid preparation and data processing

For structural analysis of NET complexes with 10 μM AoIA, 2.3 μl of purified protein was applied to glow-discharged (SuPro Instruments) Quantifoil R1.2/1.3 Au 300-mesh grids. After blotting (Vitrobot Mark IV, Thermo Fisher, 8 °C, 100% humidity, 4.5 s, blot force: 1), the grids were plunge-frozen in liquid ethane. Data collection was performed on a 300-kV Titan Krios G4 (FEI) equipped with an E-CFEG and Falcon4i detector at the Shanghai Advanced EM Center. Imaging parameters included a pixel size of 0.73 Å and a total dose of 50 e^−^ per Å^2^ across 36 frames. Motion correction was performed using MotionCor2.

For the 3.0 mg ml^−1^ NET–AoIA dataset, 7,752 dose-weighted micrographs were processed in cryoSPARC version 4.3.1, with patch contrast transfer function (CTF) estimation parameters. Micrographs with CTF resolutions below 3.8 Å were excluded. Initial particle selection was performed using blob picker with a diameter of 140 Å. After iterative 2D classification,195,302 high-quality particles were retained for 3D refinement. After 12 rounds of multireference 3D classification and postprocessing, a 3.20-Å map was obtained; however, it exhibited preferred orientations. Therefore, we introduced a new 1.5 mg ml^−1^ dataset, focusing on collecting side-view particles. These side-view particles were used as seeds to enrich more side-view particles. After multiple rounds of iteration, we ultimately obtained a 2.77-Å map with improved preferred orientation.

The final structural models of NET were based on the previously determined structure (PDB 8Y95) and were fitted into the cryo-EM maps using UCSF ChimeraX. Manual refinements were performed in Coot and ligand constraints were generated using PHENIX1.17_elbow. The final model refinement was conducted with phenix.real_space_refine, resulting in high-resolution structures of NET in complex with various inhibitors.

### MD simulations

#### System building

Monomer A of the experimentally solved cryo-EM structure of AoIA-bound NET was used to build the simulation system. Initially, the membrane was assembled and equilibrated around NET. For this, the structure of NET was uploaded to the Position of Proteins in Membranes server to determine its position in the membrane^107^. The martinize2 pipeline was used to convert the transporter into a coarse-grained representation defined by the Martini 3 force field^108,109^. The insane.py script was used to insert the protein into an asymmetric membrane with the following composition (POPC:POPE:POPS:CHOL = 20:35:15:30 in the inner leaflet and POPC:POPE:CHOL = 60:10:30 in the outer leaflet)^110^. Water and salt were added to the system to create a concentration of 150 mM.

Coarse-grained MD simulations were carried out using GROMACS (version 2025.2)^111–113^. This procedure was performed four times to independently assemble four systems of membrane embedded NET. To equilibrate the membranes, a 1-µs MD simulation was conducted using a time step of 0.02 ps, applying position restraints of 1000 kJ mol^−1^ nm^−2^ only on the protein. The temperature was maintained at 310 K using the v-rescaling thermostat while a pressure of 1 bar was maintained using the c-rescaling barostat^114,115^. The reaction field algorithm was used for electrostatic interactions with a cutoff of 1.1 nm, while a single cutoff of 1.1 nm was used for van der Waals interactions.

The equilibrated systems were converted to an all-atom representation (backmapping) using the ezAlign software^116^. To avoid spurious structural imprecisions caused by conversion of the coarse-grained representation to all-atom representation, the backmapped protein was deleted and replaced with the original cryo-EM structure of of NET bound to the AoIA conotoxin. Additionally, for the NET–AoIA–NE systems, NE was added according to its location in the PDB 8ZPB structure^55^. The membed procedure was used to relieve possible atomic overlaps between the inserted protein and the equilibrated membrane as described in previous publications^117,118^, creating four systems for every assembly.

#### All-atom simulation

We used the charmm36-jul2022 force field to describe the system^119^. The topology parameters for NE were generated using the ‘ligand reader and modeler tool’ from CHARMM-GUI^120,121^. The assembled systems were energy-minimized and equilibrated in four steps of 2.5 ns each, while gradually releasing the position restraints applied on the Cα atoms and the atoms of the ligands using the following scheme (1,000, 100, 10 and 1 kJ mol^−1^ nm^−2^)^122,123^. To fully equilibrate the system, additional 50-ns simulation was carried out without position restraints. The production simulations were run for 0.5 µs per system. To maintain the temperature at 310 K, a v-rescale (*τ* = 0.5 ps) thermostat was used, while separately coupling protein, membrane and solvent. The pressure was maintained at 1 bar using the Parrinello–Rahman barostat in a semiisotropic manner and applying a coupling constant of 20.1 ps (ref. ^124^). The smooth particle mesh Ewald method was used to describe long-range electrostatic interactions applying a cutoff of 0.9 nm, while the van der Waals interactions were described using Lennard–Jones potentials with a cutoff of 0.9 nm (ref. ^125^).

#### Analysis

The trajectories were processed and analyzed using GROMACS packages (version 2025.2). For visualization, we used VMD (version 1.9.4) and PyMol (version 2.5)^126^.

### Animals and drug administration

Experiments were performed in male SWISS mice (RjOrl:SWISS; 6–8 weeks old, body weight: 30–35 g) purchased from Janvier Labs. All animal care and experimental procedures were in accordance with the ethical guidelines for the animal welfare standards of the European Communities Council Directive (2010/63/EU) and were approved by the Committee of Animal Care of the Austrian Federal Ministry of Science and Research. Here, we used male mice to establish pain and behavioral responses as these types of studies are commonly reported using male mice, female mice or both sexes. Male mice were group-housed (maximum five animals per cage) in a temperature-controlled (22–23 °C) and humidity-controlled (60–70%) specific-pathogen-free room with a 12-h light–dark cycle and with free access to food and water. All behavioral experiments were performed during the light cycle. Efforts were made to minimize animal suffering and to reduce the number of mice use. Animals were assigned to groups randomly and drug treatment experiments were conducted in a blinded fashion. Experimental drugs AoIA and MrlA were prepared in sterile physiological 0.9% saline solution. Test compounds or vehicle (saline) was s.c. administered in a volume of 10 µl g^−1^ body weight.

### In vivo pharmacology

#### Tail-flick test

The radiant heat tail-flick test was performed in male mice using an UB 37360 Ugo Basile analgesiometer (Ugo Basile) as described previously^127^. The reaction time required by the mouse to remove its tail after application of the radiant heat was measured and defined as the tail-flick latency (in seconds). Tail-flick latencies were measured before and after s.c. administration of saline (control) or AoIA (19 µmol kg^−1^) at different time points (that is, 15, 30, 60 and 120 min; test latency). A cutoff time of 10 s was used to minimize tissue damage.

#### Formalin test

The formalin-induced inflammatory pain was performed as described previously^65,127^. Following a habituation period of 15 min to individual transparent observation chambers, male mice were s.c. administered saline (control), AoIA (1.9, 7.6 and 19 µmol kg^−1^) or MrIA (19 µmol kg^−1^), 15 min before the injection of 20 µl of 5% formalin aqueous solution to the plantar surface of the right hindpaw. Each mouse was observed for 40 min in 5-min intervals following the injection of formalin. The amount of time (in seconds) each animal spent licking, biting, lifting and flinching the formalin-injected paw (pain behavior) was recorded during phase I (0–5 min) and phase II (15–40 min). Antinociceptive effect expressed as a percentage (%) was calculated according to the following formula: 100 × [(*C* – *T*)/*C*], where *C* is the mean time in the control (saline) group and *T* is the time in the drug-treated group. Dose–response relationships of percentage inhibition of pain behavior were constructed and the dose necessary to produce a 50% effect (ED_50_) and 95% CIs was calculated according to the method of Litchfield and Wilcoxon^128^.

#### Rotarod test

The rotarod test was performed using the accelerating rotarod treadmill (Acceler Rota-Rod 7650, Ugo Basile) for mice (diameter: 3.5 cm) as described previously^127^. Male mice were habituated to the equipment in two training sessions (30 min apart) 1 day before testing. On the experimental day, mice were placed on the rotarod and the treadmill was accelerated from 4 to 40 rpm over a period of 5 min. The time spent on the drum was recorded for each mouse before and at 30, 60 and 120 min after s.c. administration of saline or test peptide. Decreased latencies to fall in the rotarod test indicate impaired motor performance. A 300-s cutoff time was used. Percentage changes from the rotarod latencies obtained before (baseline, *B*) and after drug administration (test, *T*) were calculated as 100 × (*T*/*B*).

### Sample preparation for bioanalysis

First, 2 h after the s.c. administration of 19 µmol kg^−1^ AoIA, the mice were killed and the brains were removed, washed with sterile physiological 0.9% saline and frozen in dry ice until use. Mouse brain tissues were homogenized in PBS (1:4, w/w) in 2-ml tubes with beads at 5,500 rpm for five cycles of 10 s with a 10-s break using the Precellys Evolution Touch homogenizer (Bertin Technologies). To 50 µl of samples, 30 µl of 8 M urea solution was added and incubated on ice for 10 min. Afterward, 20 µl of 40% (w/v) trichloroacetic acid (containing internal standard, 200 nM final concentration) was added for protein precipitation, incubated on ice for 10 min and centrifuged at 16,000 rpm at 4 °C for 15 min. Supernatant was transferred into an HPLC vial for LC–MS analysis.

### Quantification of peptide in biological matrix using MS

A Vanquish LC–linear quadrupole MS (ISQ-EM) system (Thermo Scientific) was used for sample analysis. Chromatographic separation was performed with a Phenomenex Jupiter C_18_ modified silica column (150 × 1.0 mm inner diameter, 4 µm) at 55 °C, using 0.1% formic acid in ddH_2_O as mobile phase A and acetonitrile, ddH_2_O and formic acid (80%, 20%, 0.1% v/v/v) as mobile phase B with a flow rate of 0.3 ml min^−1^. For the separation conditions, an initial hold at 0% buffer B for 7 min, a separation gradient from 0% to 99% B within 10 min and a 3-min wash at 99% B, followed by a 9-min equilibration phase at initial conditions, were performed. MS was performed in positive ionization mode using ambient electrospray ionization and a mass range *m*/*z* 300–1,400. A heated ESI source was operated with the following conditions: vaporizer temperature, 200 °C; ion transfer tube temperature, 300 °C; source voltage positive ions, 3,000 V; sheath gas pressure, 2.999 bar; auxiliary gas pressure, 0.503 bar; sweep gas pressure, 0.131 bar. For quantification, the most abundant ions were used for AoIA (*t*_R_ = 11.4 min, *m*/*z* 438.15, [M + 3H]^3+^), IS *(t*_R_ = 11.8 min, *m*/*z* 775.31 [M + 2H]^2+^). The area under the curve was determined and the ratio between AoIA and IS was calculated for the quantitation of analytes using a linear regression model. The software used for quantification was Chromeleon (version 7.3).

### Statistical and data analysis

Data treatment and statistical analysis were performed using GraphPad Prism (versions 5.04, 9.4 and 10.4.2). In vivo data were statistically evaluated using a one-way ANOVA with Dunnett’s multiple-comparison post hoc test, two-way ANOVA with Bonferroni’s post hoc test or unpaired *t*-test, with significance set at *P* < 0.05. Experimental data are presented as the mean ± s.d., with *n* representing the number of biological replicates.

### Reporting summary

Further information on research design is available in the Nature Portfolio Reporting Summary linked to this article.

## Online content

Any methods, additional references, Nature Portfolio reporting summaries, source data, extended data, supplementary information, acknowledgements, peer review information; details of author contributions and competing interests; and statements of data and code availability are available at https://doi.org/10.1038/s41594-026-01838-z.

## Supplementary information

Supplementary Information Supplementary Table 1 and Fig. 1.

Reporting Summary

Peer Review File

## Source data

Source Data Fig. 1 Statistical source data.

Source Data Fig. 2 Statistical source data.

Source Data Fig. 3 Statistical source data.

Source Data Fig. 4 Statistical source data.

Source Data Fig. 6 Statistical source data.

Source Data Extended Data Fig. 2 Statistical source data.

Source Data Extended Data Fig. 3 Statistical source data.

Source Data Extended Data Fig. 5 Statistical source data.

## Data Availability

Peptide structures and chemical shifts were submitted to the Protein Data Bank and Biological Magnetic Resonance Bank under the following accession codes: globular isomer AoIA, PDB 9PBO and BMRB 31256; native ribbon isomer AoIA, PDB 9PAZ and BMRB 31255. Protein structural data for hNET:χ-AoIA were deposited to the EM Data Bank and the Protein Data Bank under accession codes EMD-62139 and PDB 9K6X. Additional data are accessible via Zenodo at https://doi.org/10.5281/zenodo.19855010 (ref. ^129^). MD data were also deposited to Zenodo at https://doi.org/10.5281/zenodo.18715044 (ref. ^131^). Other data supporting the findings of this study are available from the corresponding authors upon request. Source data are provided with this paper.

## Extended data

**Extended Data Table 1:** NMR Summary statistics for globular and ribbon isomers of AoIA

	Peak A (globular, AoIA^*glob*^)	Peak B (ribbon, AoIA)
**Energies**		
Overall (kcal/mol)	-289.10 ± 17.52	-306.63 ± 15.81
Bonds (kcal/mol)	7.12 ± 0.73	5.52 ± 0.68
Angles (kcal/mol)	25.37 ± 2.78	19.03 ± 3.86
Improper (kcal/mol)	5.51 ± 0.94	6.15 ± 2.44
Dihedral (kcal/mol)	47.20 ± 1.50	46.68 ± 1.56
Van Der Waal (kcal/mol)	-21.25 ± 3.32	-24.90 ± 4.11
Electrostatic (kcal/mol)	-354.25 ± 20.78	-359.41 ± 20.72
NOE (kcal/mol)	0.106 ± 0.018	0.041 ± 0.018
cDih (kcal/mol)	1.09 ± 0.63	0.256 ± 0.133
**RMS**		
Bonds (Å)	0.0131 ± 0.00073	0.01167 ± 0.00076
Angles (°)	1.590 ± 0.070	1.362 ± 0.112
Improper (Å)	1.32 ± 0.16	1.41 ± 0.33
Dihedral (Å)	43.30 ± 0.30	41.79 ± 0.71
NOE (Å)	0.0316 ± 0.0026	0.02311 ± 0.0053
cDih (Å)	0.725 ± 0.18	0.36 ± 0.11
**Pairwise RMSD**		
Backbone atoms (Å)	0.56 ± 0.21	0.89 ± 0.38
Heavy atoms (Å)	1.51 ± 0.37	2.23 ± 0.59
**MolProbity Analysis**		
Clashscore	10.54 ± 5.13	7.23 ± 6.94
Favoured Rotamers	99.45% ± 2.48%	95.00% ± 5.67%
Ramachandran Favoured	82.85 ± 5.86%	97.14 ± 5.86%
Ramachandran Outliers	0 ± 0%	0 ± 0%
Molprobity Score	2.23 ± 0.23	1.43 ± 0.65

**Extended Data Table 2:** Cryo-EM data collection, refinement, and validation statistics

	NET:AoIA
	PDB: 9K6X
	EMDB: 62139
**Data collection and processing**	
Magnification	105, 000
Voltage (kV)	300
Electron exposure (e-/Å^2^)	50
Defocus range (μm)	-1.2~-2.8
Pixel size (Å)	0.73
Symmetry imposed	C2
Initial particle images (no.)	5,205,798
Final particle images (no.)	112,752
Map resolution (Å)	2.32~3.50
FSC threshold	0.143
Map sharping B factor (Å^2^)	-99.8
**Refinement**	
Initial mode used	PDB: 8YR2
Model resolution (Å)	2.77
FSC threshold	0.143
Model-Map CC (mask)	0.66
Model composition	
Non-hydrogen atoms	9454
Protein residues	1124
Cholesterol	12
PG (18:2(9Z,12Z)/0:0)	2
H_2_O	8
B factors (Å^2^)	
Protein	47.40
Ligand	30.00
R.m.s.deviations	
Bond lengths	0.001
Bond angles	0.422
Validation	
MolProbity score	1.42
Clash score	7.35
Rotamer outliers (%)	1.06
Ramachandran plot	
Favored (%)	98.92
Allowed (%)	1.08
Disallowed (%)	0.00

**Extended Data Table 3:** List of confirmed and putative (in brackets) χ-conotoxin sequences

Peptide	Source organism	Sequence
**MrIA**	*Conus marmoreus*	NGV**CC**GYKL**C**HO**C**-
**MrIB**	*Conus marmoreus*	VGV**CC**GYKL**C**HO**C**-
**PnID**	*Conus pennaceus*	-ST**CC**GYRM**C**VP**C***
**AuID**	*Conus aulicus*	-SV**CC**GYKL**C**FO**C***
**AoIA**	*Conus araneosus*	–R**CC**GYKM**C**HO**C**-
**(Ar1248)**	*Conus araneosus*	-GV**CC**GVSF**C**YO**C**-
**(CMrVIA)**	*Conus marmoreus*	–V**CC**GYKL**C**HO**C**-
**(CMrX)**	*Conus marmoreus*	-GI**CC**GVSF**C**YO**C**-
**(Mr10.2)**	*Conus marmoreus*	–A**CC**VYKI**C**YO**C**-
**(Tx10b)**	*Conus textile*	-DP**CC**GYRM**C**VO**C***
**(Tx10c)**	*Conus textile*	-ZT**CC**GYRM**C**VO**C***

O = 4-hydroxyproline, Z = pyroglutamic acid, * *C*-terminal amidation
