# Supplementary material for: Structural and functional basis of antinociceptive action of χ-conotoxin AoIA at the noradrenaline transporter
Source: Nat Struct Mol Biol. 2026 Jul 21;33(9):1311–23. doi: 10.1038/s41594-026-01838-z (PMC13561870; doi:10.1038/s41594-026-01838-z)

nature structural & molecular biology

Supplementary information

https://doi.org/10.1038/s41594-026-01838-z

Structural and functional basis of antinociceptive action of χ-conotoxin AoIA at the noradrenaline transporter

In the format provided by the authors and unedited

Supplementary Table 1. System setup for MD.

Repeat		1	2	3	4
**Box dimensions**		(8.45 8.45 10.09) nm	(8.48 8.48 10.00) nm	(8.43 8.43 10.14) nm	(8.50 8.50 10.02) nm
**Total number of atoms**		74995	74899	74851	74896
**Number of water molecules**		14223	14191	14175	14190
**Salt concentration**		0.15 mM	0.15 mM	0.15 mM	0.15 mM
**Lipid composition**	**Upper leaflet**	POPC:POPE:CHOL=61:10:30	POPC:POPE:CHOL=61:10:30	POPC:POPE:CHOL=61:10:30	POPC:POPE:CHOL=61:10:30
	**Lower leaflet**	POPC:POPE:POPS:CHOL=20:36:15:31	POPC:POPE:POPS:CHOL=20:36:15:31	POPC:POPE:POPS:CHOL=20:36:15:31	POPC:POPE:POPS:CHOL=20:36:15:31

Supplementary Figure 1. Time course of the Root Mean Square Deviation from the start of the production simulations of 4 independent repeats. The thick line show running averages, while the thin semi-transparent lines show the individual datapoints.

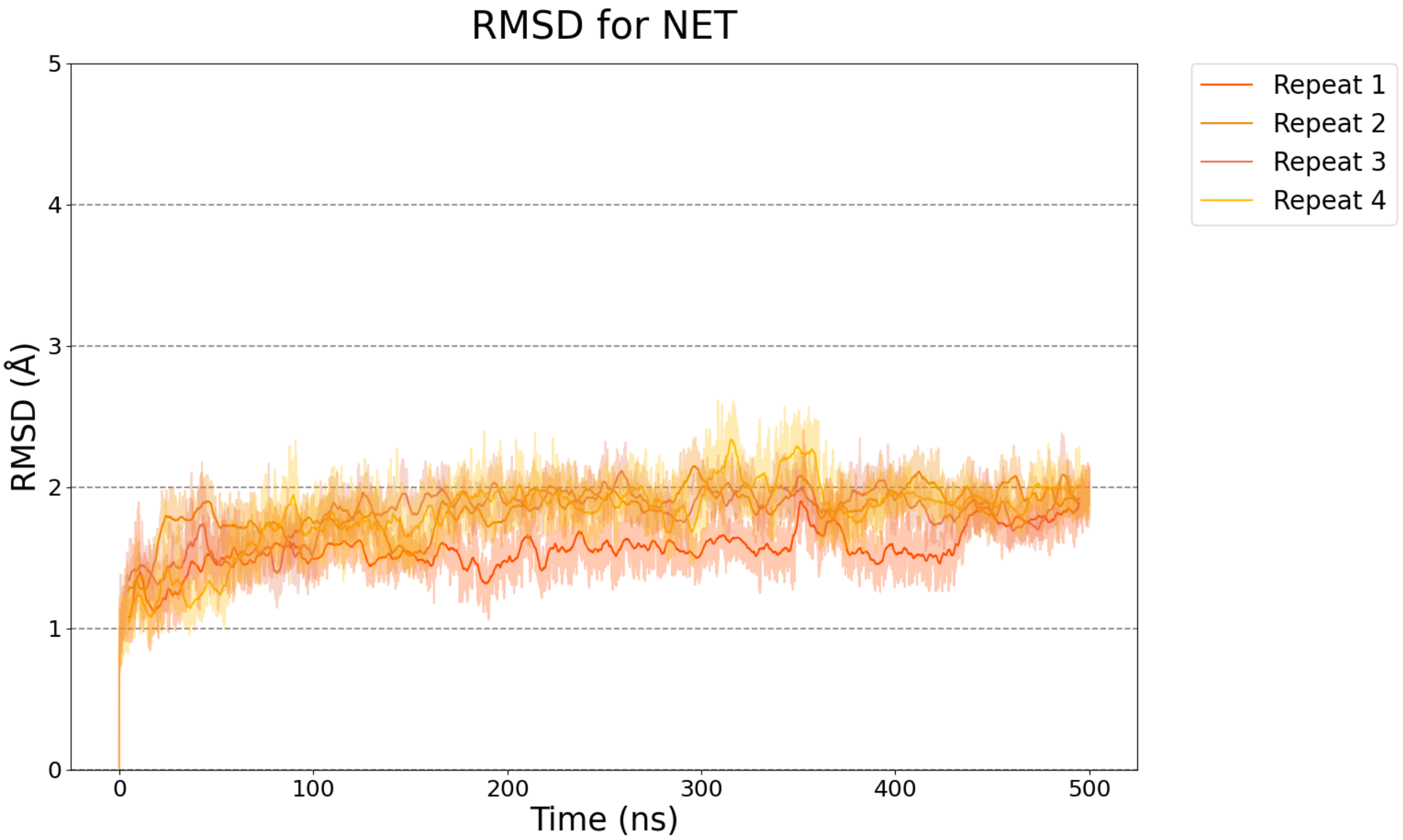

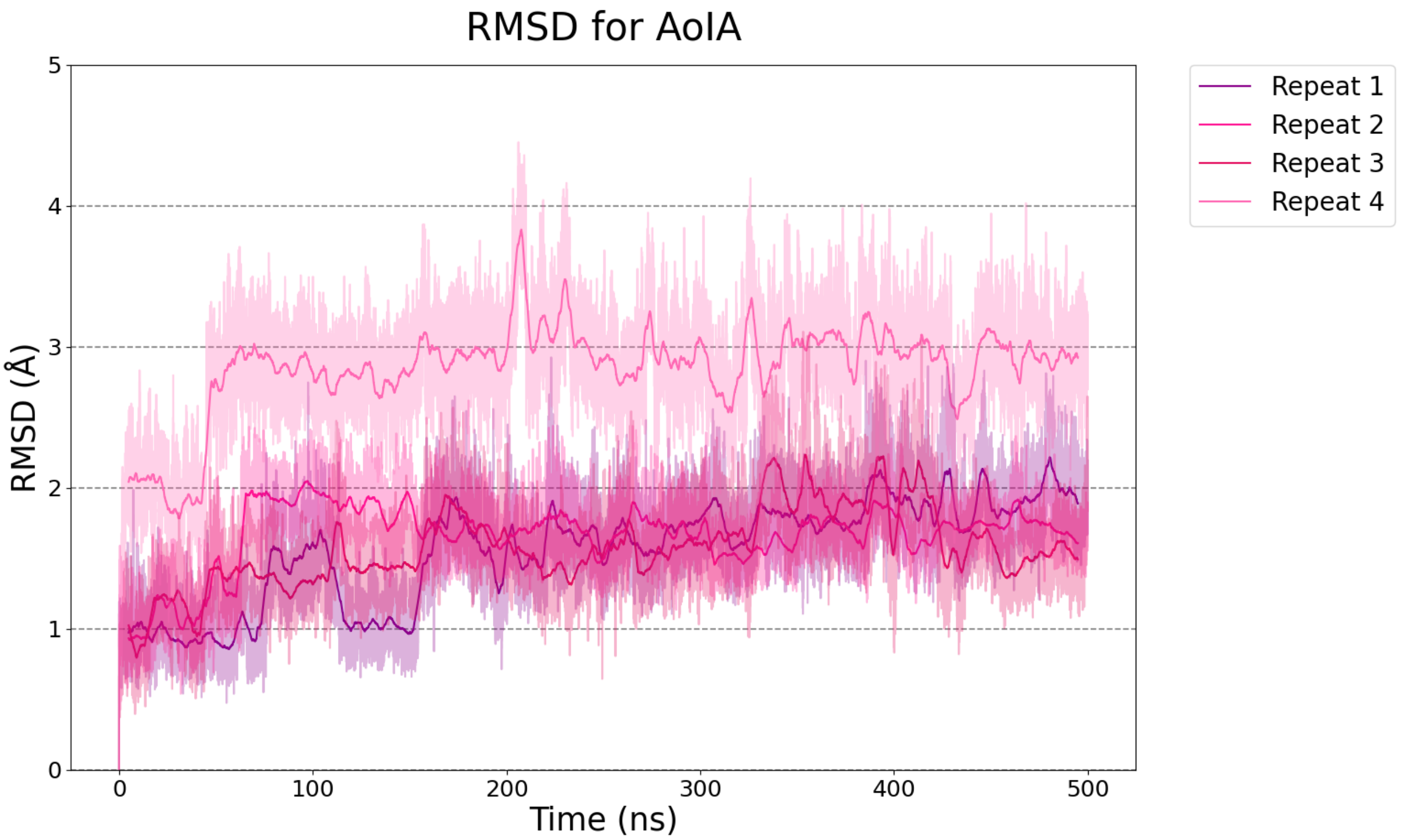

Supplement: Supplementary file 1 [file 41594_2026_1838_MOESM1_ESM.pdf]
