# Supplementary material for: Structural and functional basis of antinociceptive action of χ-conotoxin AoIA at the noradrenaline transporter
Source: Nat Struct Mol Biol. 2026 Jul 21;33(9):1311–23. doi: 10.1038/s41594-026-01838-z (PMC13561870; doi:10.1038/s41594-026-01838-z)
Supplement: Supplementary file 2 [file 41594_2026_1838_MOESM2_ESM.pdf]

nature portfolio

Corresponding author(s): Harald H. Sitte

Last updated by author(s): Feb 20, 2026

Reporting Summary

Nature Portfolio wishes to improve the reproducibility of the work that we publish. This form provides structure for consistency and transparency in reporting. For further information on Nature Portfolio policies, see our Editorial Policies and the Editorial Policy Checklist.

Statistics

For all statistical analyses, confirm that the following items are present in the figure legend, table legend, main text, or Methods section.

n/a	Confirmed	
☐	☒	The exact sample size (*n*) for each experimental group/condition, given as a discrete number and unit of measurement
☐	☒	A statement on whether measurements were taken from distinct samples or whether the same sample was measured repeatedly
☐	☒	The statistical test(s) used AND whether they are one- or two-sided<br>*Only common tests should be described solely by name; describe more complex techniques in the Methods section.*
☒	☐	A description of all covariates tested
☒	☐	A description of any assumptions or corrections, such as tests of normality and adjustment for multiple comparisons
☐	☒	A full description of the statistical parameters including central tendency (e.g. means) or other basic estimates (e.g. regression coefficient) AND variation (e.g. standard deviation) or associated estimates of uncertainty (e.g. confidence intervals)
☐	☒	For null hypothesis testing, the test statistic (e.g. F, t, r) with confidence intervals, effect sizes, degrees of freedom and P value noted<br>*Give P values as exact values whenever suitable.*
☒	☐	For Bayesian analysis, information on the choice of priors and Markov chain Monte Carlo settings
☒	☐	For hierarchical and complex designs, identification of the appropriate level for tests and full reporting of outcomes
☒	☐	Estimates of effect sizes (e.g. Cohen's *d*, Pearson's *r*), indicating how they were calculated

Our web collection on statistics for biologists contains articles on many of the points above.

Software and code

Policy information about availability of computer code

Data collection	Wallac 1450 MicroBeta Windows Workstation v4.01.014
Data analysis	MotionCor2, CryoSPARC v.4.3.1, ChimeraX 1.7 and 1.9, COOT, Phenix 1.17.1, Chromeleon 7.3.2, Shimadzu LabSolutions LC/GC v.5.7, Shimadzu Biotech MALDI-MS Application 2.9.4, Topspin 3.7.0, CARA 1.9.1, CNS 1.2, Clampex 11.3, Clampfit 11.3, GROMACS 2025.2, VMD 1.9.4, Pymol 2.5, GraphPad Prism v.5.04, v.9.4, and v.10.4.2, Excel 2016

For manuscripts utilizing custom algorithms or software that are central to the research but not yet described in published literature, software must be made available to editors and reviewers. We strongly encourage code deposition in a community repository (e.g. GitHub). See the Nature Portfolio guidelines for submitting code & software for further information.

Data

Policy information about availability of data

All manuscripts must include a data availability statement. This statement should provide the following information, where applicable:

- Accession codes, unique identifiers, or web links for publicly available datasets
- A description of any restrictions on data availability
- For clinical datasets or third party data, please ensure that the statement adheres to our policy

Peptide structures and chemical shifts have been submitted to the Protein Data Bank (PDB) and Biological Magnetic Resonance Bank: the globular isomer ArXAglob has been given accession codes 9PBO and 31256, respectively. The native ribbon isomer ArXA has been given the PDB and BMRB accession codes 9PAZ and 31255,

respectively. Protein structural data for hNET:χ-ArXA have been deposited to the Electron Microscopy Data Bank and the PDB under the accession codes: 62139 and 9K6X, respectively. Source data are provided with this paper. Other data supporting the findings of this study are available from the corresponding author upon request.

Research involving human participants, their data, or biological material

Policy information about studies with human participants or human data. See also policy information about sex, gender (identity/presentation), and sexual orientation and race, ethnicity and racism.

Reporting on sex and gender	N/A
Reporting on race, ethnicity, or other socially relevant groupings	N/A
Population characteristics	N/A
Recruitment	N/A
Ethics oversight	N/A

Note that full information on the approval of the study protocol must also be provided in the manuscript.

Field-specific reporting

Please select the one below that is the best fit for your research. If you are not sure, read the appropriate sections before making your selection.

☒ Life sciences ☐ Behavioural & social sciences ☐ Ecological, evolutionary & environmental sciences

For a reference copy of the document with all sections, see nature.com/documents/nr-reporting-summary-flat.pdf

Life sciences study design

All studies must disclose on these points even when the disclosure is negative.

Sample size	Sample size calculation was not performed.
Data exclusions	No data were excluded.
Replication	Each experiment was reproduced at least three times. Results were replicated.
Randomization	Randomization not relevant to study.
Blinding	Experiments were not blinded, treatment conditions (concentrations of ArXA, ArXAglob, and MrIA) and cell lines were known to the experimenter.

Reporting for specific materials, systems and methods

We require information from authors about some types of materials, experimental systems and methods used in many studies. Here, indicate whether each material, system or method listed is relevant to your study. If you are not sure if a list item applies to your research, read the appropriate section before selecting a response.

Materials & experimental systems

n/a	Involved in the study	
☒	☐	Antibodies
☐	☒	Eukaryotic cell lines
☒	☐	Palaeontology and archaeology
☐	☒	Animals and other organisms
☒	☐	Clinical data
☒	☐	Dual use research of concern
☒	☐	Plants

Methods

n/a	Involved in the study	
☒	☐	ChIP-seq
☒	☐	Flow cytometry
☒	☐	MRI-based neuroimaging

Eukaryotic cell lines

Policy information about cell lines and Sex and Gender in Research

Cell line source(s)	HEK293 cells were obtained from ATCC. Primary DRG neuronal cells were obtained from male wild-type C57BL/6 mice.
Authentication	HEK293 cells have been authenticated using highly polymorphic short tandem repeat loci (STRs). STR loci were amplified using the PowerPlex 16 HS System (Promega). Fragment analysis was done on an ABI3730xl (Life Technologies), and the resulting data were analyzed with GeneMarker HID software (Softgenetics). The results were compared with the Cellosaurus database in terms of matching to the cell line HEK293-DR-GFP-RAD51B-9 (RRID:CVCL_XX01): parental HEK293 cells (96.7%), stable HEK293 cell lines expressing YhDAT (90%), hNET (100%), and YhSERT (96.7%).
Mycoplasma contamination	Cell lines regularly tested negative for mycoplasma.
Commonly misidentified lines (See ICLAC register)	No commonly misidentified cell lines were used in this study.

Animals and other research organisms

Policy information about studies involving animals; ARRIVE guidelines recommended for reporting animal research, and Sex and Gender in Research

Laboratory animals	Experiments were performed in male SWISS mice (RjOrl:SWISS; 6-8 weeks old, 30-35 g body weight) purchased from Janvier Labs (Le Genest-Saint-Isle, France). Mice were group-housed in a temperature- (21-22°C) and humidity-controlled (60-70%) specific pathogen free room with a 12 h light/dark cycle and with free access to food and water. All behavioral experiments were performed during the light cycle.
Wild animals	No wild animals were used in this study.
Reporting on sex	We used male mice to establish pain and behavioral responses, as these types of studies are commonly reported using either male or female mice, or both sexes.
Field-collected samples	This study did not involve samples collected from the field.
Ethics oversight	All animal care and experimental procedures were in accordance with the ethical guidelines for the animal welfare standards of the European Communities Council Directive (2010/63/EU), and were approved by the Committee of Animal Care of the Austrian Federal Ministry of Science and Research.

Note that full information on the approval of the study protocol must also be provided in the manuscript.

Plants

Seed stocks	N/A
Novel plant genotypes	N/A
Authentication	N/A
