# Supplementary material for: Structural and functional basis of antinociceptive action of χ-conotoxin AoIA at the noradrenaline transporter
Source: Nat Struct Mol Biol. 2026 Jul 21;33(9):1311–23. doi: 10.1038/s41594-026-01838-z (PMC13561870; doi:10.1038/s41594-026-01838-z)
Supplement: Supplementary file 3 [file 41594_2026_1838_MOESM3_ESM.pdf]

nature portfolio

Peer Review File

Structural and functional basis of antinociceptive action of χ-conotoxin AoIA at the noradrenaline transporter

Corresponding Author: Professor Harald Sitte

Version 0:

Decision Letter:

4th Sep 2025

Dear Dr. Sitte,

Thank you again for submitting your manuscript "Structural and functional basis of antinociceptive action of χ-conotoxin ArXA at the noradrenaline transporter". I apologize for the delay in responding, which resulted from the difficulty in obtaining suitable referee reports. Nevertheless, we now have comments (below) from the 3 reviewers who evaluated your paper. In light of those reports, we remain interested in your study and would like to see your response to the comments of the referees, in the form of a revised manuscript.

You will see that while reviewers appreciate the results, they raise several concerns which will need to be addressed in a revision. Specifically, we ask that you address the comments of reviewer #1 regarding the peptide action int the somatosensory circuit. You will also see that multiple reviewers ask to rule out reactivity with other targets, eg Navs – we agree that this will be needed to further strengthen the manuscript. In line with reviewer's #2 comments, please also provide further support for the mode of inhibition of ArXA. Further, you will see that reviewer #3 deems it important for the impact of the manuscript to assess the neuropathic pain model – while we appreciate this could be challenging, but agree that it would strengthen the manuscript, and would encourage you to explore this avenue further.

Please be sure to address/respond to all concerns of the referees in full in a point-by-point response and highlight all changes in the revised manuscript text file. If you have comments that are intended for editors only, please include those in a separate cover letter.

We are committed to providing a fair and constructive peer-review process. Do not hesitate to contact us if there are specific requests from the reviewers that you believe are technically impossible or unlikely to yield a meaningful outcome.

We expect to see your revised manuscript within 3-6 months. If you cannot send it within this time, please contact us to discuss an extension; we would still consider your revision, provided that no similar work has been accepted for publication at NSMB or published elsewhere.

As you already know, we put great emphasis on ensuring that the methods and statistics reported in our papers are correct and accurate. As such, if there are any changes that should be reported, please submit an updated version of the Reporting Summary along with your revision.

Please follow the links below to download these files:

Reporting Summary:
https://www.nature.com/documents/nr-reporting-summary.pdf

Please note that the form is a dynamic 'smart pdf' and must therefore be downloaded and completed in Adobe Reader.

When submitting the revised version of your manuscript, please pay close attention to our href="https://www.nature.com/nature-portfolio/editorial-policies/image-integrity">Digital Image Integrity Guidelines. and to the following points below:

-- that unprocessed scans are clearly labelled and match the gels and western blots presented in figures. Please note that all key data shown in the main figures as cropped gels or blots should be presented in uncropped form, with molecular weight markers. While these data can be displayed in a relatively informal style, they must refer back to the relevant figures. These data should be submitted as source data with the last revision, prior to acceptance.
-- that control panels for gels and western blots are appropriately described as loading on sample processing controls
-- all images in the paper are checked for duplication of panels and for splicing of gel lanes.
-- For any revision that includes light microscopy data, we ask our authors to please include a completed light microscopy reporting table [https://www.nature.com/documents/Light_microscopy_reporting_table.xlsx] to ensure the methods are described thoroughly. The table will be available to reviewers and ultimately published should the manuscript be accepted at the journal.

EXTENDED DATA FIGURES

When re-submitting your manuscript, please ensure that any supplementary figures and tables that are crucial to the manuscript's conclusions are converted into Extended Data figures and tables to increase visibility of these data. Extended Data figures and tables are online-only (present in the online PDF and full-text HTML versions of the paper), peer-reviewed display items that provide essential background to the article but are not included in the main article due to space constraints. A maximum of ten Extended Data display items (figures and tables) is permitted.

Finally, please ensure that you retain unprocessed data and metadata files after publication, ideally archiving data in perpetuity, as these may be requested during the peer review and production process or after publication if any issues arise.

If there are additional or modified structures presented in the final revision, please submit the corresponding PDB validation reports.

SOURCE DATA: authors must provide, in tabular form, the data underlying the graphical representations used in figures. This is to further increase transparency in data reporting, as detailed in this editorial (http://www.nature.com/nsmb/journal/v22/n10/full/nsmb.3110.html). Spreadsheets can be submitted in excel format. Only one (1) file per figure is permitted; thus, for multi-paneled figures, the source data for each panel should be clearly labeled in the Excel file; alternately the data can be provided as multiple, clearly labeled sheets in an Excel file. When submitting files, the title field should indicate which figure the source data pertains to. We encourage our authors to provide source data at the revision stage, so that they are part of the peer-review process.

Data availability: this journal strongly supports public availability of data. All data used in accepted papers should be available via a public data repository, or alternatively, as Supplementary Information. If data can only be shared on request, please explain why in your Data Availability Statement, and also in the correspondence with your editor. Please note that for some data types, deposition in a public repository is mandatory - more information on our data deposition policies and available repositories can be found below:
https://www.nature.com/nature-research/editorial-policies/reporting-standards#availability-of-data

We require deposition of coordinates (and, in the case of crystal structures, structure factors) into the Protein Data Bank with the designation of immediate release upon publication (HPUB). Electron microscopy-derived density maps and coordinate data must be deposited in EMDB and released upon publication. Deposition and immediate release of NMR chemical shift assignments are highly encouraged. Deposition of deep sequencing and microarray data is mandatory, and the datasets must be released prior to or upon publication. To avoid delays in publication, dataset accession numbers must be supplied with the final accepted manuscript and appropriate release dates must be indicated at the galley proof stage.

While we encourage the use of color in preparing figures, please note that this will incur a charge to partially defray the cost of printing. Information about color charges can be found at http://www.nature.com/nsmb/authors/submit/index.html#costs

Nature Structural & Molecular Biology is committed to improving transparency in authorship. As part of our efforts in this direction, we are now requesting that all authors identified as 'corresponding author' on published papers create and link their Open Researcher and Contributor Identifier (ORCID) with their account on the Manuscript Tracking System (MTS), prior to acceptance. This applies to primary research papers only. ORCID helps the scientific community achieve unambiguous attribution of all scholarly contributions. You can create and link your ORCID from the home page of the MTS by clicking on 'Modify my Springer Nature account'. For more information please visit please visit www.springernature.com/orcid.

Please use the link below to submit your revised manuscript and related files:

Link Redacted

Note: This URL links to your confidential home page and associated information about manuscripts you may have submitted, or that you are reviewing for us. If you wish to forward this email to co-authors, please delete the link to your homepage.

We look forward to seeing the revised manuscript and thank you for the opportunity to review your work.

Sincerely,
Kat

Katarzyna Ciazynska, PhD
(she/her)
Senior Editor
Nature Structural & Molecular Biology
https://orcid.org/0000-0002-9899-2428

Referee expertise:

Referee #1: pharmacology, conotoxins

Referee #2: cryo-EM, membrane proteins

Referee #3: neurobiology, pharmacology

Reviewers' Comments:

Reviewer #1 (Remarks to the Author):

Summary of Key Results:
1) The structure of a chi-conotoxin bound to NET is presented.
2) Similar to previous work on chi-conotoxins, the peptide presented here also exhibit antinociceptive activity.

Originality and significance:
1) Again, the structure of the transporter bound to a ligand is a significant advancement in the field of conotoxins and in NET research.
2) Key results were mainly consistent with previously published work.

Data & methodology: validity of approach, quality of data, quality of presentation
1) Rationale for using a specific method needs to be presented.
a. Why is the peptide delivered subcutaneously rather than IT when MrIa was shown to be more effective via IT.
b. Why was NE not used in the transporter assay.
c. Why was post mortem analysis of the spinal cord not performed considering that this tissue is where chi-conotoxins have their most potent effect.
2) Quality of data and presentation is satisfactory.

Appropriate use of statistics and treatment of uncertainties
1) This is satisfactory.

Conclusions: robustness, validity, reliability
1) The main conclusion that the peptide acts primarily on the somatosensory circuit needs more experimental support.
a. The activity of the peptide at the spinal level was not measured thus the possibility that it is more potent and thus exerts most of its activity in the spinal cord cannot be eliminated at this point. Work in the related peptide, MrIa has demonstrated the IT delivery is more effective at attenuating nociception thus the same can be said for the peptide described in this work.
b. It is interesting to explain, based on the structure presented in the paper, why the peptide is more potent than MrIA.

Suggested improvements: experiments, data for possible revision.
1) Please see attachment.

References: appropriate credit to previous work?
1) Please state in the result section how the peptide sequence was obtained. Is this from the Gupta paper?

Clarity and context: lucidity of abstract/summary, appropriateness of abstract, introduction and conclusions
1) This is satisfactory. Please see above comments for improving the conclusion

Reviewer #2 (Remarks to the Author):

The noradrenaline transporter (NET) is responsible for clearing noradrenaline from the synaptic cleft, thereby terminating its action on postsynaptic receptors. NET inhibitors have shown considerable promise in the treatment of depression and pain. In this study, Belleza et al. report the identification of a novel conotoxin, ArXA, based on its sequence similarity to previously characterized conotoxins. ArXA demonstrates greater potency in inhibiting NET compared to MrIA. Furthermore, the cryo-EM structure of the NET–ArXA complex provides valuable insight into the molecular mechanism underlying ArXA-mediated inhibition. ArXA also exhibits antinociceptive effects in an inflammatory pain model. Overall, the manuscript is well-written and logically organized. However, several concerns need to be addressed before it can be considered for publication.
Major:
1. The previously published structure of NET bound to MrIA, which is closely related to the structure reported in this study, should be discussed in the Introduction. A comparison between the two structures should be presented in the main figures and described in the main text.
2. While previous studies have characterized MrIA and related toxins as allosteric inhibitors, this work proposes a mixed competitive/noncompetitive mode of inhibition for ArXA. The evidence for competitive inhibition is based on a slight increase in Km at higher ArXA concentrations; however, this difference is minimal and maybe not statistically significant. Although the structural data show that ArXA extends into the S1 binding site and partially overlaps with the amine moiety of norepinephrine (NE), the transporter's dynamic nature suggests that minor conformational adjustments could still permit simultaneous binding of ArXA and NE. Therefore, the conclusion that ArXA competes with NE is not fully convincing. Additional experiments with higher ArXA concentrations should be performed to determine whether the increase in Km reaches statistical significance, and molecular dynamics simulations are recommended to further evaluate the structural compatibility of ArXA and NE.
3. Since ArXA demonstrates greater potency than MrIA in inhibiting NET transport, the authors should elaborate on the underlying reasons for this difference, drawing on insights from structural analysis and mutagenesis experiments.
4. The authors evaluated the antinociceptive effect of ArXA in male mice. To better contextualize these findings, they should report the sequence similarity between mouse and human NET and clarify ArXA's potency for mouse NET. Although they ruled out ArXA's action on opioid receptors and TRPV1, its antinociceptive effect could still involve other targets, which the authors should address or exclude.

Minor
1. The two-dimensional 15N HSQC spectrum of ArXA, along with its assignment, should be provided in the supplementary figures.
2. Please clarify what the dashed lines in Figure 3 represent. If they indicate hydrogen bonds, why are dashed lines shown between sulfur and oxygen atoms in Figure 3G?
3. The side chains and labels in Figure 4 are unclear and should be redrawn for better readability. Additionally, a sequence alignment of NET, SERT, and DAT should be included in the supplementary materials.
4. The scale bars are missing in the 2D averages shown in Extended Figure 2A and should be added. A model to map the FSC should also be included.
5. The IC50 values for the competition curves should be summarized in an extended table.

Reviewer #3 (Remarks to the Author):

In this study, the authors synthesized and characterized χ-conotoxin (χ-ArXA), a peptide that inhibits the norepinephrine transporter (NET) without affecting opioid receptors or TRP channels (TRPA1 and TRPV1). Importantly, they resolved the cryo-EM structure of the human NET:χ-ArXA complex at high resolution, revealing an atypical binding mode that engages both the central binding site and the outer vestibule of the transporter. Furthermore, subcutaneous administration of χ-ArXA produced a significant antinociceptive effect in a mouse formalin-induced inflammatory pain model. These findings are both novel and significant, providing new mechanistic insights into χ-conotoxins and highlighting their potential as leads for the development of non-opioid analgesics.

Points for consideration:

1. Introduction: ω-conotoxin MVIIA is approved for clinical use as the intrathecally administered drug ziconotide). A comparison between ω-conotoxin and χ-conotoxin (χ-ArXA) in their analgesic efficacy and side effect profile will strengthen this study.

2. It is impressive to solve cryo-EM) structure of the human NET:ArXA complex and demonstrate in vitro activity of NET inhibition. However, ArXA may produce antinociception via additional targets in vivo. I wonder if α2-AR antagonist or knockout can block the analgesic effects of ArXA.

3. NET inhibitors are used to treat neuropathic pain. It will facilitate translation if the authors can assess a neuropathic pain model. Subcutaneous injection may only produce transient pain relief.

4. Limitations of this study should be discussed.

Version 1:

Decision Letter:

Our ref: NSMB-A51419A

7th Apr 2026

Dear Dr. Sitte,

Thank you for submitting your revised manuscript "Structural and functional basis of antinociceptive action of χ-conotoxin AoIA at the noradrenaline transporter" (NSMB-A51419A). Please accept our apologies for the delay in this decision. The manuscript has now been seen by the original referees and their comments are below. The reviewers find that the paper has improved in revision, and therefore we'll be happy in principle to publish it in Nature Structural & Molecular Biology, pending minor revisions to satisfy the referees' final requests and to comply with our editorial and formatting guidelines.

Specifically, you will see that Reviewer 2 retains certain concerns about the structural model, which will need to be addressed along with our other requests once we have run the entirety of our checks. In particular, you will be asked to please improve the clashscore of the model, in line with comments of Reviewer 2 regrading the water molecules, and update any corresponding figures.

We are now performing detailed checks on your paper and will send you a checklist detailing our editorial and formatting requirements in about a week. Please do not upload the final materials and make any revisions until you receive this additional information from us.

To facilitate our work at this stage, it is important that we have a copy of the main text as a word file. If you could please send along a word version of this file as soon as possible, we would greatly appreciate it; please make sure to copy the NSMB account (cc'ed above).

Thank you again for your interest in Nature Structural & Molecular Biology Please do not hesitate to contact me if you have any questions.

Sincerely,

Yue Feng, PhD
(she/her)
Associate Editor
Nature Structural & Molecular Biology
ORCID: 0009-0002-9697-1903

Reviewer #1 (Remarks to the Author):

The authors presented a structure of chi-conotoxin Aola bound to human NET. Similar to previously published article the data presented here described key interactions between the transporter and the toxin that could explain the high degree of selectivity of chi-Aola and most likely as yet to be discovered chi-conotoxins towards NET.

Another important discovery in this paper is the apparent antinociceptive activity of the conotoxin presumably from targeting NET in the peripheral nervous system.

The authors have satisfactorily addressed my comments and I recommend this research for publication

Reviewer #2 (Remarks to the Author):

The authors have addressed most of my concerns. I have a minor suggestion regarding the structural model: the clash score is unusually high, which may be due to misplacement of water molecules that create steric clashes with nearby residues. In cryo-EM structures, particularly at moderate resolution, water molecules should be modeled cautiously, as spherical densities can represent noise and often appear throughout the map. In Figure 4A, the density assigned to some water molecules appears too bulky to correspond to water. I recommend that most water molecules be removed or re-evaluated in the structural model.The Figure 4 should be updated accordingly.

Reviewer #3 (Remarks to the Author):

The revision is improved. I have no further comments.

Version 2:

Decision Letter:

3rd Jun 2026

Dear Dr. Sitte,

We are now happy to accept your revised paper "Structural and functional basis of antinociceptive action of χ-conotoxin AoIA at the noradrenaline transporter" for publication as an Article in Nature Structural & Molecular Biology.

Acceptance is conditional on the manuscript's not being published elsewhere and on there being no announcement of this work to the newspapers, magazines, radio or television until the publication date in Nature Structural & Molecular Biology.

Over the next few weeks, your paper will be copyedited to ensure that it conforms to Nature Structural & Molecular Biology style. Once your paper is typeset, you will receive an email with a link to choose the appropriate publishing options for your paper and our Author Services team will be in touch regarding any additional information that may be required.

After the grant of rights is completed, you will receive a link to your electronic proof via email with a request to make any corrections within 48 hours. If, when you receive your proof, you cannot meet this deadline, please inform us at rjsproduction@springernature.com immediately.

You will not receive your proofs until the publishing agreement has been received through our system.

Due to the importance of these deadlines, we ask that you please let us know now whether you will be difficult to contact over the next month. If this is the case, we ask you provide us with the contact information (email, phone and fax) of someone who will be able to check the proofs on your behalf, and who will be available to address any last-minute problems.

To assist our authors in disseminating their research to the broader community, our SharedIt initiative provides all co-authors with the ability to generate a unique shareable link that will allow anyone (with or without a subscription) to read the published article. Recipients of the link with a subscription will also be able to download and print the PDF.

As soon as your article is published, you can generate your shareable link by entering the DOI of your article here: http://authors.springernature.com/share. Corresponding authors will also receive an automated email with the shareable link

Note the policy of the journal on data deposition: http://www.nature.com/authors/policies/availability.html.

Your paper will be published online soon after we receive proof corrections and will appear in print in the next available issue. You can find out your date of online publication by contacting the production team shortly after sending your proof corrections.

You may wish to make your media relations office aware of your accepted publication, in case they consider it appropriate to organize some internal or external publicity. Once your paper has been scheduled you will receive an email confirming the publication details. This is normally 3-4 working days in advance of publication. If you need additional notice of the date and time of publication, please let the production team know when you receive the proof of your article to ensure there is sufficient time to coordinate. Further information on our embargo policies can be found here: https://www.nature.com/authors/policies/embargo.html

You can now use a single sign-on for all your accounts, view the status of all your manuscript submissions and reviews, access usage statistics for your published articles and download a record of your refereeing activity for the Nature journals.

If you have not already done so, we strongly recommend that you upload the step-by-step protocols used in this manuscript to the Protocol Exchange. Protocol Exchange is an open online resource that allows researchers to share their detailed experimental know-how. All uploaded protocols are made freely available, assigned DOIs for ease of citation and fully searchable through nature.com. Protocols can be linked to any publications in which they are used and will be linked to from your article. You can also establish a dedicated page to collect all your lab Protocols. By uploading your Protocols to Protocol Exchange, you are enabling researchers to more readily reproduce or adapt the methodology you use, as well as increasing the visibility of your protocols and papers. Upload your Protocols at www.nature.com/protocolexchange/. Further information can be found at www.nature.com/protocolexchange/about.

An online order form for reprints of your paper is available at https://www.nature.com/reprints/author-reprints.html. Please let your coauthors and your institutions' public affairs office know that they are also welcome to order reprints by this method.

Authors may need to take specific actions to achieve compliance with funder and institutional open access mandates. If your research is supported by a funder that requires immediate open access (e.g. according to Plan S principles or the NIH public access policy) then you should select the gold OA route, and we will direct you to the compliant route where possible. Because authors warrant under our subscription licensing terms that they haven’t committed to licensing any version of their article under a licence inconsistent with the terms of our agreement – including the applicable embargo period – publication under the subscription model isn’t suitable for authors whose funders require no embargo.

In approximately 10 business days you will receive an email with a link to choose the appropriate publishing options for your paper and our Author Services team will be in touch regarding any additional information that may be required.

You will not receive your proofs until the publishing agreement has been received through our system.

If you have any questions about our publishing options, costs, Open Access requirements, or our legal forms, please contact ASJournals@springernature.com

Sincerely,

Katarzyna Ciazynska, PhD
(she/her)
Senior Editor
Nature Structural & Molecular Biology
https://orcid.org/0000-0002-9899-2428

Click here if you would like to recommend Nature Structural & Molecular Biology to your librarian:
http://www.nature.com/subscriptions/recommend.html#forms

Open Access This Peer Review File is licensed under a Creative Commons Attribution 4.0 International License, which permits use, sharing, adaptation, distribution and reproduction in any medium or format, as long as you give appropriate credit to the original author(s) and the source, provide a link to the Creative Commons license, and indicate if changes were made.
In cases where reviewers are anonymous, credit should be given to 'Anonymous Referee' and the source.
The images or other third party material in this Peer Review File are included in the article’s Creative Commons license, unless indicated otherwise in a credit line to the material. If material is not included in the article’s Creative Commons license and your intended use is not permitted by statutory regulation or exceeds the permitted use, you will need to obtain

permission directly from the copyright holder.
To view a copy of this license, visit https://creativecommons.org/licenses/by/4.0/

Dear Editor, dear Reviewers,

Many thanks for your careful examination of our manuscript. We appreciate the comments and suggestions you have given, and we acknowledge how these could contribute towards the improvement of our study. While some suggestions can be addressed directly, others presented challenges considering the current scope of our study, the resources of our consortium, and a reasonable period for completion. Following the guidance of the Editor, we have prepared a revised manuscript that addresses the Reviewers' concerns, while staying within the practical constraints of our current resources and a reasonable time frame. In this document, we highlight the major changes we have made in the revised manuscript, and provide you with our response to each individual comment the Reviewers have raised.

In conclusion, we would like to stress that this study may serve as a starting point of a couple of follow-up studies, some of which have already been started, also following the suggestions of the reviewers. Therefore, we would like to – again – thank all our reviewers at this point for their very valuable comments.

Oliver Belleza, Zhang Heng, Eric Xu, Harald Sitte, on behalf of the entire team

Reviewer 1

General comments:

1) Please rename the peptide chi-AoIa, there is already a published peptide, a ArIb from Conus arenatus that is given the name designation Ar. Framework X was the previous cysteine pattern assignment given to chi conotoxins, but this has been changed to framework I. This name designation will make this new peptide name consistent with previously described chi conotoxins.

Response: According to the reviewer's suggestion, we have renamed the peptide χ-AoIA. This name, along with the shortened version AoIA, is now used throughout the entire manuscript.

2) Rationalize why the peptide was delivered subcutaneously rather than intrathecally, given that MrIa was shown to be more effective via IT. It is possible that a more potent antinociceptive activity will be observed via IT delivery.

Response: While it is indeed possible that our peptide χ-AoIA presents a more potent antinociceptive activity via intrathecal (IT) delivery, one of our objectives in this study was not to simply replicate previous studies that have been done on the analgesic effects of χ-conotoxins, but also to explore alternatives. AoIA does not merely replicate the effects of previous chi-conotoxins. Unlike earlier studies using IT administration (e.g., MrIA), AoIA demonstrates robust antinociception via subcutaneous (s.c.) delivery, a route that is certainly more practical. Our cellular assays further demonstrate that AoIA inhibits NET with 10-fold greater potency than MrIA, likely explaining its efficacy via peripheral administration. While we have already shown that AoIA does not cross the blood-brain barrier at a detectable level (Figure S6), we believe further IT experiments are not needed to support our claims; in fact, given the demonstrated advantage of the subcutaneous route, AoIA provides an alternative route that will be more favorable to translation. Compared with s.c., i.v. or i.p. routes, IT administration is invasive, technically demanding, less acceptable to patients, and carries greater risks of complications such as headache, infection, or nerve injury: Thus, the s.c. delivery presents a clear advantage over the IT delivery.

3) Why was MPP+ used for the transporter assay and not NE?

Response: MPP^+ is a common substrate used for studying the uptake of monoamine transporters. Unlike NE and other related catecholamine substrates, MPP^+ is chemically stable, and is therefore more amenable to bench handling and reliably producing consistent results in our cellular assays. We have a long-standing experience in the laboratory with utilizing it over the past 25 years.

4) Based on the structure, can you please explain why AoIa is more potent than than MrIa? Do amino acids in the second loop and in the N-terminal affect potency? Previous work showed that -GYKL- in MrIa is the major determinant of potency.

Response: Our determined structures indeed support the notion that the GYKL residues of MrIA, GYKM in the case of AoIA, surrounding the hairpin loop form critical interactions with the central binding site (S1) of NET. In addition, the remaining parts of the peptide also interact with the vestibular site of NET. Regarding the molecular basis of the potency of AoIA in comparison to MrIA,

this is a more challenging (and also interesting) question that we are indeed pursuing concurrently as a follow-up project, involving computational pharmacology, mutagenesis, and other wet lab approaches. Thank you for pointing this out.

Specific comment:

Lines 118-119: How was AoIa sequence obtained? Was this from Gupta's work or your own work? This may have been described in the methods, but it would be good to describe that here as well and cite Gupta if indeed this was from their work.

Response: The sequence was indeed obtained from Gupta's work which was already cited in the original text. This is more explicitly stated in the revised text: "The peptide originally isolated by Gupta et al. from *Conus araneosus* with the sequence RCCGYKMCHOC, where O stands for 4-hydroxyproline (Hyp), was found to possess the same disulfide connectivity as other χ-conotoxins." (Page 5, last paragraph)

Lines 137-139: Is this air oxidation? Please briefly describe that here.

Response: Oxidation was performed in an aqueous solution containing 30% DMSO. It is now more explicitly stated in the main text that oxidation is DMSO-assisted. Experimental details are already described in the Methods section.

Main text: "A non-selective DMSO-assisted oxidative folding method yielded two major isomeric peptide products, observed as the two major peaks in the HPLC chromatograms (Figure 1B)." (page 6, second paragraph).

Methods section, sub-section "*Non-selective oxidative synthesis of AoIA globular and ribbon isomers" (page 33).*

Lines 139-141: Indicate %B90 where the peptide/s elute.

Response: Peak A (AoIA^{glob}): t_R = 16.6 min, %B = 26.1; Peak B (AoIA): t_R = 17.1 min, %B = 26.4. These values have been added to the main text: "The first peak "Peak A" has a HPLC retention time (tR) of 16.6 min and elutes at 26.1% Solvent B, while the second peak "Peak B" elutes at tR = 17.1 min and 26.4% Solvent B." (Page 6, second paragraph)

Lines 205-206; 211-212: You mentioned that the peptide was tested in the calcium imaging of DRG neurons but that result was not presented here. Although TRP channels have been associated with nociception, other ion channels such as Nav1.8 could also be a target of antinociceptive compound. That should be considered in the discussion. Any activity on DRG could have implications in somatosensation.

Response: A more thorough description of the calcium imaging experiments on DRG neurons is now provided on Page 10, second paragraph. However, in order to follow the advice of the Reviewer, we

conducted additional experiments which exclude an action on the dominant voltage-gated sodium channels in sensory neurons (Nav1.7 and Nav1.8). These new results were added to Figure 3 in Panels F-H.

Line 230: Please explain why formaldehyde was used. Will it not kill the DRG neurons?

Response: Short-term exposure to formaldehyde was used to investigate intracellular calcium handling. Short-term formaldehyde exposure in the used concentration of 20 mM is not cytotoxic, but elicits robust and reversible calcium transients in neurons (Fisher et al., 2014; Fischer et al., 2015; Hoffmann et al., 2022), which come from the endoplasmic reticulum due to direct inhibition of SERCA (Fischer et al., 2015). The experiments were conducted in TRPA1 knockout animals, as SERCA effects are masked by the more formaldehyde-sensitive TRPA1. These experiments exclude SERCA as a target of AoIA, which is now clearly communicated in the manuscript. Importantly, this in not in conflict with behavioral observations in the formalin test, where all known antinociceptive agents reduce the second phase.

Lines 386-387: Is activity in the tail flick assay a measure of peripheral mechanisms, specifically thermal nociception? No activity in this assay suggests that the chi-ctx does not act on the peripheral circuit of thermal nociception. Tail flick is a reflex response and is mediated at the spinal level, but for this to happen, a peripheral stimuli must be detected first. Please clarify.

Response: While the tail flick assay requires peripheral detection of a thermal stimulus (D'Amour & Smith, 1941), the behavioral output (withdrawal of the tail) is governed predominantly by a rapid spinal reflex arc (Bannon & Malmberg, 2007) and remained intact in spinalized cats (Irwin et al., 1951). There are differential supraspinal contributions to the formalin and the tail flick test (Abbott & Melzack, 1982). Both tests also rely on peripheral input, and are therefore also sensitive to peripheral action, which indeed precludes pinpointing a site of action. This line has been removed, and a new paragraph describing the analgesic mechanisms surrounding AoIA activity has been added to the Discussion (Pages 30-31, starting with: "Our behavorial studies. . ." on Page 30, second paragraph).

Line 457-460: Couldn't really say this as there are only 3 published chi-Ctx's and there are others that could prove to be more potent. Please revise this sentence.

Response: The sentence has now been revised to not state any comparisons with other chi-conotoxins besides MrIA. It reads now as follows: "It is worth nothing that AoIA exhibits around 10-fold increased potency compared to MrIA in our NET assays, which could indicate that it may be a suitable candidate for further development and applications." (Page 28)

Line 465-467: I think one of your references in fact said this. MrIa partially overlaps the NE binding site thus not exactly an allosteric modulator. Please revise and reference that work.

Response: The work by Sharpe et al. (2001), Bryan-Lluka et al. (2003), Paczkowski et al. (2007), and also Hu et al. (2024) were already referenced in relation to the fact that MrIA was previously described as an allosterically binding, non-competitive inhibitor. Our sentence makes clear that the non-competitive kinetic model proposed for MrIA may be incomplete and needed to be revised. At higher concentrations, AoIA behaves as a competitive inhibitor, which could also be true for other chi-conotoxins like MrIA. We are the first to describe this mixed competitive mode of inhibition of chi-conotoxins. Thus, we have decided that the sentence remains the same.

Lines 501-503: Other potential targets have not been ruled out in this work. For example Nav 1.8 which is also widely expressed in DRG neurons. Please revise this sentence.

Response: We have tested now other potential targets, including voltage-gated sodium channels Nav1.7 and Nav1.8, and alpha2-adrenergic receptors. The sentence has been revised. Another sentence referencing previous work that determined chi-conotoxins operate through adrenergic mechanisms directly follows. The newly added text reads as follows: "The lack of activity on other common targets involved in nociception, such as the opioid receptors, TRP channels as well as voltage-gated sodium and calcium channels (Figure 3), suggests that NET inhibition is its primary mechanism for analgesia. Indeed, it has been previously suggested for MrIA and Xen2174 that the analgesic effect of χ-conotoxins proceeds through adrenergic mechanisms in rodent models of pain." (Page 29, last paragraph)

Lines 514-516. Although not present in the brain it could still be present in the spinal cord and that could still indicate central activity. Why was this not done considering that MrIa was, as previously said is more effective when delivered IT.

Response: The likelihood of detecting the peptide in the spinal cord—while it was not found elsewhere in the central nervous system—is extremely low. Consequently, we did not pursue this line of investigation further. As we are not currently planning additional animal experiments within the scope of this project, we are unable to address this question at present.

Line 517: Again, this is a reflex response to peripheral nociceptive input. No input then no reflex.

Response: Please see also our reply above (Page 4 of this document).

Our conotoxin is a selective NET inhibitor. NET inhibition is known to enhance descending noradrenergic inhibitory pathways, which are preferentially engaged during persistent, inflammatory, or sensitization dependent nociception rather than during acute spinal reflexes. This is well reported in the literature (Pertovaara 2006; Millan 2002; Rahman et al. 2008). According to Iyengar et al. (2004), Pertovaara (2006), and Millan (2002), this pattern is in line with the known pharmacology of NET inhibitors, including those used in clinical settings. These inhibitors are effective in models of inflammatory or tonic pain where descending noradrenergic pathways are involved, but they usually have modest effects in acute thermal reflex assays like tail flick.

In contrast, the formalin test, particularly phase II, involves peripheral inflammatory processes, sustained nociceptor activation, and central sensitization, all of which are highly sensitive to modulation by enhanced noradrenergic signaling. The robust effect of the conotoxin in the formalin test is therefore plausibly a result of NET inhibition.

Thus, the absence of activity in the tail flick assay does not indicate that the conotoxin lacks peripheral action or does not influence peripheral nociceptors. It rather reflects the fact that NET mediated analgesic mechanisms are not strongly recruited in acute spinal reflex responses to thermal stimuli, whereas they are highly relevant in inflammatory pain states, as shown by the formalin test.

This line has been removed and a new paragraph is put in its place to discuss the mechanisms surrounding the analgesic activity of AoIA (Pages 30-31).

Lines 523-524: Given this, why was only the brain tissue analyzed as stated in line 515? The spinal cord should have been analyzed as well.

Response: Since we examined the brain tissue, and could not detect AoIA at meaningful concentrations, we refrained from examining spinal cord tissue (as also stated above to your previous question). Both the brain and spinal cord are shielded by the same blood-brain barrier. We therefore assume that AoIA would not be detected in the spinal cord either.

Lines 525-527: Are both NET and a22--ARAR also expressed in central projections of sensory neurons?

Response: This line of text is meant to guide the reader towards an exploration of the plausible peripheral mechanisms by which analgesia can be elicited through NET inhibition and/or α2-AR activation, which is the main subject of this paragraph. The sentences that immediately follow this line all refer to studies that have previously described how pain signaling could be terminated peripherally via adrenergic mechanisms. Indeed, both NET and α2-AR are known to be expressed centrally in the level of the spinal cord where sensory projections terminate. As a matter of fact, the analgesic effect of NET inhibitors is mostly known to function centrally, as we write in this sentence: "Most of the evidence for analgesic activity of NET inhibitors involves the central processing in the locus coeruleus and the spinal cord." (Page 31, second paragraph)

We also described the central processing of pain through α2-AR in the Introduction: "Briefly, NE acts in the spinal cord as an inhibitory modulator of nociceptive processing through the activation of α2-adrenergic receptors (α2-AR). NE is taken up into the releasing neuron after having stimulated pre- and post-synaptically located G protein-coupled receptors (GPCRs). Importantly, presynaptic α2-ARs negatively regulate NE release, but an increase in synaptic NE stimulates postsynaptic α2-AR to inhibit calcium channels, which, in turn, reduces the release of excitatory neurotransmitters. Based on this mechanism, certain NET-targeting antidepressants are effective in treating co-incident pain and depressive disorders." (Page 5, second paragraph)

Reviewer 1, continued:

Summary of Key Results:

1) The structure of a chi-conotoxin bound to NET is presented.

2) Similar to previous work on chi-conotoxins, the peptide presented here also exhibit antinociceptive activity.

Originality and significance:

1) Again, the structure of the transporter bound to a ligand is a significant advancement in the field of conotoxins and in NET research.

2) Key results were mainly consistent with previously published work.

Response: We thank the reviewer for summarizing and acknowledging the quality of our work.

Data & methodology: validity of approach, quality of data, quality of presentation

1) Rationale for using a specific method needs to be presented.

a. Why is the peptide delivered subcutaneously rather than IT when MrIa was shown to be more effective via IT.

Response: As we stated above, our objective is to explore alternatives to MrIA. Given that AoIA produces antinociceptive activity using subcutaneous administration, which is a less invasive route compared to intrathecal delivery, we do not deem it necessary to further test intrathecal effects as the subcutaneous poses a clear advantage over the intrathecal route as outlined in more detail above.

b. Why was NE not used in the transporter assay.

Response: MPP^+ is a known substrate of monoamine transporters and is a suitable and - more importantly – stable alternative to NE in our uptake assays.

c. Why was post mortem analysis of the spinal cord not performed considering that this tissue is where chi-conotoxins have their most potent effect.

Response: We believe that the post-mortem analysis of brain tissue is sufficient since the spinal cord is similarly shielded by the blood-brain barrier.

2) Quality of data and presentation is satisfactory.

Appropriate use of statistics and treatment of uncertainties

1) This is satisfactory.

Response: Again, we thank the reviewer for acknowledging the quality of our work.

Conclusions: robustness, validity, reliability

1) The main conclusion that the peptide acts primarily on the somatosensory circuit needs more experimental support.

a. The activity of the peptide at the spinal level was not measured thus the possibility that it is more potent and thus exerts most of its activity in the spinal cord cannot be eliminated at this point. Work in the related peptide, MrIa has demonstrated the IT delivery is more effective at attenuating nociception thus the same can be said for the peptide described in this work.

Response: This is indeed a complex question. While we recognize its importance, we currently lack the experimental framework to address it comprehensively within the revision timeline. We have now added new text to the discussion section and included it also in the "limitations of the study" section which Reviewer 3 had also suggested in their comments to authors. While the mode of action is not entirely clear at the present stage, we are convinced that our explanation, which we have now put forward, may serve as a starting point to explore this interesting activity of a subcutaneously administered antinociceptive peptide. The newly added text can be found at the end of the Discussion section, within the two last paragraphs (Pages 31 – 32).

b. It is interesting to explain, based on the structure presented in the paper, why the peptide is more potent than MrIA.

Response: The molecular basis of the increased potency of AoIA in comparison to MrIA and its globular isomer AoIA^{glob} presents an interesting question that we are pursuing concurrently as a follow-up project, involving computational pharmacology, mutagenesis, and other wet lab approaches.

Suggested improvements: experiments, data for possible revision.

References: appropriate credit to previous work?

1) Please state in the result section how the peptide sequence was obtained. Is this from the Gupta paper?

Response: The sequence was indeed obtained from Gupta's work which was already cited in the original text. This is more explicitly stated in the revised text: "The peptide originally isolated by Gupta et al. from *Conus araneosus* with the sequence RCCGYKMCHOC, where O stands for 4-hydroxyproline (Hyp), was found to possess the same disulfide connectivity as other χ-conotoxins. (page 5, last paragraph)

Clarity and context: lucidity of abstract/summary, appropriateness of abstract, introduction and conclusions

1) This is satisfactory. Please see above comments for improving the conclusion

Response: We would like to again thank the reviewer for acknowledging the quality of our work.

REFERENCES to Reviewer 1:

Abbott, F. V., & Melzack, R. (1982). Brainstem lesions dissociate neural mechanisms of morphine analgesia in different kinds of pain. *Brain research*, *251*(1), 149–155. https://doi.org/10.1016/0006-8993(82)91282-3

Bannon, A. W., & Malmberg, A. B. (2007). Models of nociception: hot-plate, tail-flick, and formalin tests in rodents. *Current protocols in neuroscience*, *Chapter 8* https://doi.org/10.1002/0471142301.ns0809s41

Bryan-Lluka, L. J., Bönisch, H., & Lewis, R. J. (2003). chi-Conopeptide MrIA partially overlaps desipramine and cocaine binding sites on the human norepinephrine transporter. *The Journal of biological chemistry*, *278*(41), 40324–40329. https://doi.org/10.1074/jbc.M213101200

D'Amour, F. E., & Smith, D. L. (1941). A method for determining loss of pain sensation. *The Journal of pharmacology and experimental therapeutics*, *72*(1), 74-79.

Fischer, M., Carli, G., Raboisson, P., & Reeh, P. (2014). The interphase of the formalin test. *Pain*, *155*(3), 511–521. https://doi.org/10.1016/j.pain.2013.11.015

Fischer, M. J., Soller, K. J., Sauer, S. K., Kalucka, J., Veglia, G., & Reeh, P. W. (2015). Formalin evokes calcium transients from the endoplasmatic reticulum. *PloS one*, *10*(4), e0123762. https://doi.org/10.1371/journal.pone.0123762

Hoffmann, T., Klemm, F., I Kichko, T., Sauer, S. K., Kistner, K., Riedl, B., Raboisson, P., Luo, L., Babes, A., Kocher, L., Carli, G., Fischer, M. J. M., & Reeh, P. W. (2022). The formalin test does not probe inflammatory pain but excitotoxicity in rodent skin. Physiological reports, 10(6), e15194. https://doi.org/10.14814/phy2.15194

Hu, T., Yu, Z., Zhao, J., Meng, Y., Salomon, K., Bai, Q., Wei, Y., Zhang, J., Xu, S., Dai, Q., Yu, R., Yang, B., Loland, C. J., & Zhao, Y. (2024). Transport and inhibition mechanisms of the human noradrenaline transporter. *Nature*, *632*(8026), 930–937. https://doi.org/10.1038/s41586-024-07638-z

Irwin, S., Houde, R. W., Bennett, D. R., Hendershot, L. C., & Seevers, M. H. (1951). The effects of morphine methadone and meperidine on some reflex responses of spinal animals to nociceptive stimulation. *The Journal of pharmacology and experimental therapeutics*, *101*(2), 132–143.

Iyengar, S., Webster, A. A., Hemrick-Luecke, S. K., Xu, J. Y., & Simmons, R. M. (2004). Efficacy of duloxetine, a potent and balanced serotonin-norepinephrine reuptake inhibitor in persistent pain models in rats. *The Journal of pharmacology and experimental therapeutics*, *311*(2), 576–584. https://doi.org/10.1124/jpet.104.070656

Paczkowski, F. A., Sharpe, I. A., Dutertre, S., & Lewis, R. J. (2007). chi-Conotoxin and tricyclic antidepressant interactions at the norepinephrine transporter define a new transporter model. *The Journal of biological chemistry*, *282*(24), 17837–17844. https://doi.org/10.1074/jbc.M610813200

Sharpe, I. A., Gehrmann, J., Loughnan, M. L., Thomas, L., Adams, D. A., Atkins, A., Palant, E., Craik, D. J., Adams, D. J., Alewood, P. F., & Lewis, R. J. (2001). Two new classes of conopeptides inhibit the alpha1-adrenoceptor and noradrenaline transporter. *Nature neuroscience*, *4*(9), 902–907. https://doi.org/10.1038/nn0901-902

Reviewer 2

The noradrenaline transporter (NET) is responsible for clearing noradrenaline from the synaptic cleft, thereby terminating its action on postsynaptic receptors. NET inhibitors have shown considerable promise in the treatment of depression and pain. In this study, Belleza et al. report the identification of a novel conotoxin, ArXA, based on its sequence similarity to previously characterized conotoxins. ArXA demonstrates greater potency in inhibiting NET compared to MrIA. Furthermore, the cryo-EM structure of the NET–ArXA complex provides valuable insight into the molecular mechanism underlying ArXA-mediated inhibition. ArXA also exhibits antinociceptive effects in an inflammatory pain model. Overall, the manuscript is well-written and logically organized. However, several concerns need to be addressed before it can be considered for publication.

Response: We thank the reviewer for the positive comments on our manuscript and thorough feedback.

Major:

1. The previously published structure of NET bound to MrIA, which is closely related to the structure reported in this study, should be discussed in the Introduction. A comparison between the two structures should be presented in the main figures and described in the main text.

Response: The previously published structure of $MrIA^{EM}$ bound to NET is now mentioned in the Introduction: "More recently, the structure of a synthetic analogue of MrIA bound to NET, χ-$MrIA^{EM}$, was solved using cryogenic electron microscopy (cryo-EM)." (Page 4), and this is discussed further in the Main Text. A direct comparison between the two structures, NET:$MrIA^{EM}$ vs NET:AoIA, is now presented in Figure 4 Panel K and an accompanying paragraph has been added to the Main Text to discuss their similarities (Page 17, last paragraph).

2. While previous studies have characterized MrIA and related toxins as allosteric inhibitors, this work proposes a mixed competitive/noncompetitive mode of inhibition for ArXA. The evidence for competitive inhibition is based on a slight increase in Km at higher ArXA concentrations; however, this difference is minimal and maybe not statistically significant. Although the structural data show that ArXA extends into the S1 binding site and partially overlaps with the amine moiety of norepinephrine (NE), the transporter's dynamic nature suggests that minor conformational adjustments could still permit simultaneous binding of ArXA and NE. Therefore, the conclusion that ArXA competes with NE is not fully convincing. Additional experiments with higher ArXA concentrations should be performed to determine whether the increase in Km reaches statistical significance, and molecular dynamics simulations are recommended to further evaluate the structural compatibility of ArXA and NE.

Response: We have repeated our uptake experiments to better characterize the mode of NET inhibition with higher concentrations of the peptide AoIA, up to 10 μM. We also tested at 25 μM, but the resulting uptakes were too low to obtain meaningful information. With our new data, the difference in Km between low (0.1, 0.5, and 1 μM) to high (5, and 10 μM) AoIA concentrations became even more apparent, as we describe in the Main Text: "This is substantiated by the observed

incremental concentration-dependent decrease in V_{max} accompanied by an initial constant K_m: going from 1.9 µM in the control buffer to 1.6 – 1.8 µM in the presence of 0.1 – 1 µM AoIA, followed by an increase in K_m values at higher concentrations (4.1 µM and 7.4 µM at AoIA concentrations 5 µM and 10 µM, respectively) (Figure 2C, Table S5)." (Page 9)

We would also like to point out that the previous study by Sharpe et al. (2001) that described the non-competitive mode of NET uptake inhibition by MrIA reported only a single concentration, 1 µM, which would not have been sufficient to produce the competitive mode that we now observe with AoIA. In addition, we have performed molecular dynamics simulations to investigate the interactions of AoIA and NE in the central binding site. Accordingly, the dynamic nature of the NET:AoIA complex shows the structural incompatibility of $K6^{AoIA}$ simultaneously occupying the same space as the substrate NE within the central binding site, which is in support of a competitive mode inhibition. We describe now in the Main Text: "To further ascertain the competitive nature of NE and AoIA binding by spatial overlap, we carried out MD simulations. The final hypothetical structure of the MD simulation reflects the competitive nature as it leads to a high energy conformation in which several key features of high-affinity binding to NET are broken." (Page 18, last paragraph, Figures 4I-J)

3. Since ArXA demonstrates greater potency than MrIA in inhibiting NET transport, the authors should elaborate on the underlying reasons for this difference, drawing on insights from structural analysis and mutagenesis experiments.

Response: Regarding the molecular basis of the potency of AoIA in comparison to MrIA, this is a more challenging (and also interesting) question that we are indeed pursuing concurrently as a follow-up project, involving computational pharmacology, mutagenesis, and other wet lab approaches. Thank you for pointing this out.

Beyond the structure and modelling based approach as used in the first submission of the manuscript, we will make extensive use of MD simulations to address this question by comparing dynamics, interaction, forces and energies of NET:AoIA and NET:MrIA complexes. We expect to extract from these data the molecular origin of the higher affinity.

4. The authors evaluated the antinociceptive effect of ArXA in male mice. To better contextualize these findings, they should report the sequence similarity between mouse and human NET and clarify ArXA's potency for mouse NET. Although they ruled out ArXA's action on opioid receptors and TRPV1, its antinociceptive effect could still involve other targets, which the authors should address or exclude.

Response: We have included a sequence comparison of mouse and human NET as Extended Data Figure S8 (the sequence identity is 94%) and in the discussion: "Mouse and human NET have a 94% sequence identity (Figure S8), which makes it a suitable model for translating our *in vitro* results." (Page 29, last paragraph). We have performed electrophysiological experiments to assess AoIA's interaction with voltage-gated Nav1.7 and Nav1.8 channels, as well as BRET-based biosensor studies assessing any potential agonism at the alpha2A-adrenergic receptor, thereby strengthening the specificity profile of AoIA: please see Figure 3, and the accompanying description on Pages 10-11.

Minor

1. The two-dimensional 15N HSQC spectrum of ArXA, along with its assignment, should be provided in the supplementary figures.

Response: This has been added as Figure S1.

2. Please clarify what the dashed lines in Figure 3 represent. If they indicate hydrogen bonds, why are dashed lines shown between sulfur and oxygen atoms in Figure 3G?

Response: Thank you for pointing this out. Indeed, dashed lines represent hydrogen bonds. We have updated the figure and removed the dashed lines between S and O atoms.

3. The side chains and labels in Figure 4 are unclear and should be redrawn for better readability. Additionally, a sequence alignment of NET, SERT, and DAT should be included in the supplementary materials.

Response: The figure has been redrawn for clarity: please see Figure 5. Sequence alignment of NET with DAT and SERT has been included in Extended Data Figure S9.

4. The scale bars are missing in the 2D averages shown in Extended Figure 2A and should be added. A model to map the FSC should also be included.

Response: The scale bars have been redrawn for better clarity: please see Extended Figure S5A. The model to map the FSC is included in the same figure, Panel C.

The IC50 values for the competition curves should be summarized in an extended table.

Response: A table containing the IC_{50} values has been added to the Extended Data (Table S4).

Reference for Reviewer 2:

Sharpe, I. A., Gehrmann, J., Loughnan, M. L., Thomas, L., Adams, D. A., Atkins, A., Palant, E., Craik, D. J., Adams, D. J., Alewood, P. F., & Lewis, R. J. (2001). Two new classes of conopeptides inhibit the alpha1-adrenoceptor and noradrenaline transporter. *Nature neuroscience*, *4*(9), 902–907. https://doi.org/10.1038/nn0901-902

Reviewer 3

In this study, the authors synthesized and characterized χ-conotoxin (χ-ArXA), a peptide that inhibits the norepinephrine transporter (NET) without affecting opioid receptors or TRP channels (TRPA1 and TRPV1). Importantly, they resolved the cryo-EM structure of the human NET:χ-ArXA complex at high resolution, revealing an atypical binding mode that engages both the central binding site and the outer vestibule of the transporter. Furthermore, subcutaneous administration of χ-ArXA produced a significant antinociceptive effect in a mouse formalin-induced inflammatory pain model. These findings are both novel and significant, providing new mechanistic insights into χ-conotoxins and highlighting their potential as leads for the development of non-opioid analgesics.

Response: We thank the reviewer for their thorough feedback and positive comments.

Points for consideration:

1. Introduction: ω-conotoxin MVIIA is approved for clinical use as the intrathecally administered drug ziconotide). A comparison between ω-conotoxin and χ-conotoxin (χ-ArXA) in their analgesic efficacy and side effect profile will strengthen this study.

Response: We agree that a putative translational value of a direct comparison between AoIA and ziconotide could be of interest. Our study, however, focuses on the very first structural and pharmacological characterization of AoIA, with great emphasis on elucidating its primary mechanistic target through an extensive battery of in vitro assays, molecular dynamics simulations, as well as its analgesic efficacy *in vivo*. Ziconotide functions through a different mechanism, and we do cite a number of studies that describe it. A more extensive comparison of the differences in analgesic efficacy and side effect profiles among different pharmacological families of conotoxins (e.g., χ vs ω) would be beyond the scope of our study.

2. It is impressive to solve cryo-EM) structure of the human NET:ArXA complex and demonstrate in vitro activity of NET inhibition. However, ArXA may produce antinociception via additional targets in vivo. I wonder if α2-AR antagonist or knockout can block the analgesic effects of ArXA.

Response: We have performed additional experiments to explore other potential targets for antinociception. Firstly, BRET-based biosensor studies assessed any potential agonism at the alpha2A-adrenergic receptor, and we found no such effect for AoIA (Figure 3B-C). In addition, electrophysiological experiments demonstrated the lack of effect of AoIA on voltage-gated Nav1.7 and Nav1.8 channels (Figure 3F-H). While there might still be additional targets *in vivo* that we have not assessed, we believe our cell-based experiments have already ruled out a substantial number of targets that could explain mechanistically how the antinociception is elicited by AoIA in vivo. This includes a lack of agonistic or antagonistic effect on the alpha2a-adrenergic receptor.

3. NET inhibitors are used to treat neuropathic pain. It will facilitate translation if the authors can assess a neuropathic pain model. Subcutaneous injection may only produce transient pain relief.

Response: As correctly pointed out, we agree that testing AoIA in a neuropathic pain model would strengthen the manuscript, and we felt motivated to explore this avenue further. However, we found this too challenging to be included in this current project due to the following constraints:

(i) The lack of an approved animal experimentation protocol within our consortium, and

(ii) The extensive training required for such experiments (the technique is currently not in use within our consortium and not anywhere near to anyone of us).

(iii) Resource and feasibility constraints: Compared to inflammatory pain models, neuropathic pain models require longer experimental timelines, larger animal cohorts due to higher variability, and tight regular observation due to severeness of the models, making them significantly more resource-intensive.

We therefore discuss this as a limitation of the current manuscript (Pages 30-32).

4. Limitations of this study should be discussed.

Response: We have added a description of the limitations of our study (Page 32, last paragraph), especially pertaining to our *in vivo* experiments. We acknowledge that investigating the antinociceptive activity of AoIA via intrathecal versus subcutaneous administrations, as well as in a neuropathic pain model, would add substantial, incredibly valuable information about our newly characterized peptide. However, we would like to stress that the primary objective of our *in vivo* study was to demonstrate:

(a) the proof of action of AoIA as an active, antinociceptive non-opioid agent,

(b) the significant antinociceptive efficacy after systemic (i.e., s.c.) administration in a preclinical inflammatory pain model; s.c. injections offer clear benefits to intrathecal injections, such as sustained release and convenience for self-administration, effectiveness, better safety and usually well-tolerated application at reduced costs and also time saving.

(c) the antinociceptive activity of AoIA without adverse motor effects, thus a favourable risk-benefit ratio,

(d) a comparable antinociceptive efficacy of AoIA to the gold-standard analgesic morphine in an inflammatory pain model (chronic phase)

(e) the clear benefits of the AoIA vs. MrIA as an effective nonopioid analgesic, based on AoIA significant antinociceptive efficacy after systemic s.c. administration vs. the lack of efficacy of MrIA in preclinical inflammatory pain, paralleled by the absence of unwanted sedative behaviours of AoIA.

Dear Editor, dear Reviewers,

Many thanks for your careful examination of our manuscript. We are pleased that you have found our added revisions sufficient and our responses to your previous comments satisfactory. In this document, we provide our response to additional comments from the Reviewers.

Oliver Belleza, Zhang Heng, Eric Xu, Harald Sitte, on behalf of the entire team

Reviewer #1 (Remarks to the Author):

The authors presented a structure of chi-conotoxin AoIa bound to human NET. Similar to previously published article the data presented here described key interactions between the transporter and the toxin that could explain the high degree of selectivity of chi-AoIa and most likely as yet to be discovered chi-conotoxins towards NET.

Another important discovery in this paper is the apparent antinociceptive activity of the conotoxin presumably from targeting NET in the peripheral nervous system.

The authors have satisfactorily addressed my comments and I recommend this research for publication

Response: We thank Reviewer 1 for their comments and positive evaluation of our manuscript.

Reviewer #2 (Remarks to the Author):

The authors have addressed most of my concerns. I have a minor suggestion regarding the structural model: the clash score is unusually high, which may be due to misplacement of water molecules that create steric clashes with nearby residues. In cryo-EM structures, particularly at moderate resolution, water molecules should be modeled cautiously, as spherical densities can represent noise and often appear throughout the map. In Figure 4A, the density assigned to some water molecules appears too bulky to correspond to water. I recommend that most water molecules be removed or re-evaluated in the structural model.The Figure 4 should be updated accordingly.

Response: We thank Reviewer 2 for their comments and suggestions. The water molecules have been re-evaluated. The corresponding figure, Figure 4, has been updated accordingly.

Reviewer #3 (Remarks to the Author):

The revision is improved. I have no further comments.

Response: We thank Reviewer 3 for their positive comments.
